# miR-92a-3p Promoted EMT *via* Targeting LATS1 in Cervical Cancer Stem Cells

**DOI:** 10.3389/fcell.2021.757747

**Published:** 2021-11-18

**Authors:** Shuangyue Liu, Liping Chu, Mingzhu Xie, Lisha Ma, Hongmei An, Wen Zhang, Jihong Deng

**Affiliations:** Department of Gynecology, Kunming Maternity and Child Care Hospital, Kunming, China

**Keywords:** miR-92a-3p, larger tumor suppressor, cervical cancer, E-cadherin, EMT

## Abstract

miR-92a-3p (microRNA-92a-3p) has been reported to be dysregulated in several cancers, and as such, it is considered to be a cancer-related microRNA. However, the influence of miR-92a-3p on biological behaviors in cervical cancer (CC) still remains unclear. Quantitative real-time PCR was used to detect miR-92a-3p levels in CC stem cells. Here, Cell Counting Kit-8 (CCK8) assay, Transwell cell invasion assay and flow cytometry assay were used to characterize the effects that miR-92a-3p and large tumor suppressor l (LATS1) had on proliferation, invasion and cell cycle transition. The luciferase reporter gene assay was used to verify the targeting relationship between miR-92a-3p and LATS1. Western Blotting was used to investigate the related signaling pathways and proteins. Data from The Cancer Genome Atlas (TCGA) showed that miR-92a-3p was upregulated in CC tissues and closely associated with overall survival. miR-92a-3p promoted proliferation, invasion and cell cycle transition in CC stem cells. The luciferase reporter assay showed that miR-92a-3p bound to the 3′-untranslated region (3′-UTR) of the LATS1 promoter. LATS1 inhibited proliferation, invasion and cell cycle transition. Results measured by Western Blotting showed that LATS1 downregulated expressions of transcriptional co-activator with PDZ-binding motif (TAZ), vimentin and cyclin E, but upregulated the expression of E-cadherin. Re-expression of LATS1 partly reversed the effects of miR-92a-3p on proliferation, invasion and cell cycle transition, as well as on TAZ, E-cadherin, vimentin, and cyclin E. miR-92a-3p promoted the malignant behavior of CC stem cells by targeting LATS1, which regulated TAZ and E-cadherin.

## Introduction

Cervical cancer (CC) is one of the most common cancers in females and a major cause of cancer-related mortalities (Siegel et al., 2019). CC is associated with a high risk of mortality due to tumor metastasis and recurrence. Therefore, identification of biomarkers which could predict CC progression and novel therapeutic targets is of great importance.

It has been reported that more than 60% of the human protein-coding genes are regulated by microRNAs, which bind to the 3′-UTR of target mRNAs. The microRNAs are involved in various physiological and pathological processes, including the malignant tumor progression (Sherafatian and Arjmand, 2019). miR-92a-3p is a member of miR-17-92 clusters, and it has been found to be overexpressed in several cancers. miR-92a-3p was found to stimulate VEGF and angiogenesis in ovarian cancer cells (Guo et al., 2017), and be significantly upregulated in serum samples of patients with early stage hepatocellular carcinoma (Zhang et al., 2017). miR-92a-3p could also serve as a serum biomarker for the recurrence of colon cancer patients after adjuvant chemotherapy (Conev et al., 2015). It has also been reported that miR-92a functioned as an onco-miRNA in CC (Zhou et al., 2015; Luo et al., 2017). However, the biological effect, regulatory network and potential mechanism of miRNAs in malignant tumors are complex. Therefore, further characterization of miR-92a-3p in CC is necessary.

In this study, we investigated the regulatory effect of miR-92a-3p on the biological role in CC, as well as the underlying mechanism of Hippo signaling. Our studies testified that miR-92a-3p promoted malignant behaviors of CC stem cells by targeting the large tumor suppressor 1 (LATS1) and its downstream transcriptional co-activator with PDZ-binding motif (TAZ). The results, therefore, suggested that miR-92a-3p might be a biomarker of CC patients.

## Materials and Methods

### Cell Culture and Cell Transfection

The cells were cultured in Dulbecco’s modified eagle medium (DMEM) (Gibco, Gaithersburg, MD, United States) with 10% fetal bovine serum (Gibco) at 37°C. The cells were passaged every 2–3 days. miR-92a-3p mimics control and miR-92a-3p inhibitor control were synthesized by Ribobio (Guangzhou, China), and transfected into cells with DharmaFECT 1 (VWR, Radnor, PA, United States) in accordance with the instruction for use provided by the manufacturer. The pCMV6-LATS1 plasmid and empty plasmid were purchased from OriGene Technologies (Rockville, MD, United States), and transfected into cells with Lipofectamine 3000 (Thermo Fisher Scientific, Waltham, MA, United States) according to the manufacturer′s instruction for use.

### Quantitative Real-Time PCR

Quantitative real-time PCR (qRT-PCR) was used to determine mRNA levels. Total RNA of differently treated cells was prepared with TRIZOL (Thermo Fisher Scientific) according to the manufacturer’s instruction for use. The RNA concentration was quantified with NanoDrop 2000c (Thermo Fisher Scientific). The cDNA was obtained by reverse transcription with the PrimerScript RT Reagent Kit (VWR) according to the manufacturer’s instruction for use. SYBR Green PCR Master Mix (Bio-Rad, Hercules, CA, United States) and ABI 7500 real-time PCR system (Thermo Fisher Scientific) were adopted to analyze the RT-PCR products. U6 was used as an internal control. The mRNA levels were measured according to the 2^−△△Ct^ method (Livak and Schmittgen, 2001). All experiments were repeated for three times.

### The Cell Counting Kit-8 Assay

The viable cell mass was measured with the Cell Counting Kit-8 (CCK8) Assay (Bimake, Houston, TX, United States). The CC stem cells (1 × 10^5^) were transfected with miR-92a-3p mimic/inhibitor and seeded in 96-well plates. The cells were then cultured in an incubator with 5% CO_2_ at 37 °C for 1, 2, 3, 4, and 5 days respectively. Totally 10 μl CCK8 solution was added into each well, and the cells were cultured for another 2 h. The absorbance was finally determined at 490 nm with a microplate reader.

### Transwell Invasion Assay

The invasion assay was performed with a Transwell chamber (Corning, Corning, NY, United States) and covered with Matrigel (BD Biosciences, San Jose, CA, United States). Serum-free medium and complete medium were added in the upper and lower chambers, respectively. The cells (1 × 10^5^) were seeded in the upper chamber and cultured for 36 h in an incubator with 5% CO_2_ at 37°C. Non-invasive cells were removed, and the cells that entered the lower chamber were stained with hematoxylin and counted with a microscope (BX53; Olympus, Tokyo, Japan) at the magnification of ×400.

### Flow Cytometry Assay

The CC stem cells were seeded into a 6-well plate and cultured in an incubator with 5% CO_2_ at 37°C for 48 h. The cells were then resuspended with phosphate-buffered saline (Thermo Fisher Scientific) and fixed with precooled alcohol (75%). Totally 300 μl of propidium iodide was then added into the tube and the mixed solution was incubated for 15 min in the darkness. The cell cycle was determined with the flow cytometry.

### Western Blotting

Western Blotting was used to determine the levels of proteins. Briefly, the cells were harvested with lysis buffer containing 1% protease inhibitor and phosphatase inhibitors. Totally 30 μg of total protein was loaded onto a 10% SDS-PAGE gel, and after electrophoresis, the protein was transferred to polyvinylidene fluoride (PVDF) membranes, which was to be blocked with 5% bovine serum albumin and incubated with primary antibodies of cyclin E (4,129; 1:1,000; Cell Signal Technology, Danvers, MA, United States), E-cadherin (3,195; 1:1,000; Cell Signal Technology), vimentin (3,932; 1:1,000; Cell Signal Technology), TAZ (83,669; 1:1,000; Cell Signal Technology), LATS1 (9,153; 1:1,000; Cell Signal Technology), and β-actin (4,970; 1:1,000; Cell Signal Technology) at 4°C for overnight. The PVDF membranes were then washed with TTBS buffer and probed with horseradish peroxidase (HRP)-conjugated secondary antibody (7,074/7,076; 1:2,000; Cell Signal Technology) at 37°C for 2 h. The immunosignal substance was detected with the SuperSignal West Dura Extended Duration Substrate Kit (Thermo Fisher Scientific), and the resulting images were captured with an imaging system (DNR BioImaging System, Jerusalem Israel).

### The Luciferase Reporter Assay

Wild type and mutant sequences of LATS1 3′-UTR with putative miR-92a-3p binding sites were synthesized and sub-cloned into the luciferase vector to generate wild type and mutant luciferase reporters. Then, 100 ng of the luciferase vector was co-transfected with the miR-92a-3p mimic or negative control with Lipofectamine 3000 (Invitrogen, Carlsbad, CA, United States). The luciferase activities were assessed with a Dual-luciferase Reporter Assay Kit (Promega, Madison, WI, United States) according to the manufacturer’s protocol.

### The Cancer Genome Atlas Database Analysis

TCGA is a landmark cancer genomics program, which molecularly characterizes over 20,000 primary cancer and matched normal samples including 33 different cancer types. The miR-92a-3p mRNA expression profiles for CC patients were obtained from TCGA (https://www.cancer.gov/about-nci/organization/ccg/research/structural-genomics/tcga). Overall survival of CC patients was also downloaded from TCGA data portal. Data acquirement and application were performed in accordance with TCGA publication guidelines and data access policies.

### Statistic Analysis

SPSS statistical software for Windows, version 22.0 (SPSS, Chicago, IL, United States) was used for the statistic analysis. The data were compared with the *t*-test and one-way analysis of variance. The bilateral 95% confidence interval was used for all tests, and a value of *p* < 0.05 represented statistical significance.

## Results

### miR-92a-3p was Upregulated in CC and Associated With a Poor Prognosis

**FIGURE 1:**
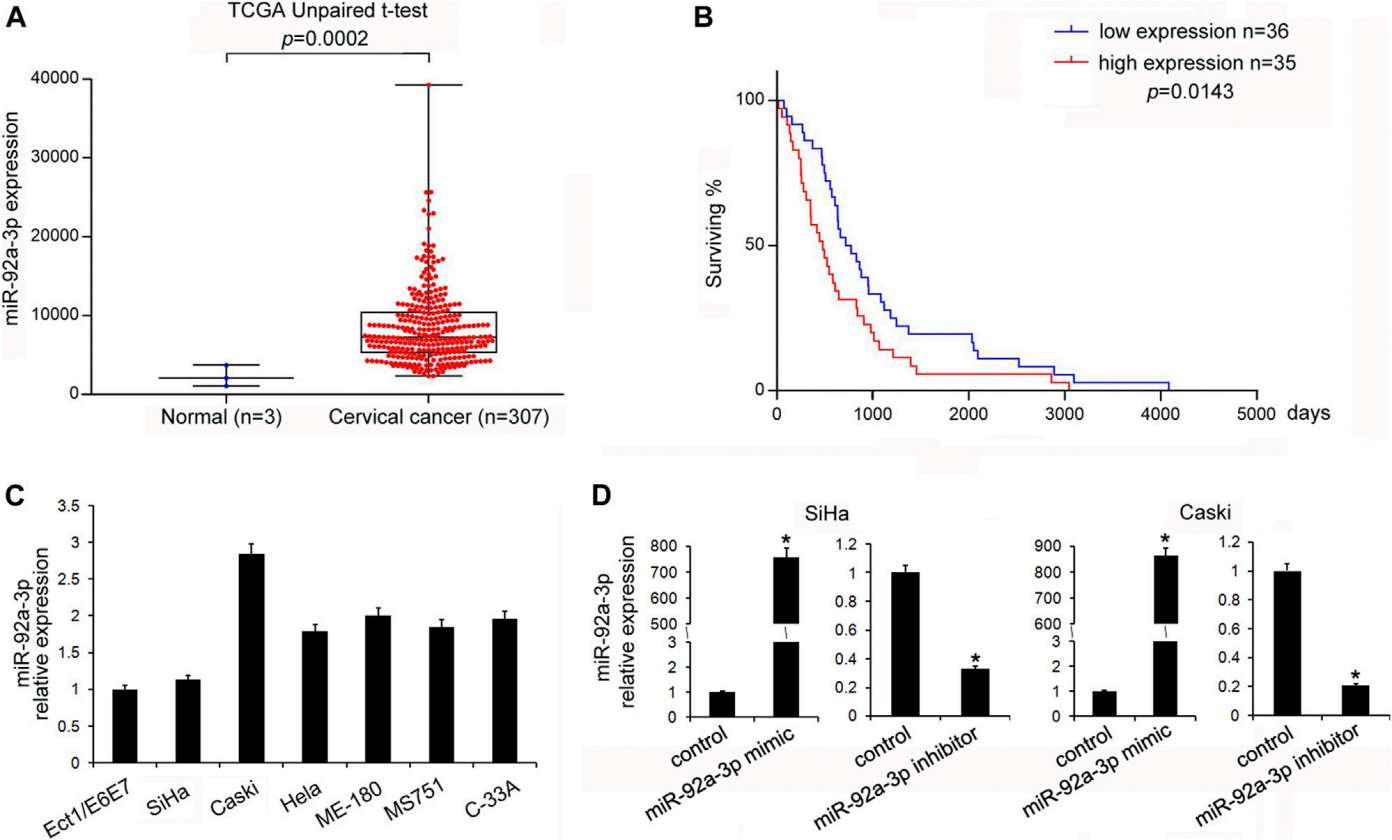
miR-92a-3p was upregulated in cervical cancer. **(A)** Analysis of The Cancer Genome Atlas data showed that miR-92a-3p expression in cervical cancer (CC) tissues was higher than that in adjacent normal tissues (*t*-test, *p* = 0.0002). **(B)** Kaplan-Meier survival curves showed that patients with low miR-92a-3p expressions had better overall survival of than those with high miR-92a-3p expressions (log-rank test, *p* = 0.0143). **(C)** miR-92a-3p expressions in Ect1/E6E7, Caski, SiHa, Hela, ME-180, MS751, and C-33A cell lines were examined using quantitative real-time PCR. miR-92a-3p levels in CC stem cells were higher than those in normal cervical cell lines (Ect1/E6E7). **(D)** RT-qPCR showed that miR-92a-3p expressions increased after miR-92a-3p mimic transfection in SiHa and Caski cells. The miR-92a-3p expression decreased after transfection of miR-92a-3p inhibitor in Caski cells. *p < 0.05.

We analyzed miR-92a-3p expression profiles of 307 cases of CC patients from the TCGA database. The results showed that the CC tissues had higher miR-92a-3p expression levels than adjacent normal tissues (Figure 1A, unpaired *t*-test, *p* = 0.0002). We also analyzed the correlations of miR-92a-3p levels with patients’ overall survival. Kaplan-Meier survival analysis showed that the overall survival of CC patients with low miR-92a-3p levels was longer than those with high miR-92a-3p levels (Figure 1B, log-rank test, *p* = 0.0143).

### miR-92a-3p Facilitated Proliferation, Invasion, and Cell Cycle Transition in CC Stem Cells

**FIGURE 2:**
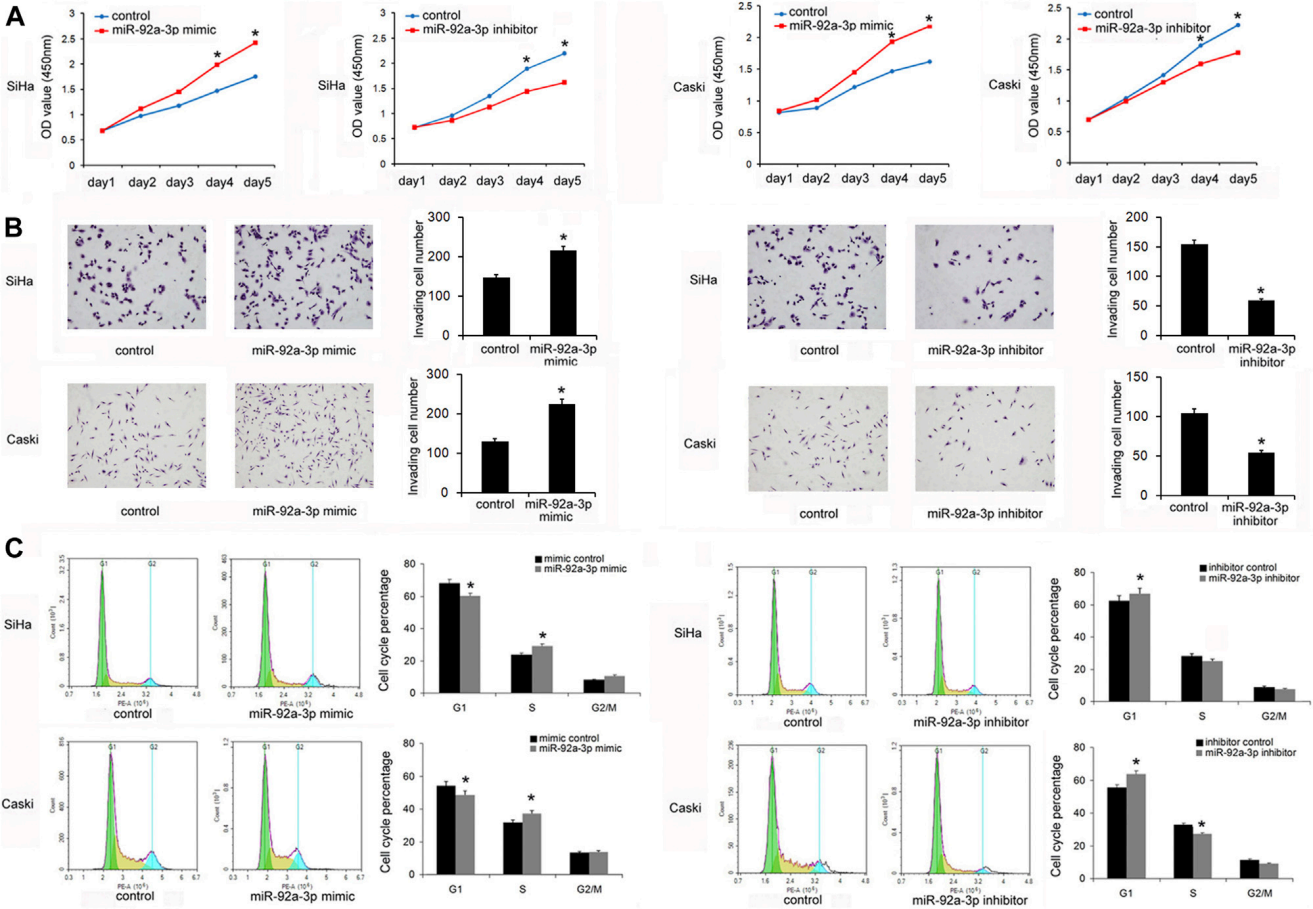
miR-92a-3p promoted proliferation, invasion, and cell cycle transition in cervical cancer stem cells. **(A)** The Cell Counting Kit-8 assay showed that cell growth in SiHa and Caski cells was increased after transfection of the miR-92a-3p mimic. The cell growth was decreased in SiHa and Caski cells transfected with the miR-92a-3p inhibitor. **(B)** The Transwell invasion assay showed that the invading number increased after miR-92a-3p overexpression in SiHa and Caski cells. miR-92a-3p inhibitor transfection decreased the invading SiHa and Caski cell numbers. **(C)** Flow cytometry results showed that the G1 percentage decreased, and the S percentage increased in SiHa and Caski cells after miR-92a-3p overexpression. miR-92a-3p inhibition increased the percentage of cells in G1 phase and reduced the percentage of cells in S phase. *p < 0.05.

The levels of miR-92a-3p in a normal cervical cell line Ect1/E6E7 and several CC stem cells lines (CaSki, SiHa, Hela, ME-180, MS751, and C-33A) were examined. Figure 1C shows that in five out of six CC cell lines, the levels of miR-92a-3p were higher in 5/6 CC cell lines than that in Ect1/E6E7, which was consistent with its expression pattern in CC tissues. Transfections of the miR-92a-3p mimic and inhibitor were conducted in SiHa and CaSki cell lines, respectively. Figure 1D shows that miR-92a-3p mimic transfection upregulated its endogenous levels, while miR-92a-3p inhibitor transfection downregulated its expression in both SiHa and CaSki cells. The CCK8 assay was then used to determine its role in CC cell growth. The results showed that miR-92a-3p overexpression promoted proliferation, while miR-92a-3p inhibition decreased the growth rate of SiHa and CaSki cells (Figure 2A). The Transwell invasion assay showed that the number of cells passing through the Transwell membrane increased in the miR-92a-3p mimic-transfected SiHa and CaSki cells. miR-92a-3p inhibitor transfection decreased the invading SiHa and CaSki cell numbers (Figure 2B). Changes of the cell cycle were also examined. Figure 2C shows that the miR-92a-3p mimic transfection decreased the percentage in G1 phase but increased it in S phase in the SiHa and CaSki stem cells, however, miR-92a-3p inhibitor was on the contrary, which suggested that miR-92a-3p facilitated cell cycle progression in CC stem cells.

### miR-92a-3p Targeted LATS1 Directly in CC Stem Cells

**FIGURE 3:**
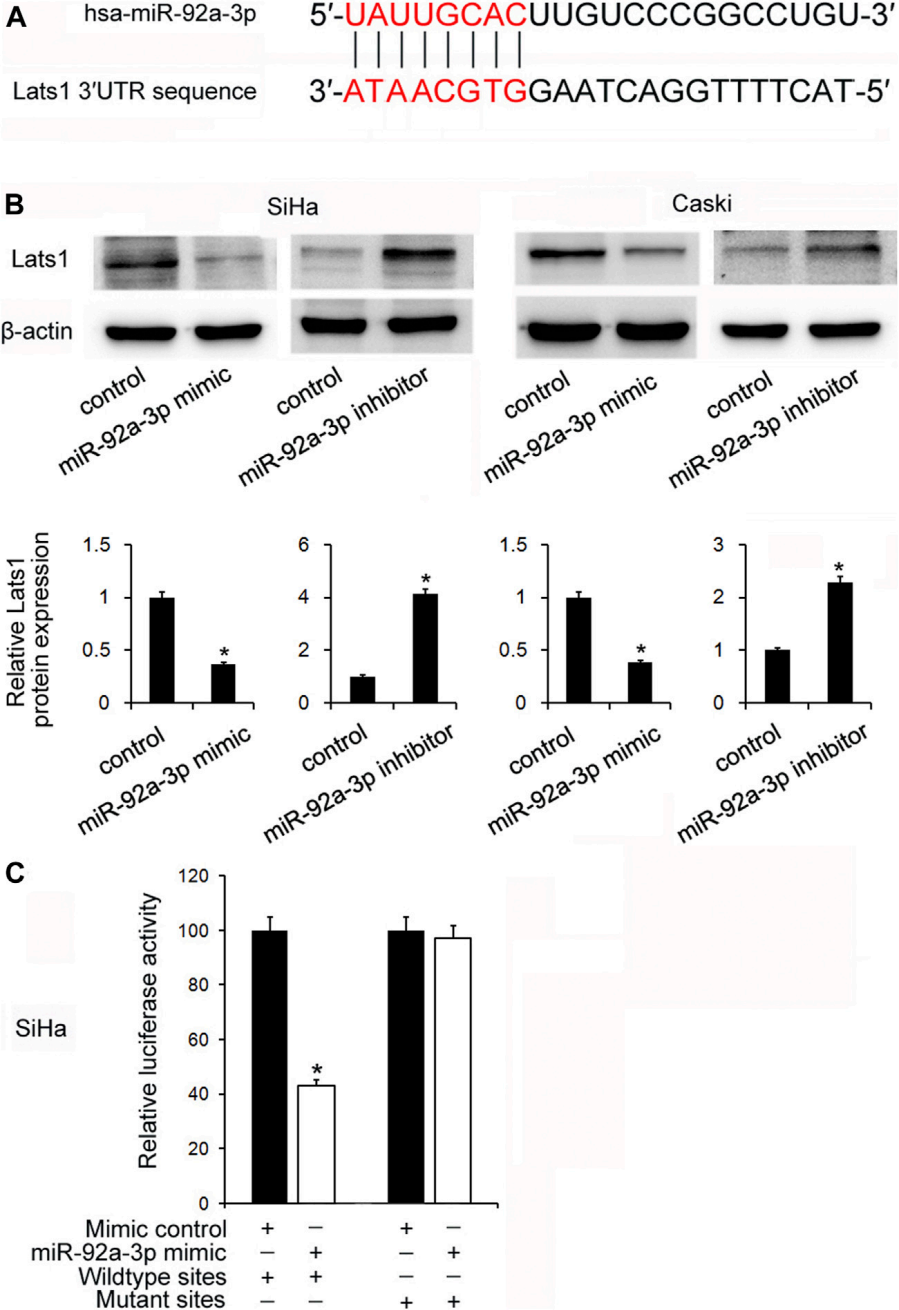
miR-92a-3p targeted large tumor suppressor 1(LATS1)in cervical cancer. **(A)** The potential binding site between miR-92a-3p and LATS1 3′-UTR. **(B)** The miR-92a-3p mimic/mimic control and miR-92a-3p inhibitor/inhibitor control were transfected into SiHa and Caski cells respectively. Western blotting showed that LATS1 was decreased after miR-92a-3p mimic transfection and increased after miR-92a-3p inhibitor transfection. **(C)** miR-92a-3p mimic/mimic control and wild type/mutant reporter plasmid were co-transfected into the SiHa cells. miR-92a-3p mimic downregulated luciferase activity in cells transfected with the LATS1-wild type reporter plasmid. *p < 0.05.

TarBase v7.0 software predicted that miR-92a-3p had potential binding sites in the 3′-UTR of LATS1 (Figure 3A). Their relationship was validated by Western Blotting. Figure 3B shows that the miR-92a-3p mimic downregulated LATS1 protein levels, but the miR-92a-3p inhibitor upregulated them. In addition, a dual-luciferase reporter assay was used to verify whether miR-92a-3p directly targeted LATS1. SiHa cells were co-transfected with the luciferase reporter plasmid containing wild type mutated binding sites of LATS1 3′-UTR and the miR-92a-3p mimic. The results showed that miR-92a-3p downregulated luciferase activities in the cells transfected with the LATS1-wild type reporter plasmid, but there were no significant changes in cells transfected with LATS1-mutant reporter plasmid (Figure 3C).

### LATS1 Inhibited Proliferation, Invasion, and Cell Cycle in CC Stem Cells

**FIGURE 4:**
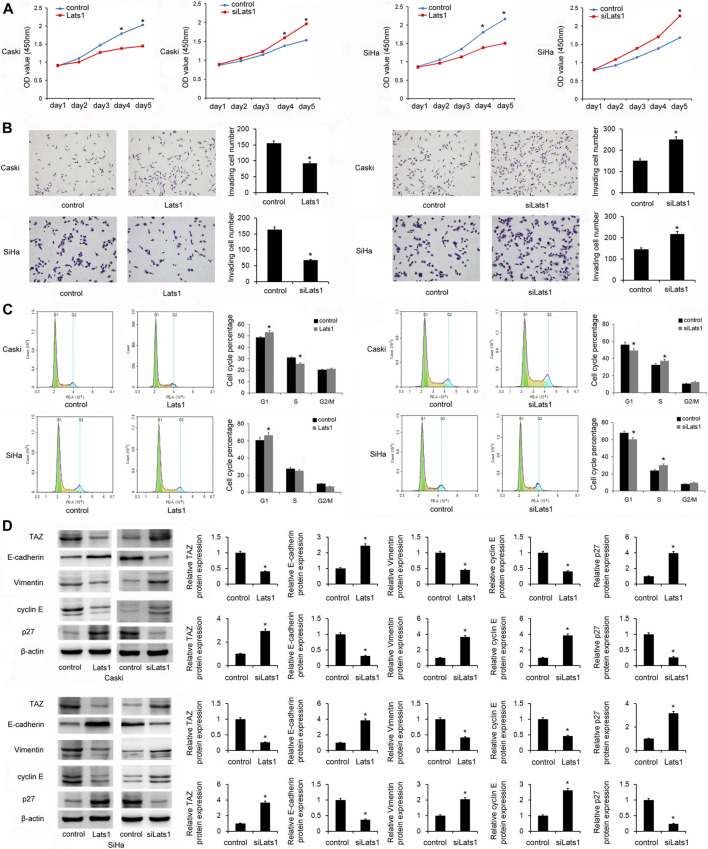
LATS1 inhibited proliferation, invasion, and the cell cycle in cervical cancer stem cells. **(A)** The Cell Counting Kit-8 assay showed that LATS1 overerxpression inhibited cell growth in SiHa and Caski cells. Large tumor suppressor 1 (LATS1) siRNA increased the cell growth in SiHa and Caski cells. **(B)** The Transwell invasion assay showed that LATS1 overexpression decreased invading cell numbers while LATS1 siRNA knockdown increased invading cell numbers **(C)** Flow cytometry showed that LATS1 overexpression increased the G1 phase percentage and decreased the S phase percentage. LATS1 siRNA decreased the G1 percentage and upregulated the S phase percentage. **(D)** Western blotting results showed that LATS1 overexpression downregulated TAZ, vimentin, cyclin E, and upregulated E-cadherin and p27 protein levels in SiHa and Caski cells. LATS1 silencing upregulated TAZ, vimentin, cyclin E, and downregulated E-cadherin and p27 levels in SiHa and Caski cells. *p < 0.05.

Then it was determined that whether LATS1 affected proliferation, invasion, and cell cycle of CC stem cells. The CCK8 assay showed that overexpression of LATS1 decreased cell growth in the SiHa and CaSki stem cells, and LATS1 siRNA knockdown increased the growth (Figure 4A). The Transwell invasion assay showed that LATS1 overexpression decreased invading cell numbers, and LATS1 siRNA knockdown increased the number (Figure 4B). Flow cytometry showed that overexpression of LATS1 increased the percentage of cells in G1 phase but decreased it in S phase, and LATS1 knockdown showed the opposite effect (Figure 4C).

### LATS1 Downregulated TAZ and Upregulated E-Cadherin in CC Cells

In order to identify the mechanism of CC cell invasion and proliferation regulated by LATS1, we screened several proteins which are potentially associated. Expressions of the epithelial-mesenchymal transition (EMT)-related factors, E-cadherin and vimentin were detected in the CaSki and SiHa cells transfected with the LATS1 plasmid and siRNA. Western Blotting showed that overexpression of LATS1 decreased the levels of TAZ, vimentin and cyclin E, but increased the protein levels of E-cadherin and p27 in SiHa and CaSki cells. LATS1 siRNA silencing increased the protein levels of TAZ, vimentin and cyclin E, but it decreased the protein levels of E-cadherin and p27 in SiHa and CaSki cells (Figure 4D).

### miR-92a-3p Regulated Proliferation and Invasion by Targeting LATS1 in CC Stem Cells

**FIGURE 5:**
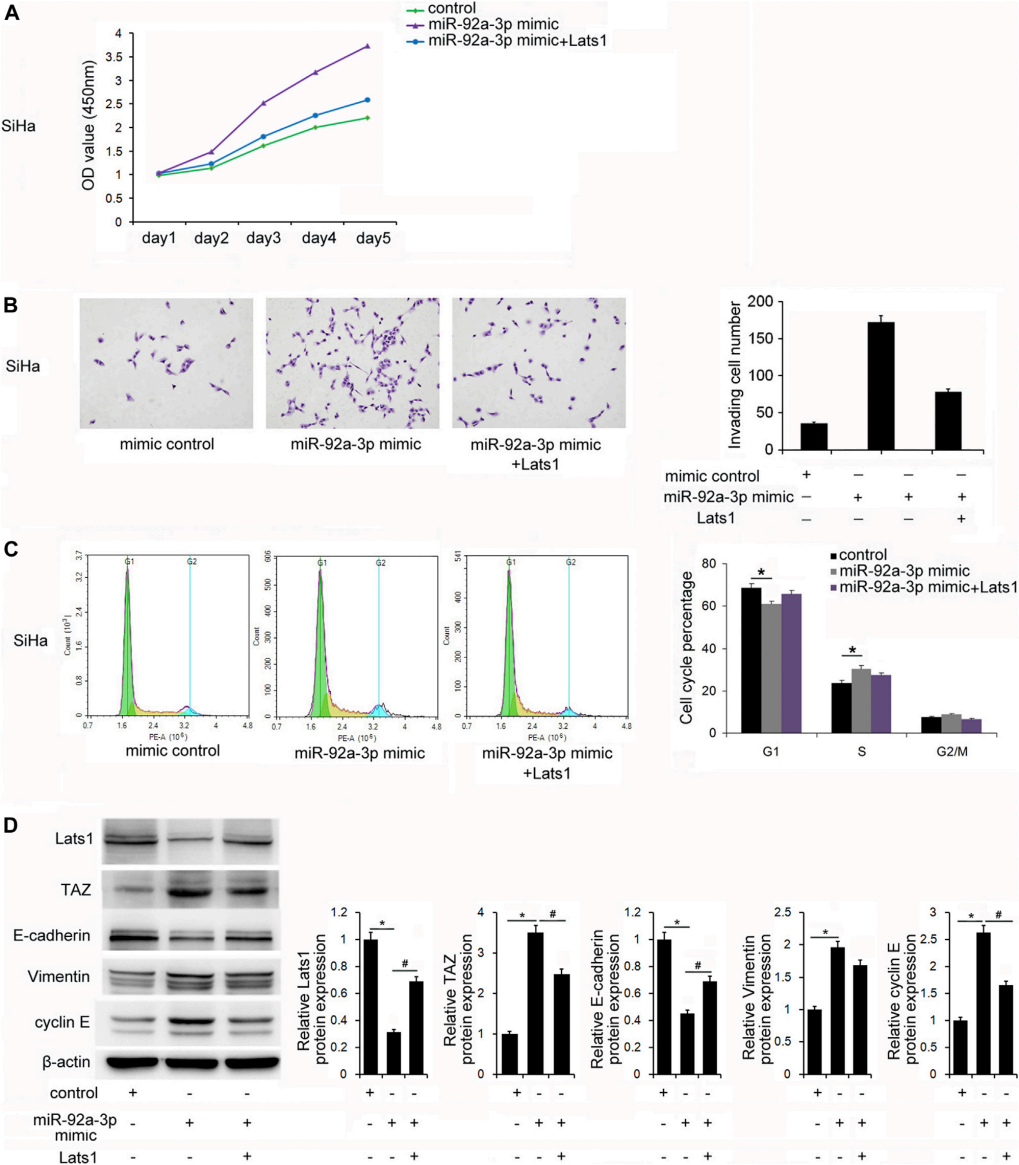
miR-92a-3p regulated biological behaviors *via* targeting large tumor suppressor 1 (LATS1) in cervical cancer stem cells. **(A)** The Cell Counting Kit-8 assay showed that the miR-92a-3p mimic increased cell growth, and restoration of LATS1 in cells with miR-92a-3p overexpression suppresssed growth. **(B)** The Transwell invasion assay results showed that the number of invading cells was increased after transfection of the miR-92a-3p mimic. LATS1 restoration abolished the positive effect of miR-92a-3p on invasion. **(C)** Flow cytometry results showed that miR-92a-3p overexpression significantly decreased the G1 percentage and increased the S percentage of cells. LATS1 transfection reversed the effect of miR-92a-3p on the G1-S cell cycle progression. **(D)** Western blotting results showed that miR-92a-3p overexpression downregulated LATS1, E-cadherin, and upregulated TAZ, vimentin, and cyclin E. LATS1 restoration downregulated TAZ, Vimentin, cyclin E and upregulated E-cadherin. *p < 0.05 miR-92a-3p mimic vs. the control; ^#^ *p <* 0.05 miR-92a-3p mimic + Lats1 vs. the miR-92a-3p mimic.

To further confirm whether miR-92a-3p regulated biological behaviors *via* targeting LATS1, SiHa cells were transfected with the miR-92a-3p mimics and the LATS1 plasmid. CCK8 assays showed that cell growth increased after transfection with the miR-92a-3p mimics, and restoration of LATS1 reversed the upregulation of growth (Figure 5A). The Transwell assay showed that the number of invading cells increased after transfection of the miR-92a-3p mimics, and LATS1 restoration abolished the positive effect of miR-92a-3p on invasion (Figure 5B). Similarly, LATS1 transfection reversed the effect of miR-92a-3p on G1-S cell cycle progression (Figure 5C). These results indicated that miR-92a-3p regulated the proliferation, invasion, and cell cycle of CC stem cells by targeting LATS1. Next, we examined if restoration of LATS1 reversed the effects of miR-92a-3p on TAZ, cyclinE and the EMT markers. Figure 5D shows that miR-92a-3p overexpression downregulated the protein levels of LATS1 and E-cadherin, but upregulated of TAZ, vimentin and cyclin E, and LATS1 plasmid transfection partly reversed the effects of miR-92a-3p on TAZ, E-cadherin, vimentin and cyclin E.

## Discussion

The miR-92a-3p is a member of miR-17-92 clusters that is located on chr13q31.3 within the third intron of the *C13orf25/MIR17HG* gene. miR-92a-3p was considered as a cancer-related microRNA. Recent evidences suggested that miR-92a-3p was upregulated in several cancers, including esophageal squamous cell cancer (Li et al., 2019), breast cancer (Cun and Yang, 2018), gastric cancer (Zhang et al., 2018) and colorectal cancer (Fu et al., 2018). miR-92a was also reported to act as an onco-miRNA in CC. A report showed that the miR-92a level in the serum of patients with cervical cancer was higher than that in healthy volunteers (Kong et al., 2017). Other reports revealed that miR-92a was significantly upregulated in CC tissues and cell lines. The overexpression of miR-92a contributed to the malignant proliferation and invasion of CC, and both FBXW7 and DKK3 could be the target genes of miR-92a (Zhou et al., 2015; Luo et al., 2017). In the present study, analysis of TCGA dataset showed higher miR-92a-3p expression in CC tissues were found compared with that in normal tissues. More importantly, high miR-92a-3p levels correlated with patients’ poor prognosis, suggesting that miR-92a-3p could be a malignant biomarker for CC patients.

Previous studies showed that miR-92a-3p had regulatory effects on cancer proliferation, invasion and chemosensitivity. miR-92a-3p promoted the proliferation and invasion of esophageal squamous cell cancer by targeting PTEN (Li et al., 2019). miR-92a-3p induced the proliferation of renal cell carcinoma by targeting FBXW7 (Zeng et al., 2020). Inhibition of miR-92a-3p induced apoptosis in colorectal cancer cells (Ahmadi et al., 2016). In this study, it was showed that miR-92a-3p promoted proliferation, invasion, and cell cycle transition in CC stem cells. The overexpression of miR-92a-3p downregulated LATS1, which was a potential tumor suppressor in CC stem cells. It was also found that there was a potential binding site between miR-92a-3p and the LATS1 3′-UTR, which was furtehr validated with the luciferase reporter assay. What’ more, it revealed that the role that miR-92a-3p mimics played on CC cell proliferation and invasion was partially blocked by LATS1, which indicated that LATS1 mediated the downstream oncogenic effects of miR-92a-3p.

LATS1 is a core component of the Hippo signaling pathway (Janse van Rensburg and Yang, 2018; Taha et al., 2018). The Hippo signaling exerts a critical role in modulating cell proliferation and has been demonstrated to contribute to the progression of various diseases, involving cancers. The Hippo signaling pathway is primarily composed of MST1/2, LATS1/2, and YAP/TAZ. Following activation of the Hippo pathway, MST1/2 is phosphorylated to activate LATS1/2, which can then phosphorylate YAP/TAZ, and resulting in the inhibition of activity of YAP/TAZ (Harvey et al., 2013; Han, 2019). In this study, we found that LATS1 levels, rather than the LATS kinases activity, affected the CC progression. The overexpression of LATS1 inhibited the proliferation of CC stem cells, but LATS1 depletion showed the opposite effect. LATS1 silencing promoted the G1-S transition in CC stem cells, and the overexpression of LATS1 inhibited. Accelerated cell cycle progression is one of the hallmarks for malignant cancers caused by. Cyclins are orchestrators of the cell cycle, and their expression and activities fluctuate across specific phases. Cyclin E is required for the G1/S transition (Kisielewska et al., 2009). The p27 interacts with cyclin E binary complexes to inhibit their kinase activities, which negatively regulates cell cycle transition (Cheng et al., 2017). LATS1 has been reported to inhibit cyclin E/CDK2 activity and G1-S progression (Pefani et al., 2014). In this study, we found that the overexpression of LATS1 downregulated cyclin E but upregulated p27 expressions. The miR-92a-3p mimics upregulated cyclin E protein, which could be reversed by restoration of LATS1. These results suggested that the overexpression of miR-92a-3p might have induced the cell cycle of CC stem cells by the downregulation of LATS1, and which induced the upregulation of cyclin E.

Invasion and metastasis are the major causes of mortalites in patients with CC. The results here showed that LATS1 played a negative regulatory role during the invasion of CC stem cells. Changes of cancer invading abilities were closely related with the EMT, which was a cellular transition process from epithelial cells to an invasive mesenchymal-like phenotype (Qureshi et al., 2015). The EMT, therefore, plays an important role in malignant progression of CC. Acquisition of mesenchymal markers such as vimentin and loss of epithelial markers such as E-cadherin are the hallmarks of the EMT. Our results showed that LATS1 negatively regulated the proteins levels of vimentin and positively regulated E-cadherin protein in CC stem cells. The miR-92a-3p downregulated E-cadherin and upregulated vimentin, which was reversed by transfection of the LATS1 plasmid. These results are consistent with a previous report showing miR-92a-3p facilitated the EMT in endothelial cells (Yamada et al., 2019).

Regulation of the EMT by LATS1 has been previously reported (Wu et al., 2018). TAZ, a key downstream effector of LATS1, has also been revealed to induce the EMT (Janse van Rensburg and Yang, 2016). The results in this study confirmed that LATS1 downregulated TAZ in CC stem cells. All of the results suggested that the overexpression of miR-92a-3p promoted the TAZ-induced EMT of CC stem cells by downregulating LATS1, which might further have contributed to the increased cell invasion.

In conclusion, the present study showed that the overexpression of miR-92a-3p promoted proliferation, invasion and cell cycle progression in CC stem cells. Particularly, miR-92a-3p promoted proliferation of CC stem cells *via* regulating the protein levels of LATS1/cyclinE. And it promoted invasion by regulating the LATS1/TAZ/EMT signaling. Therefore, our findings provide insight into the novel role and mechanism of miR-92a-3p in CC stem cells.

## Data Availability

The raw data supporting the conclusions of this article will be made available by the authors, without undue reservation.
