# Supplementary figures and images for: miR-92a-3p Promoted EMT via Targeting LATS1 in Cervical Cancer Stem Cells
Source: Front Cell Dev Biol. 2021 Nov 18;9:757747. doi: 10.3389/fcell.2021.757747 (PMC8639224; doi:10.3389/fcell.2021.757747)

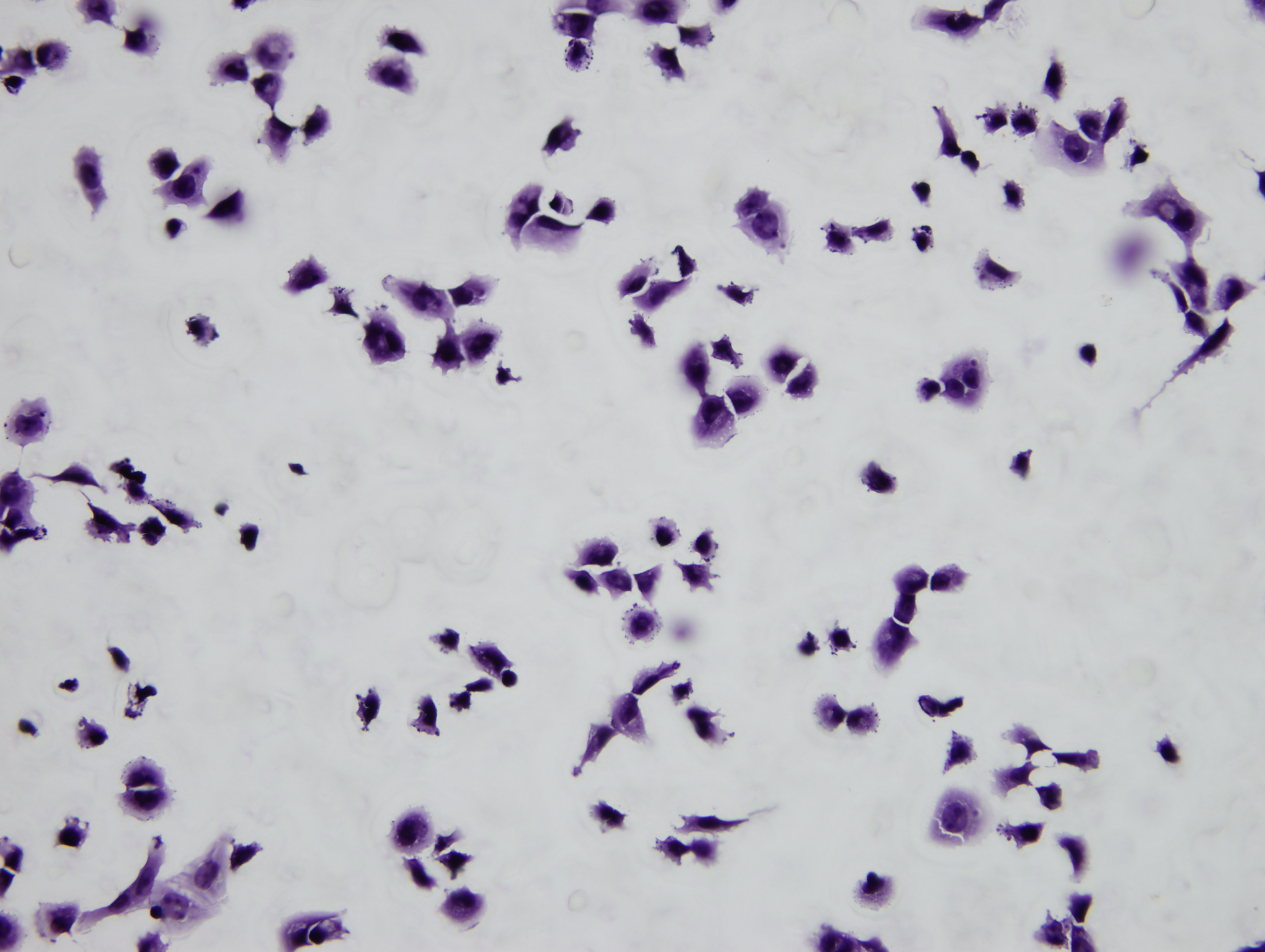

Supplement: Supplementary file 3 [file DataSheet3.ZIP › F2B right up control.tif]

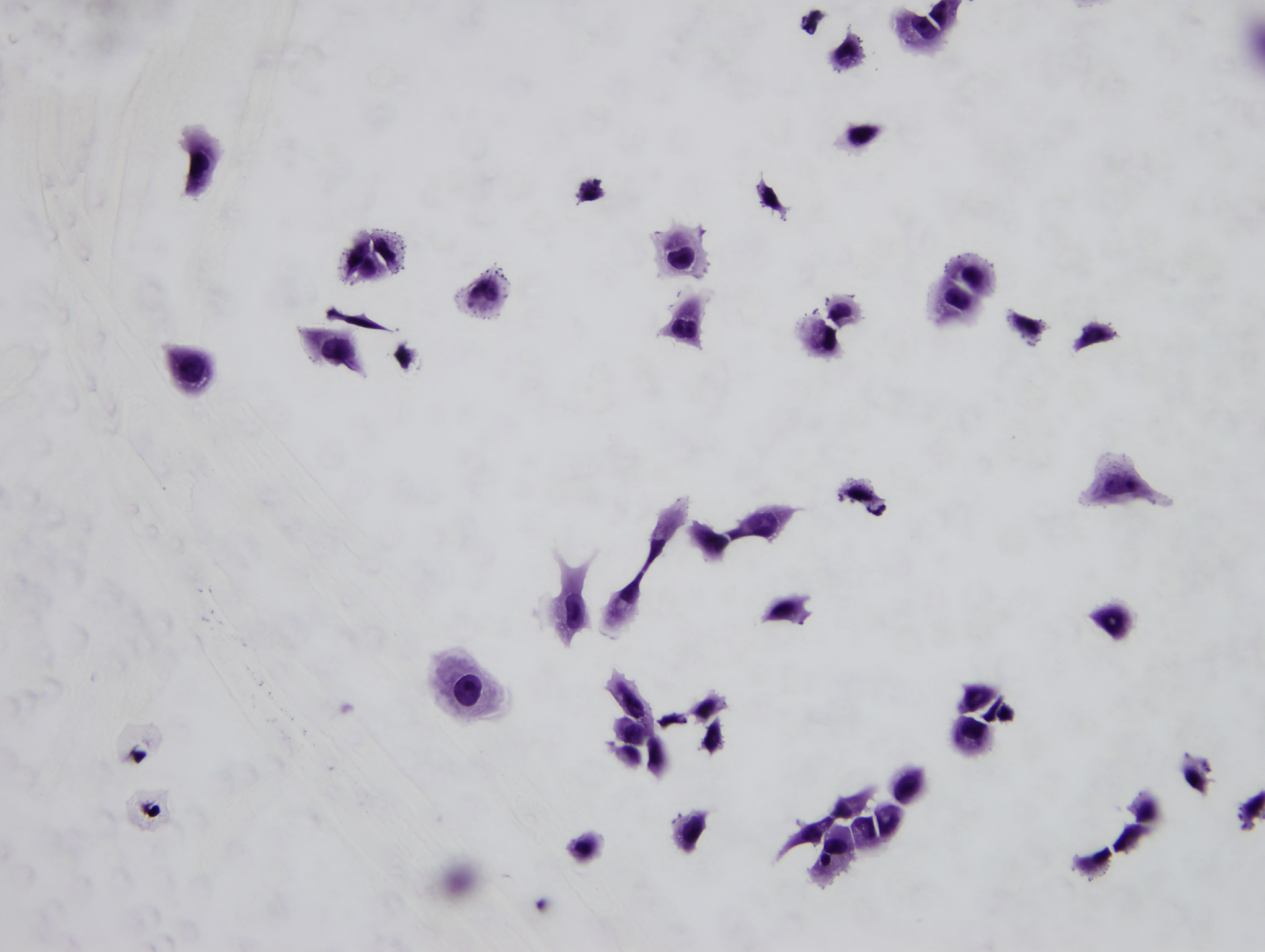

Supplement: Supplementary file 3 [file DataSheet3.ZIP › F2B right up miR-92a-3p inhibitor.tif]

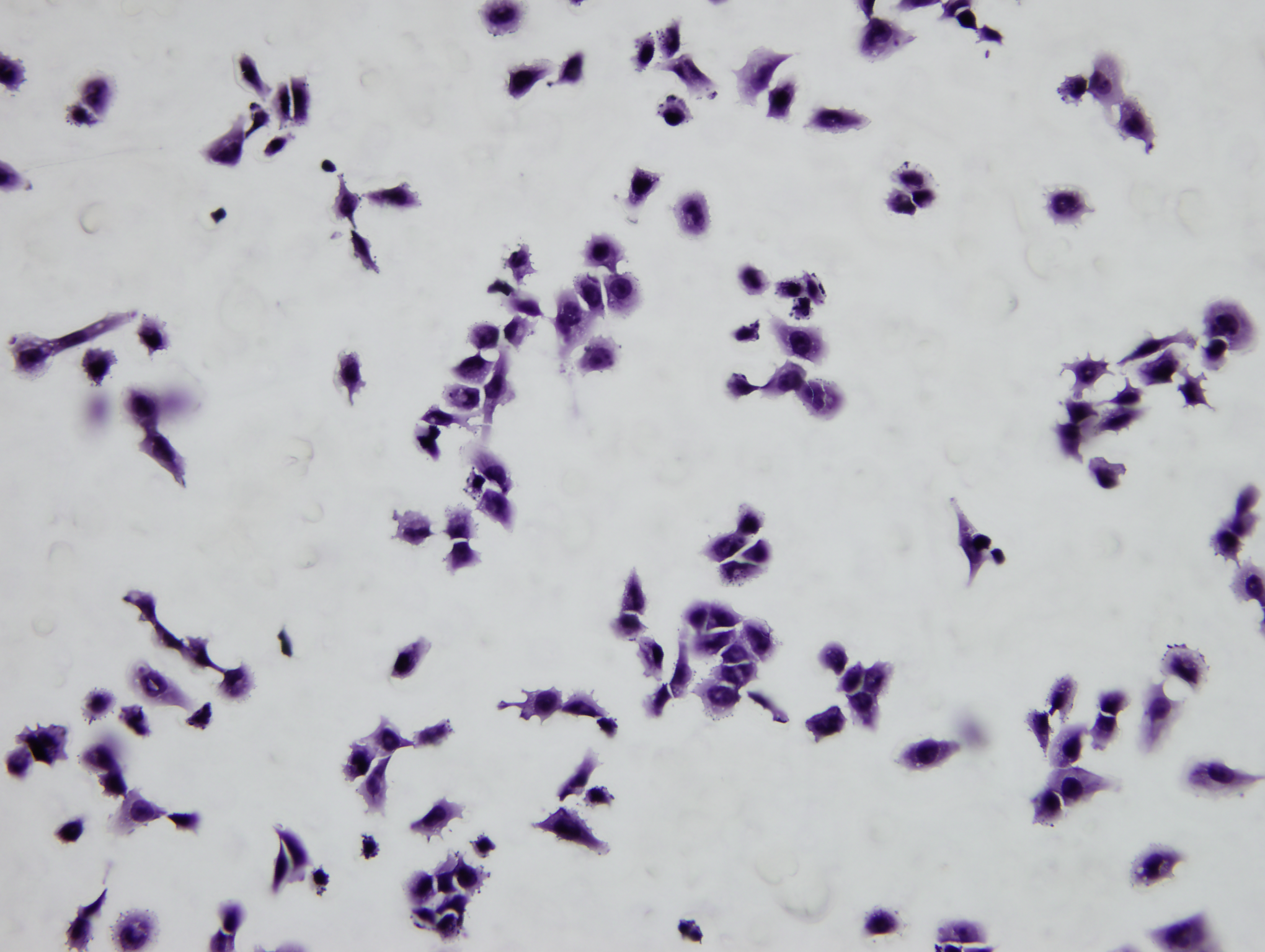

Supplement: Supplementary file 3 [file DataSheet3.ZIP › F4B left down control.tif]

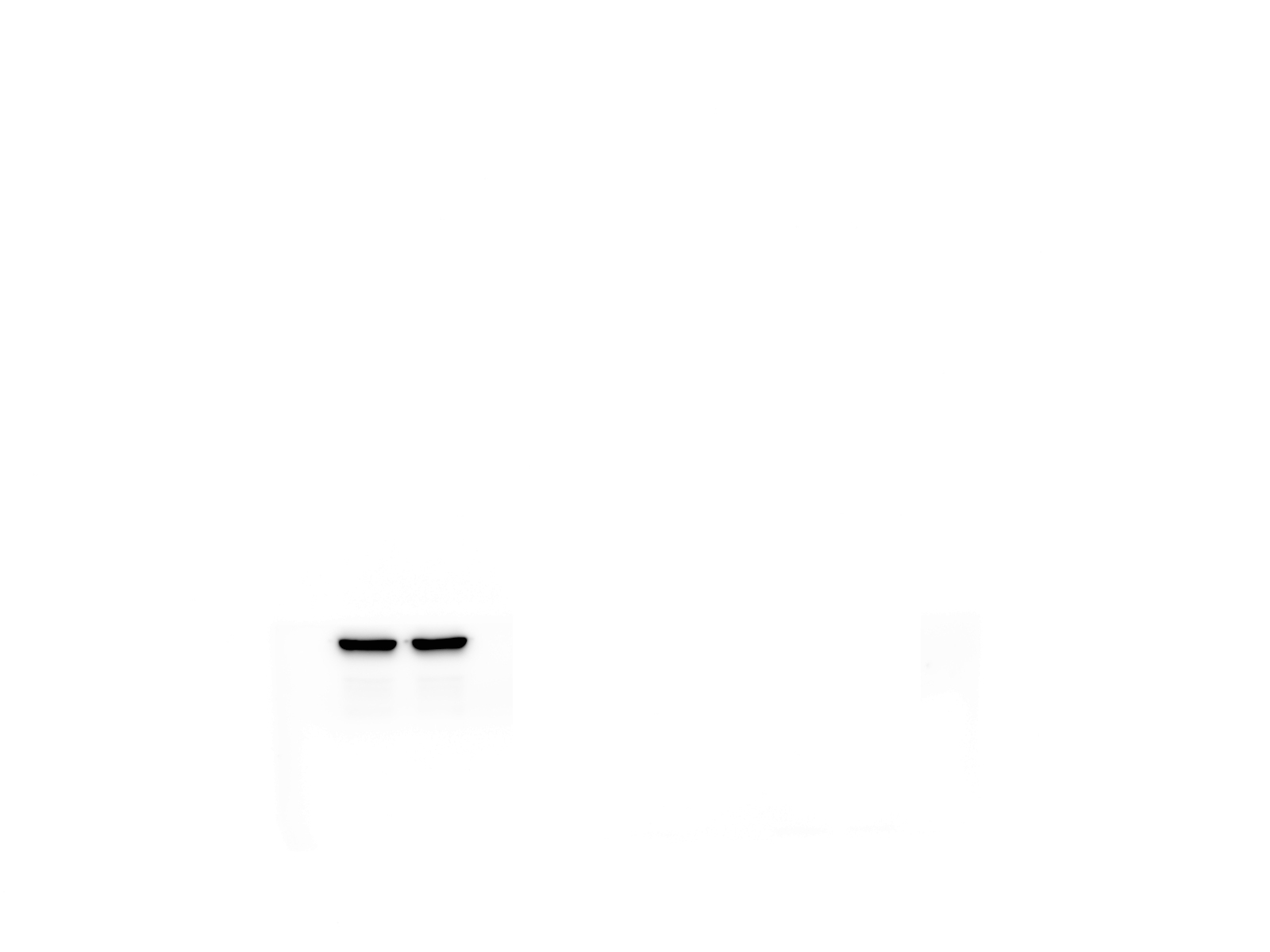

Supplement: Supplementary file 4 [file DataSheet8.ZIP › F3B left 1 actin.jpg]

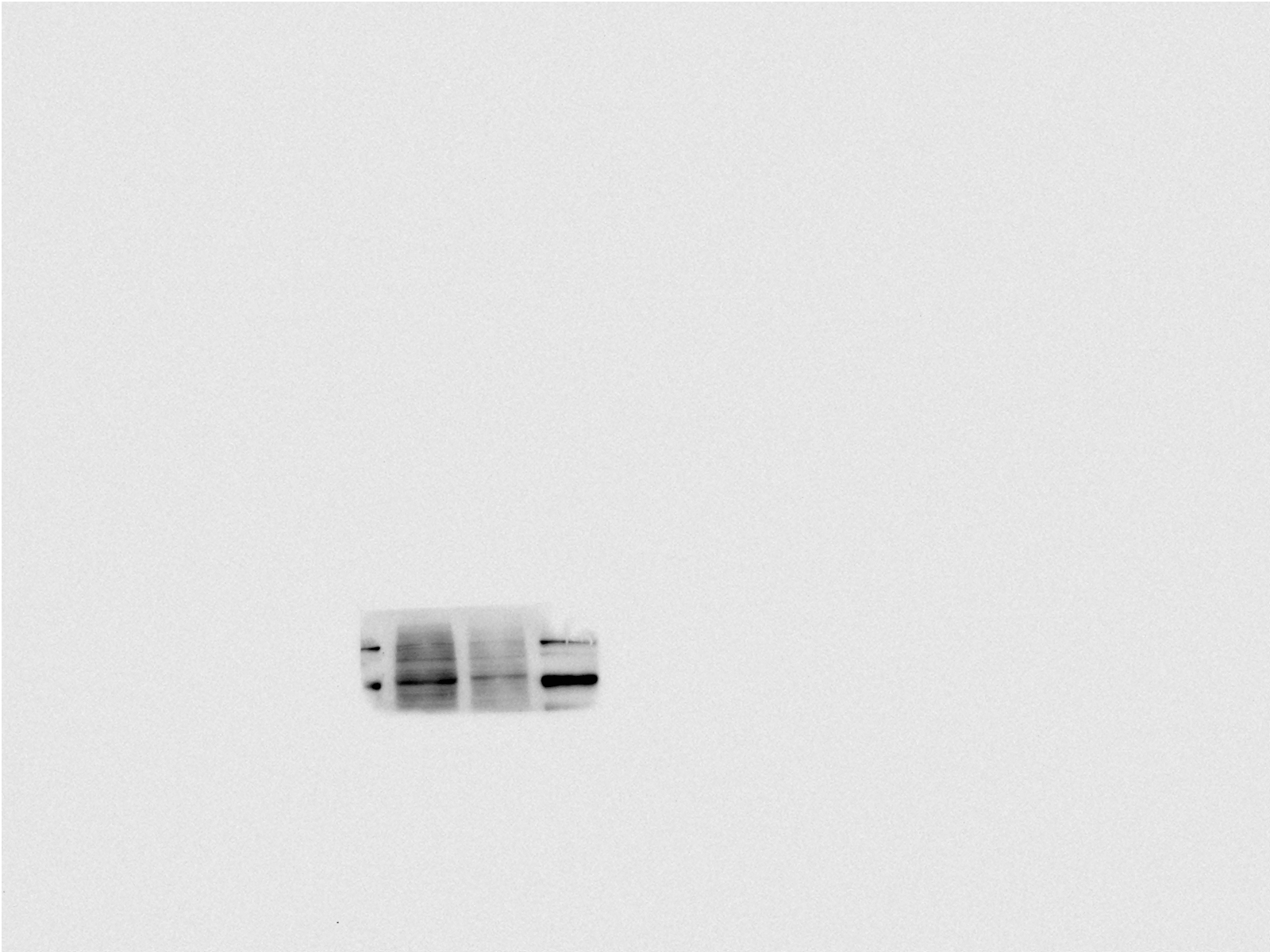

Supplement: Supplementary file 4 [file DataSheet8.ZIP › F3B left 1 lats1 .jpg]

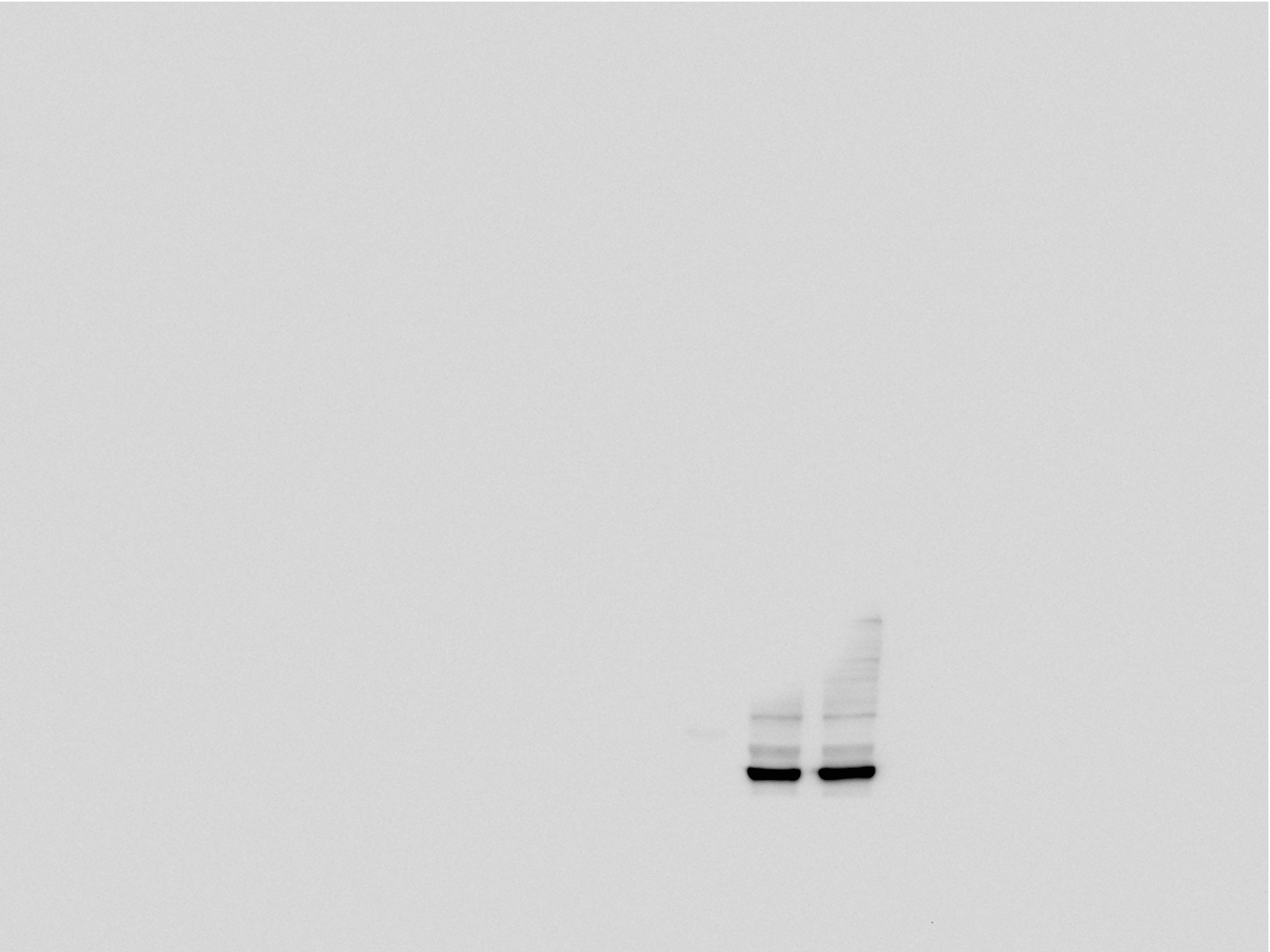

Supplement: Supplementary file 4 [file DataSheet8.ZIP › F3B left 2 actin.jpg]

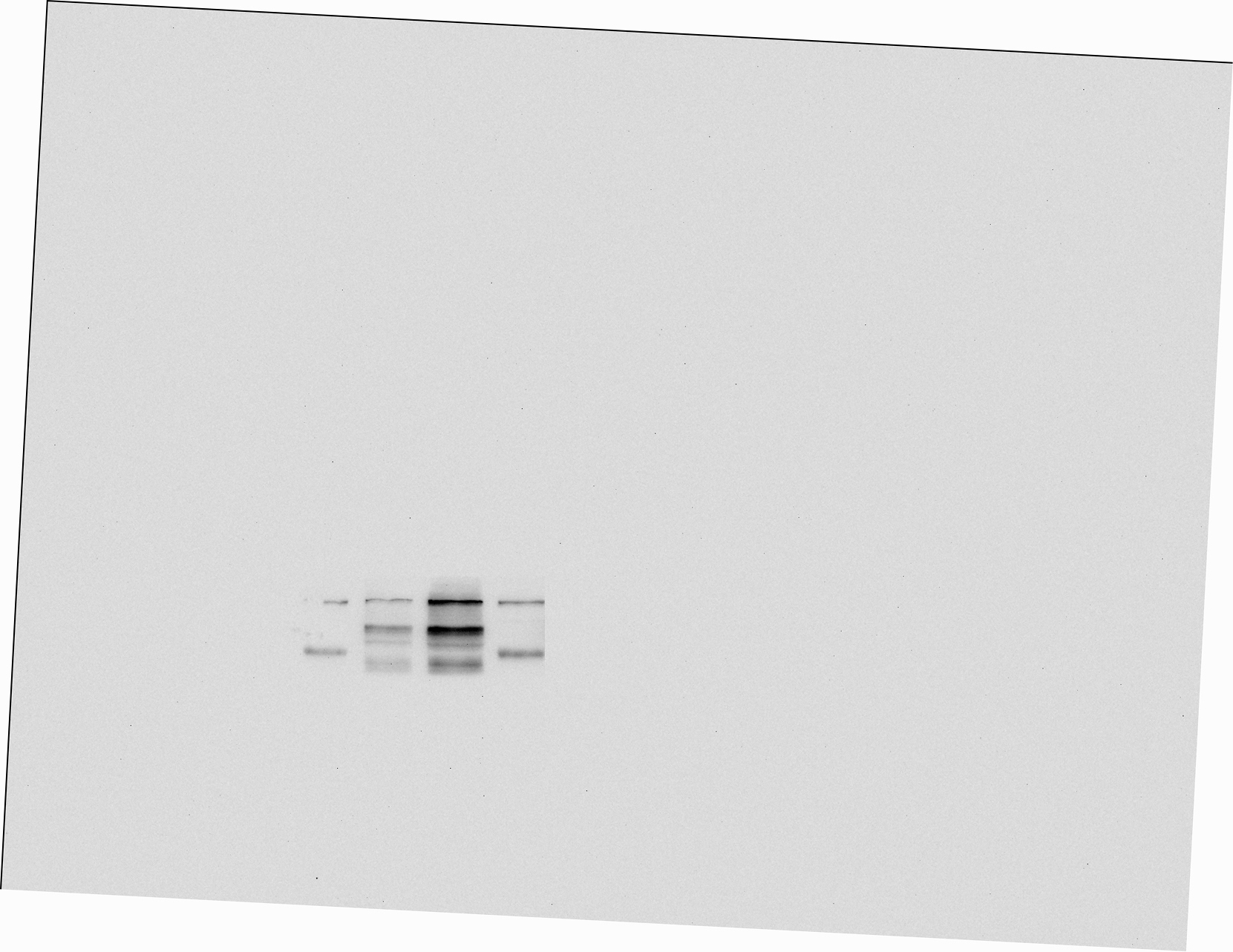

Supplement: Supplementary file 4 [file DataSheet8.ZIP › F3B left 2 lats1 .jpg]

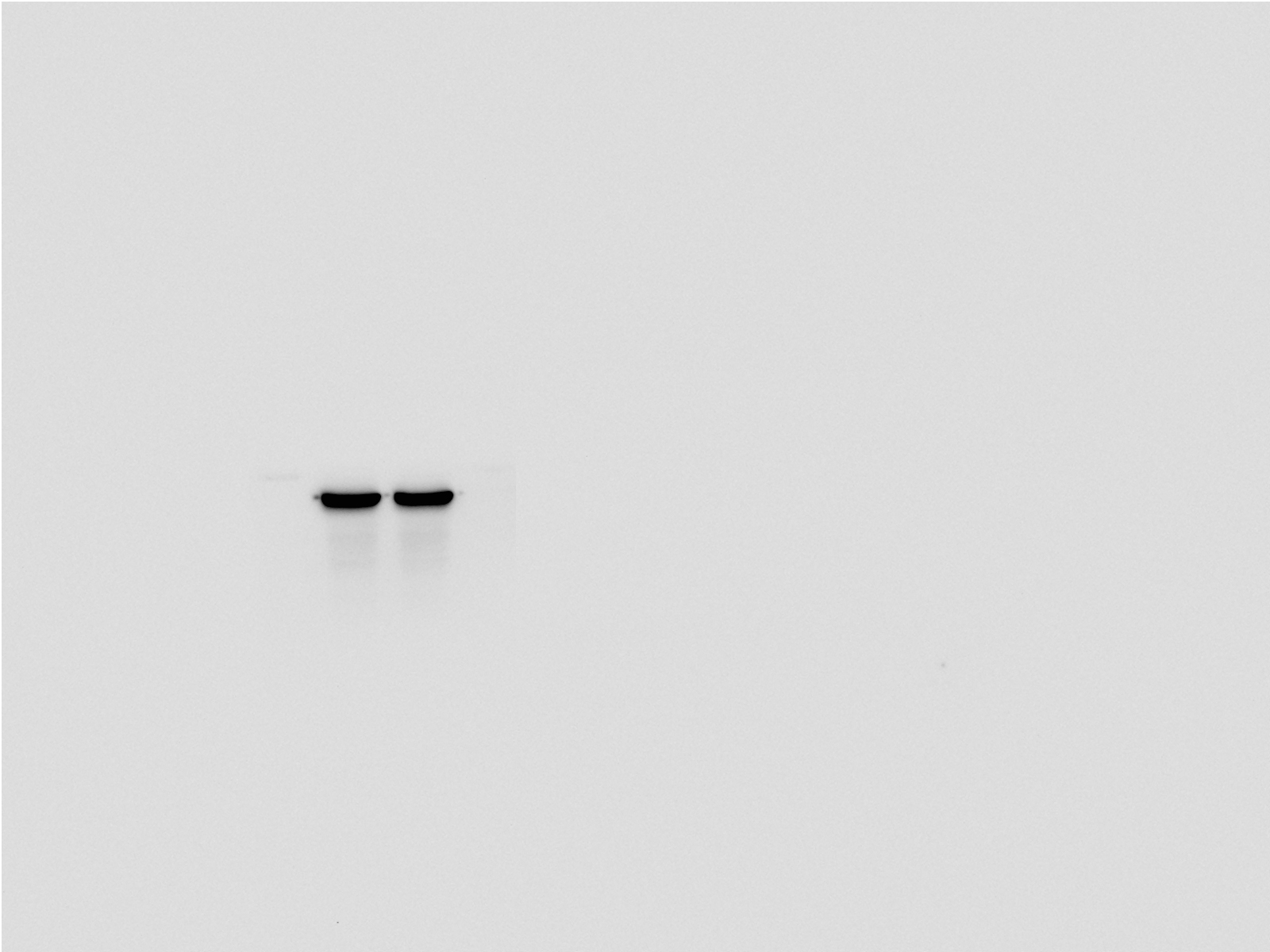

Supplement: Supplementary file 4 [file DataSheet8.ZIP › F3B left 3 actin.jpg]

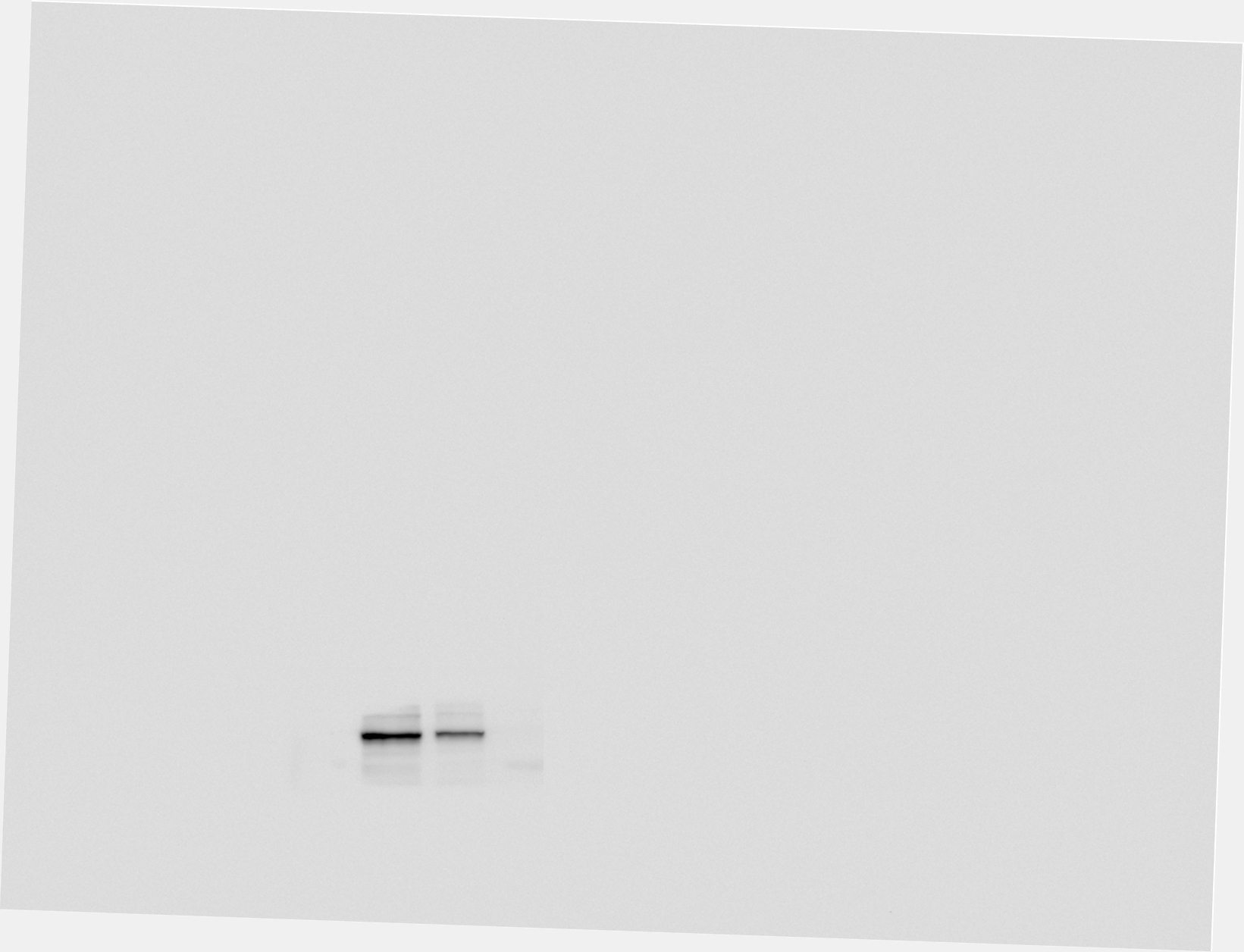

Supplement: Supplementary file 4 [file DataSheet8.ZIP › F3B left 3 lats1.jpg]

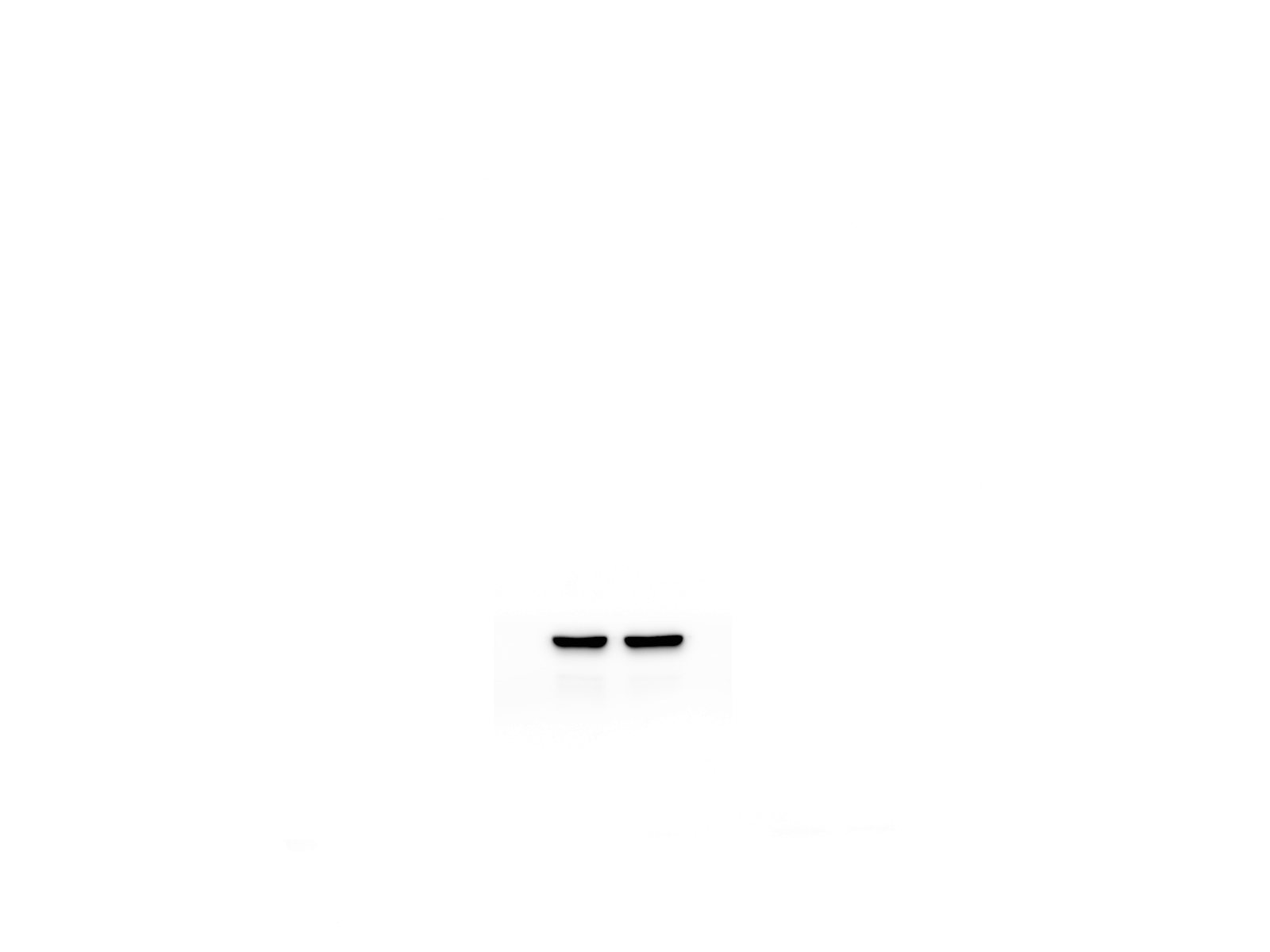

Supplement: Supplementary file 4 [file DataSheet8.ZIP › F3B left 4 actin.jpg]

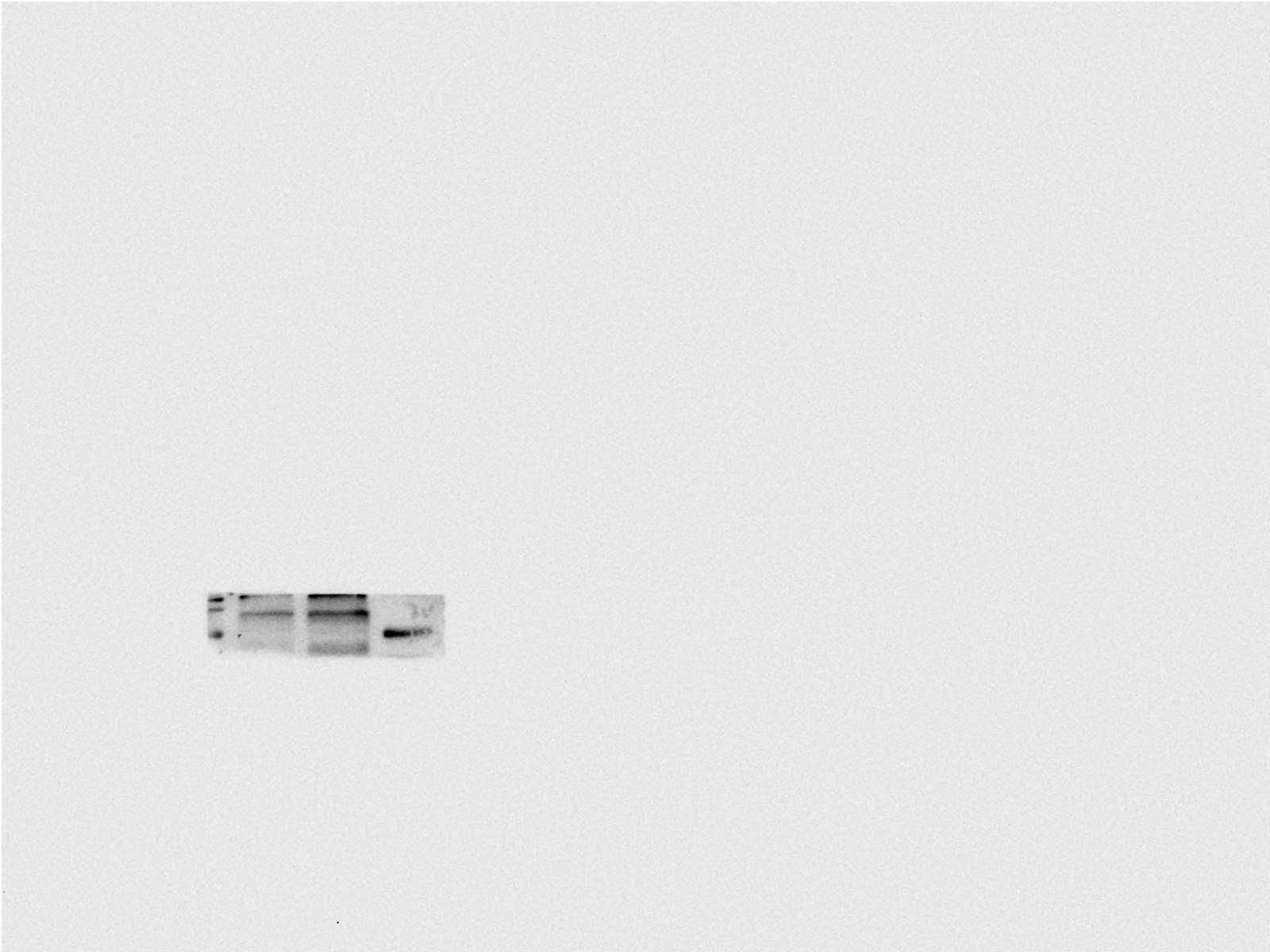

Supplement: Supplementary file 4 [file DataSheet8.ZIP › F3B left 4 lats1.jpg]

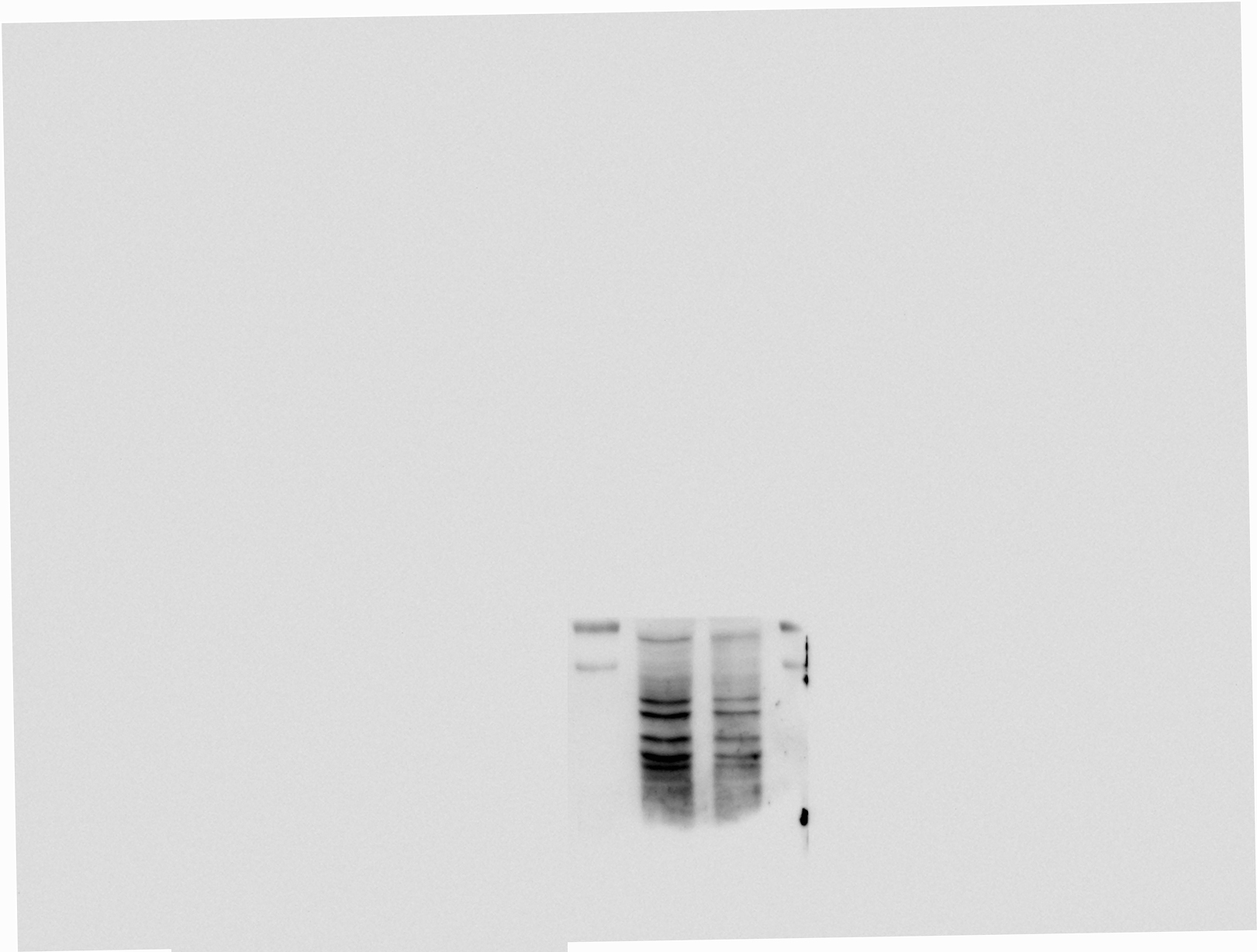

Supplement: Supplementary file 4 [file DataSheet8.ZIP › F4D left down cyclin E.jpg]

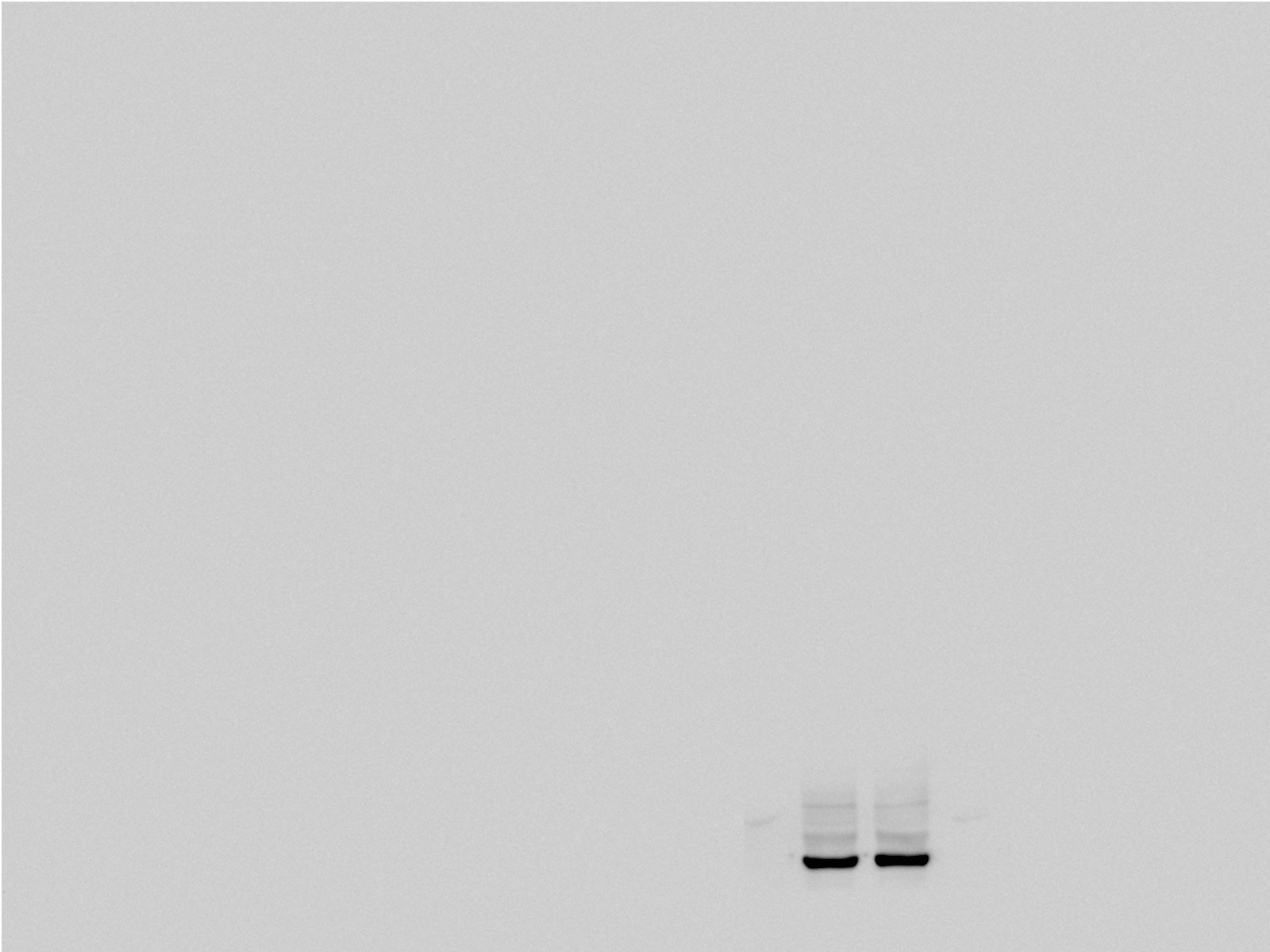

Supplement: Supplementary file 4 [file DataSheet8.ZIP › F4D left down actin.jpg]

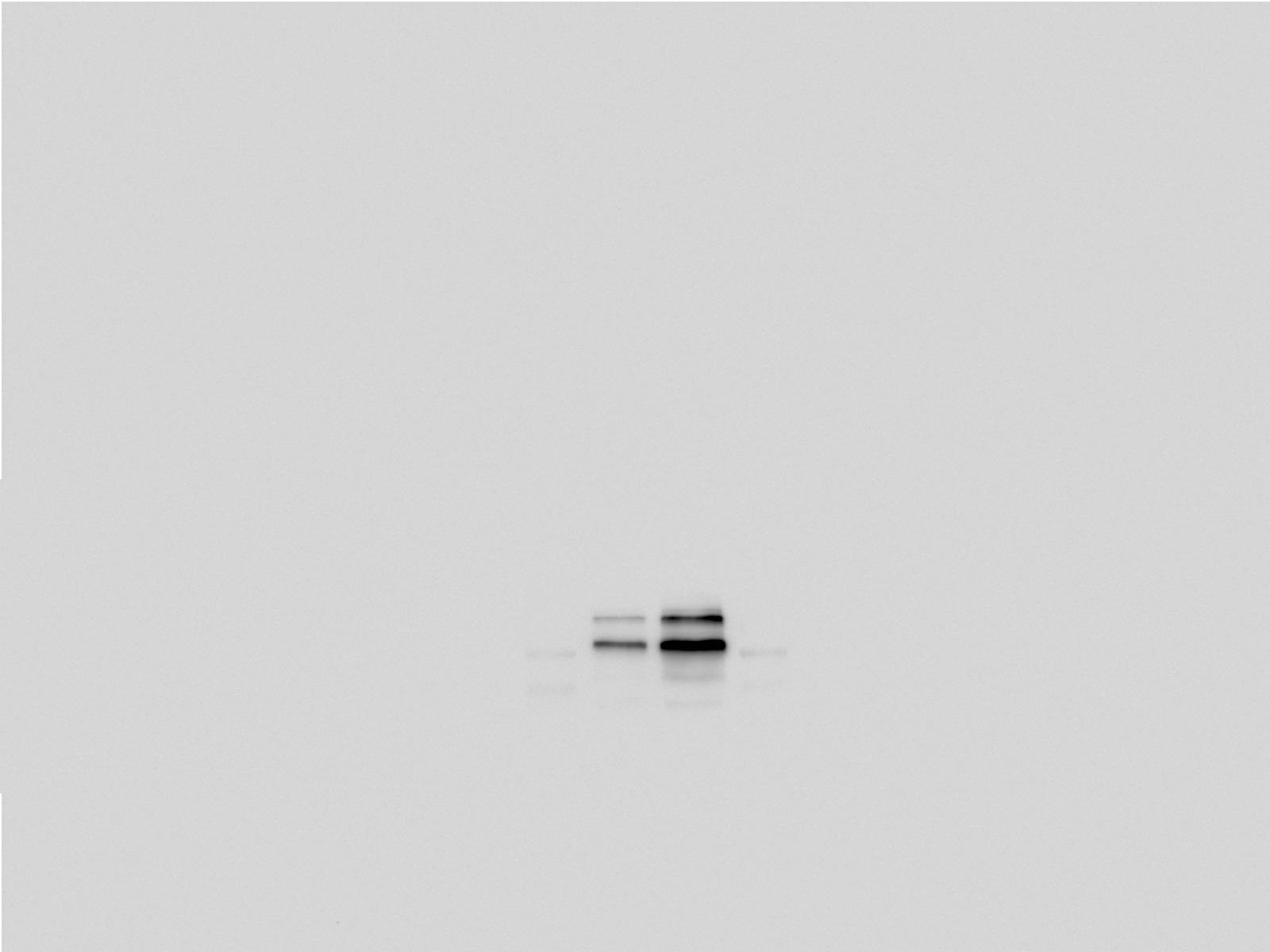

Supplement: Supplementary file 4 [file DataSheet8.ZIP › F4D left down E-Cadherin.jpg]

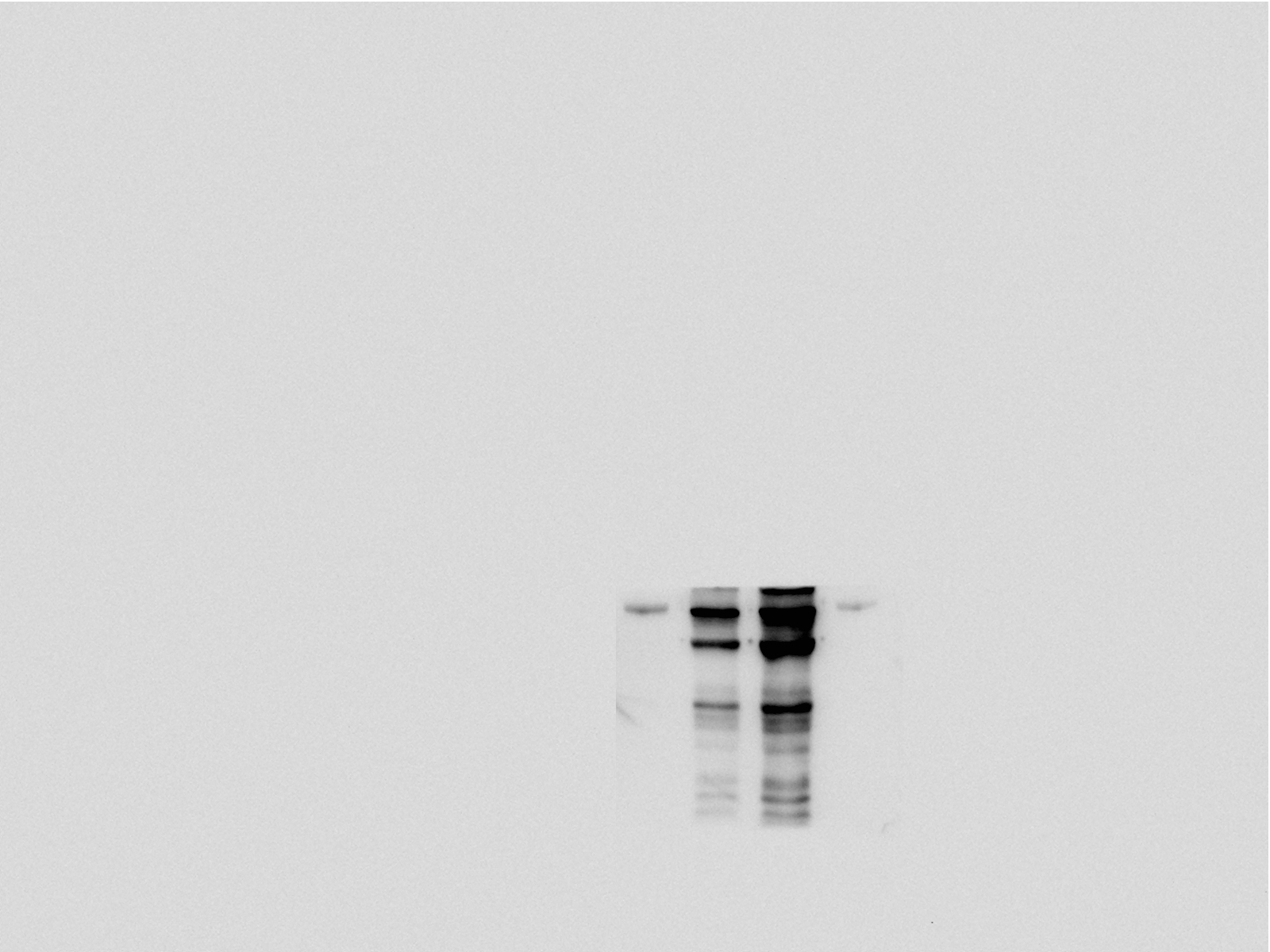

Supplement: Supplementary file 4 [file DataSheet8.ZIP › F4D left down p27.jpg]

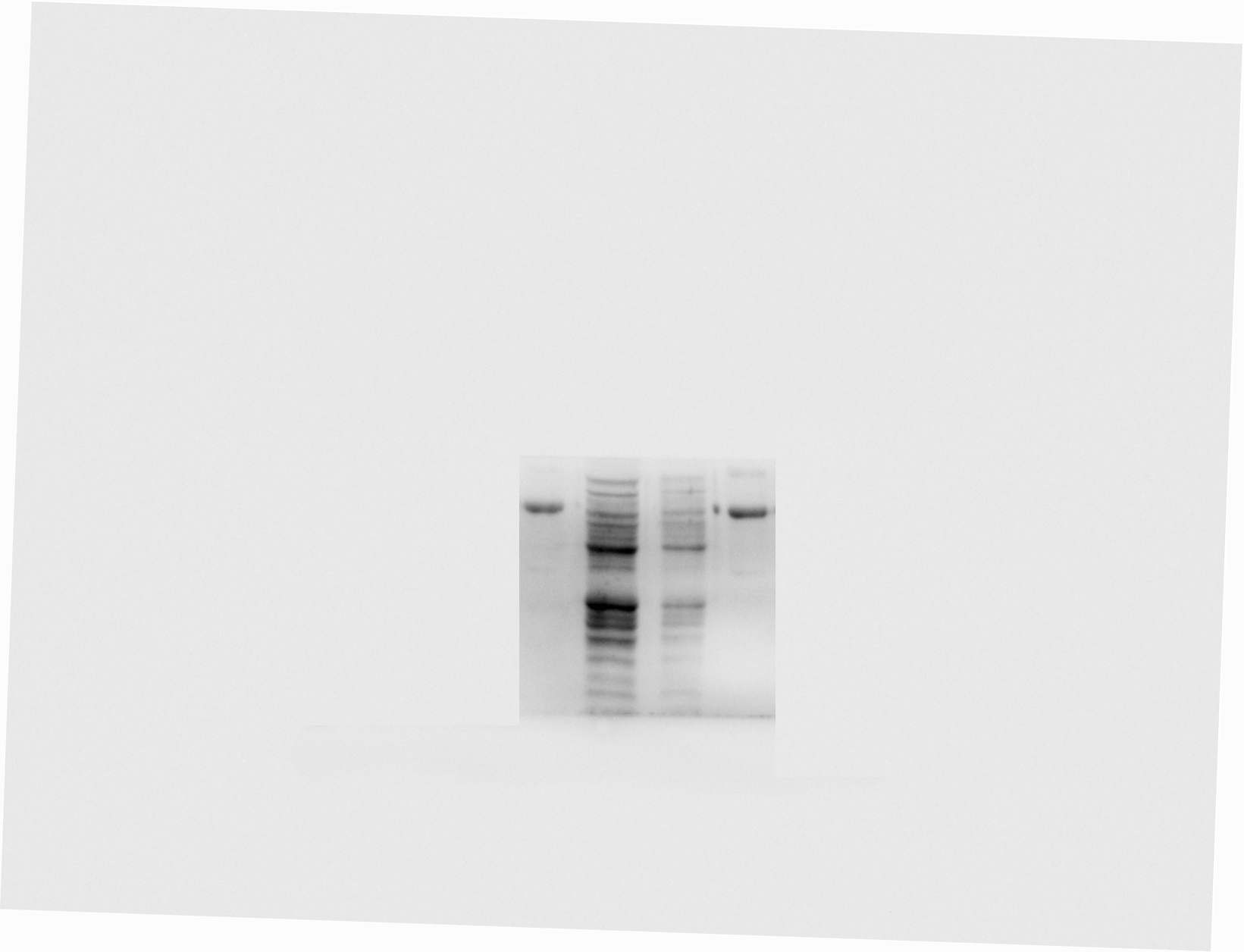

Supplement: Supplementary file 4 [file DataSheet8.ZIP › F4D left down TAZ.jpg]

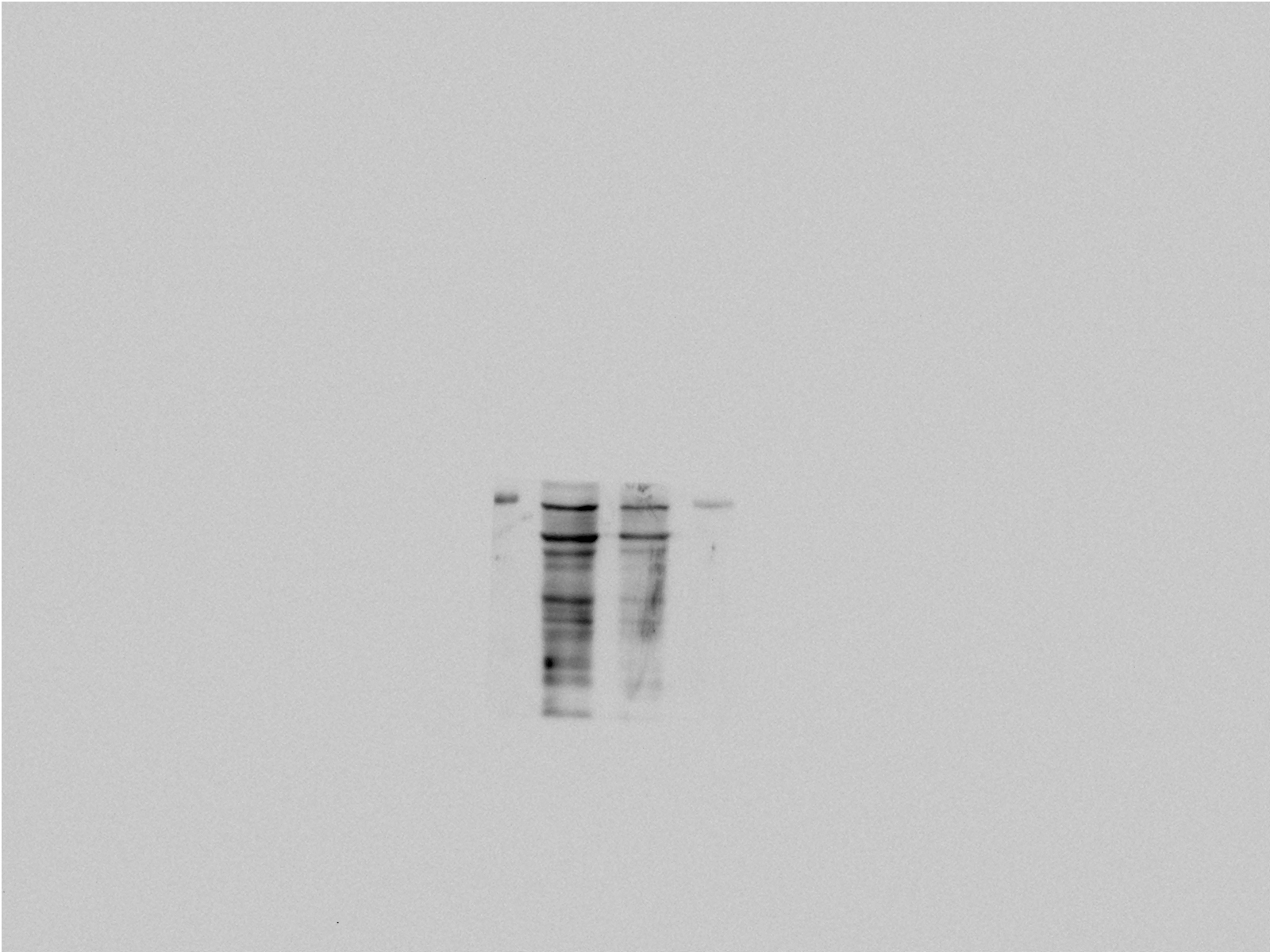

Supplement: Supplementary file 4 [file DataSheet8.ZIP › F4D left down vimentin.jpg]

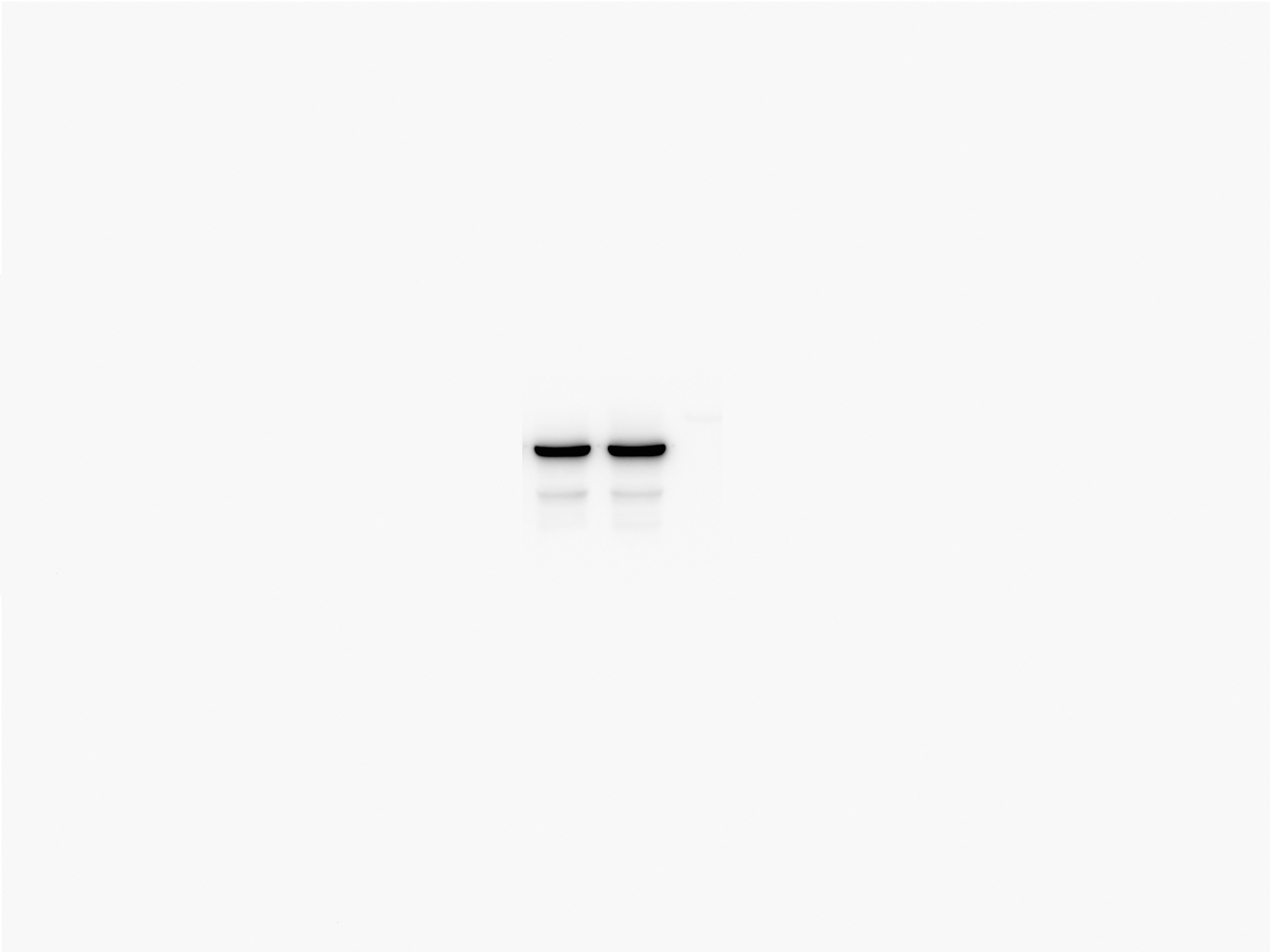

Supplement: Supplementary file 4 [file DataSheet8.ZIP › F4D left up actin.jpg]

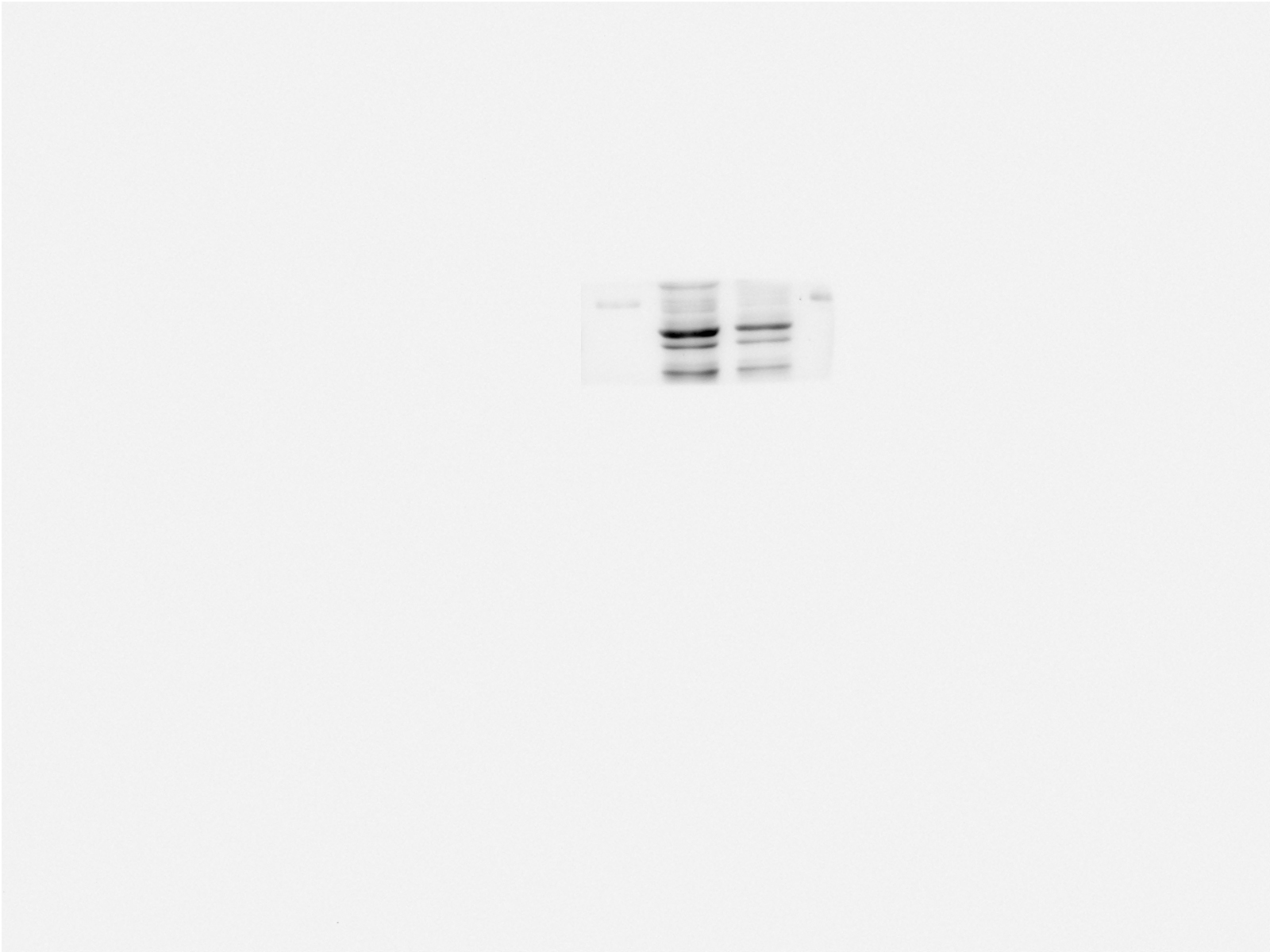

Supplement: Supplementary file 4 [file DataSheet8.ZIP › F4D left up cyclin E.jpg]

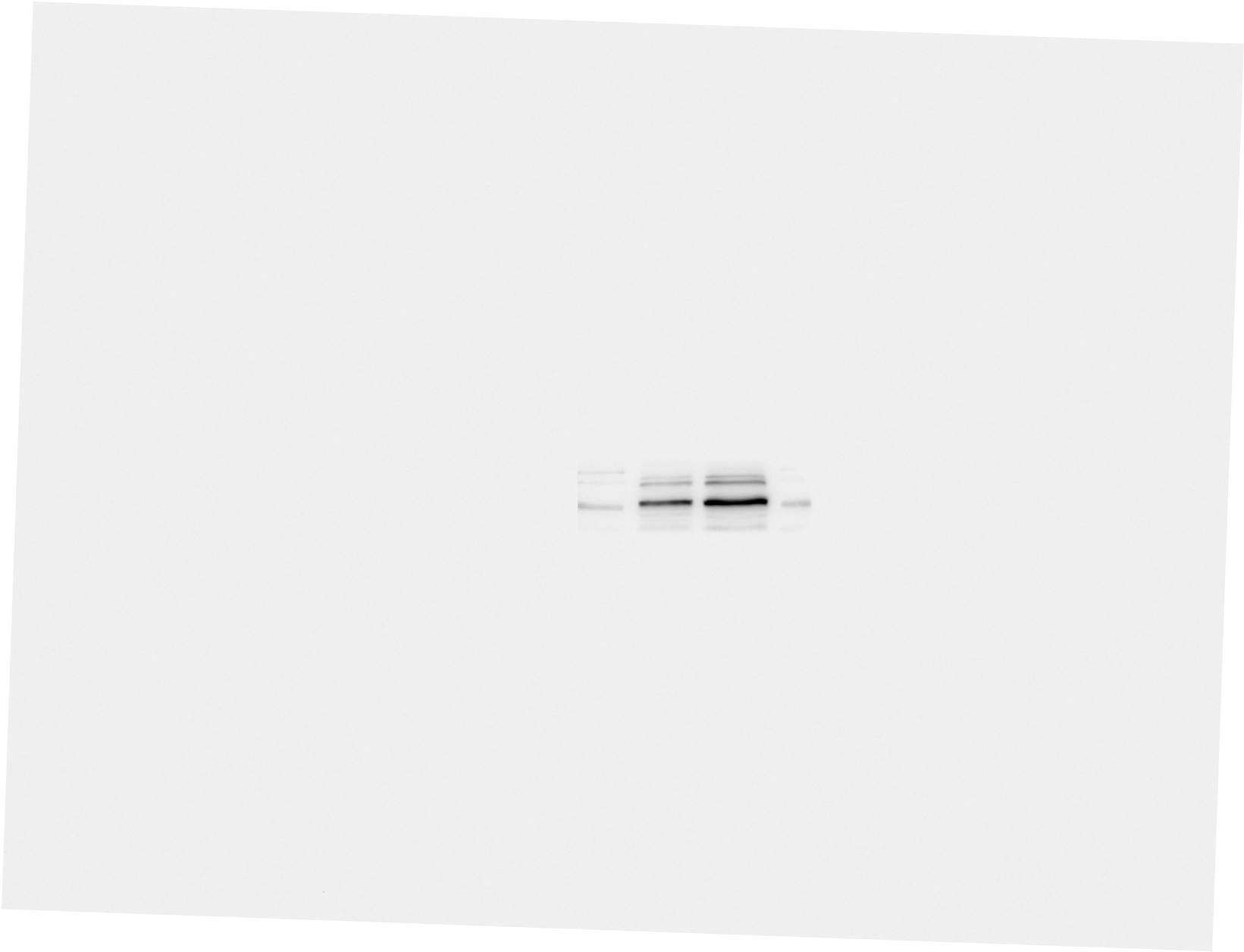

Supplement: Supplementary file 4 [file DataSheet8.ZIP › F4D left up E-Cadherin.jpg]

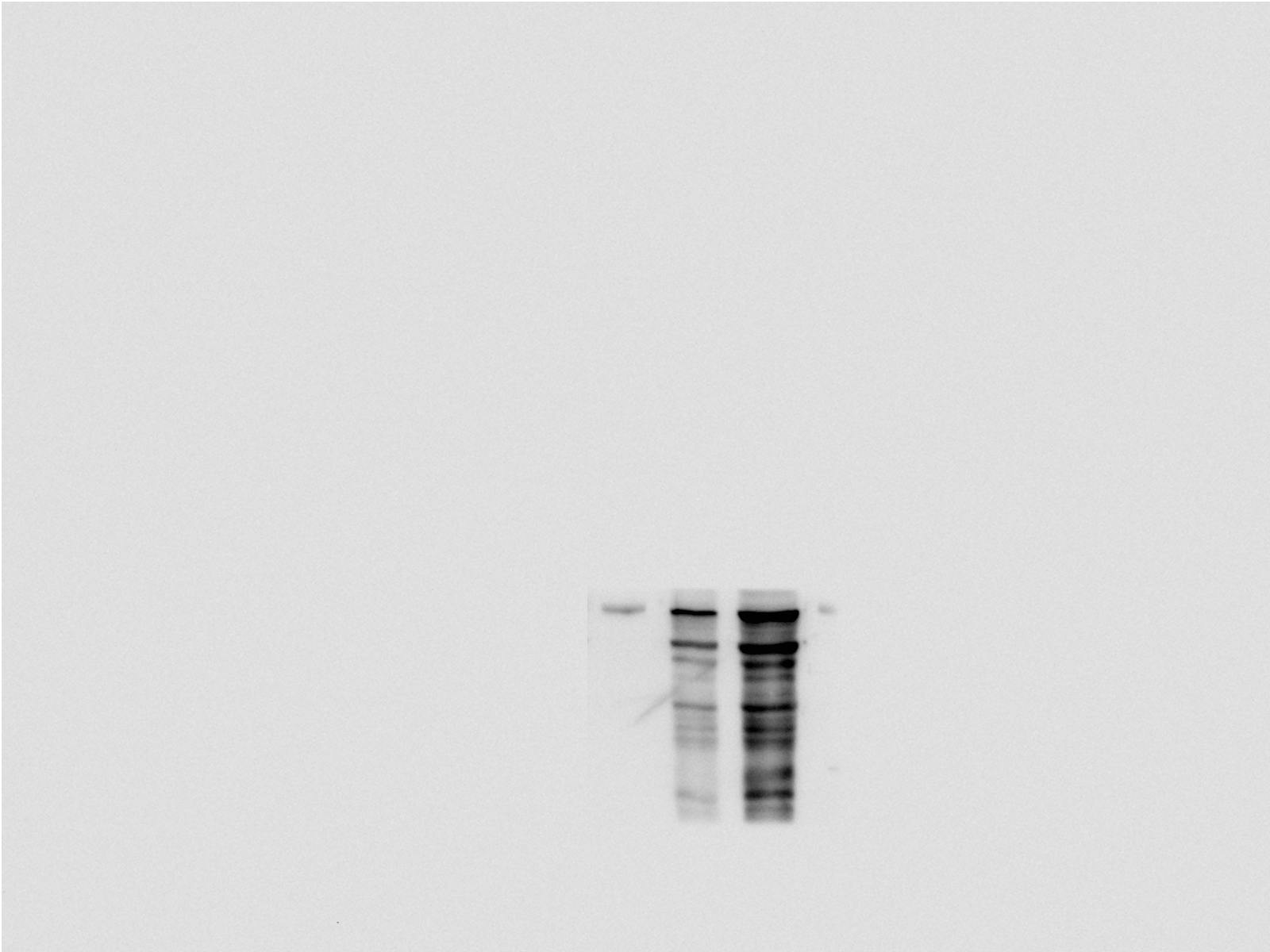

Supplement: Supplementary file 4 [file DataSheet8.ZIP › F4D left up p27.jpg]

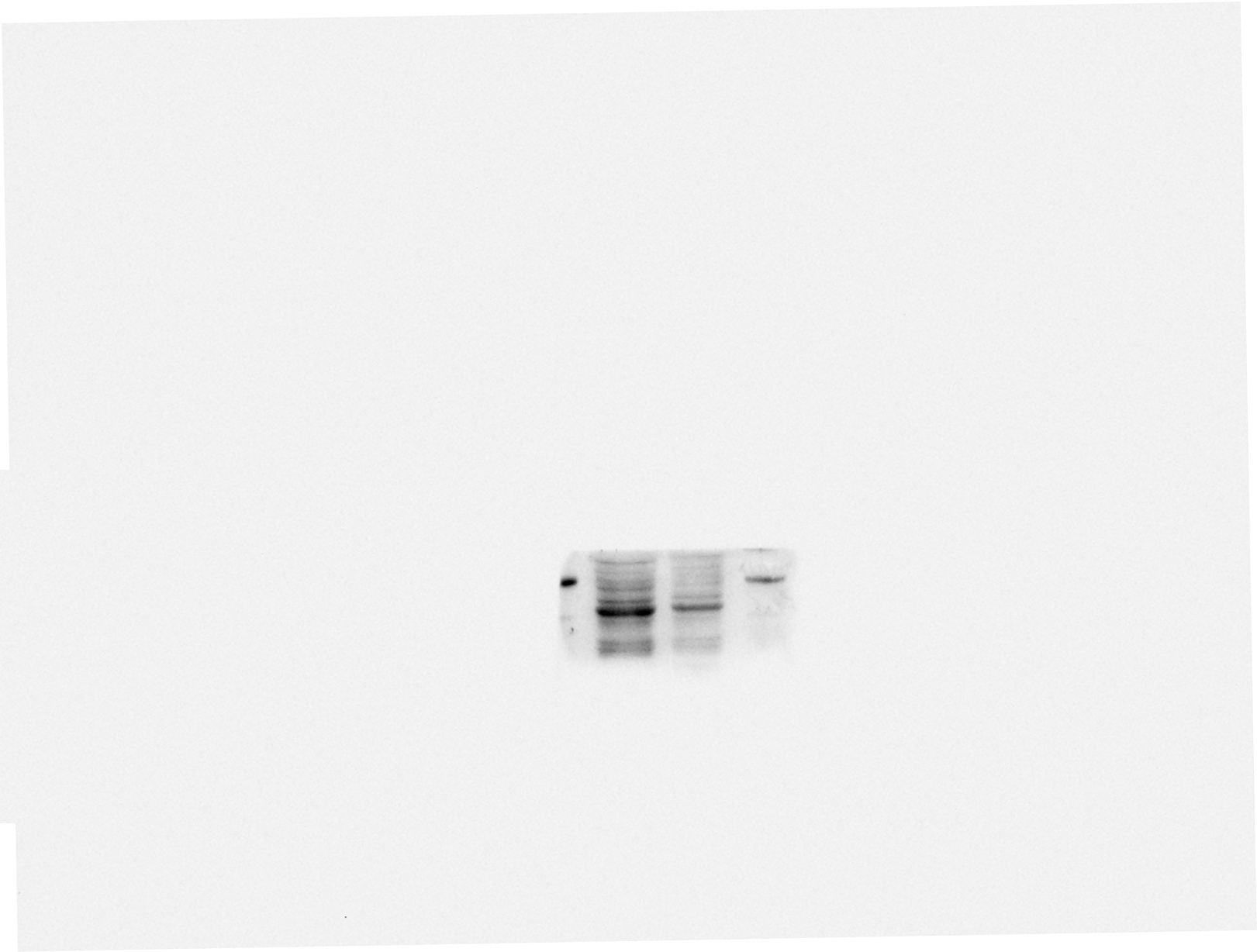

Supplement: Supplementary file 4 [file DataSheet8.ZIP › F4D left up TAZ.jpg]

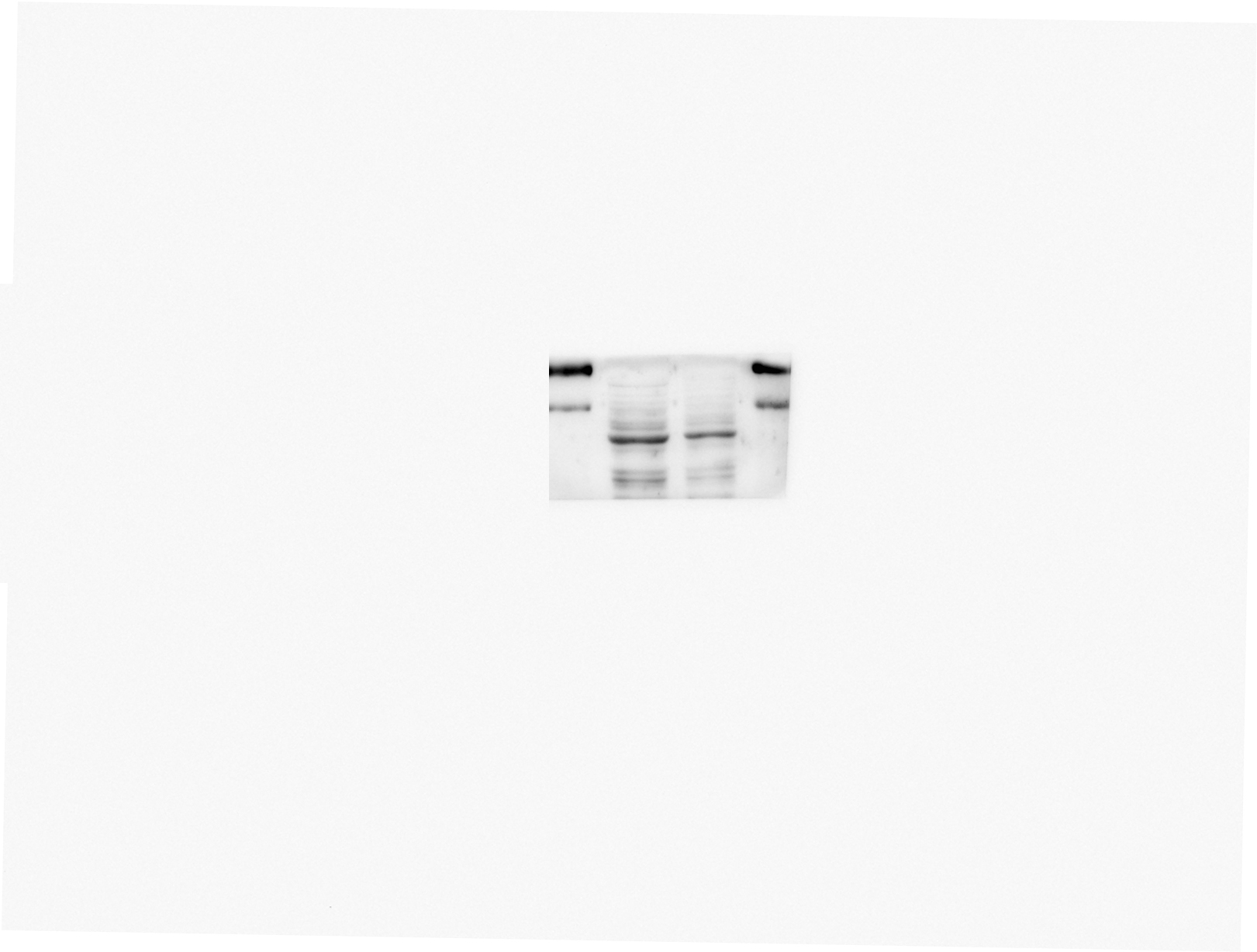

Supplement: Supplementary file 4 [file DataSheet8.ZIP › F4D left up vimentin.jpg]

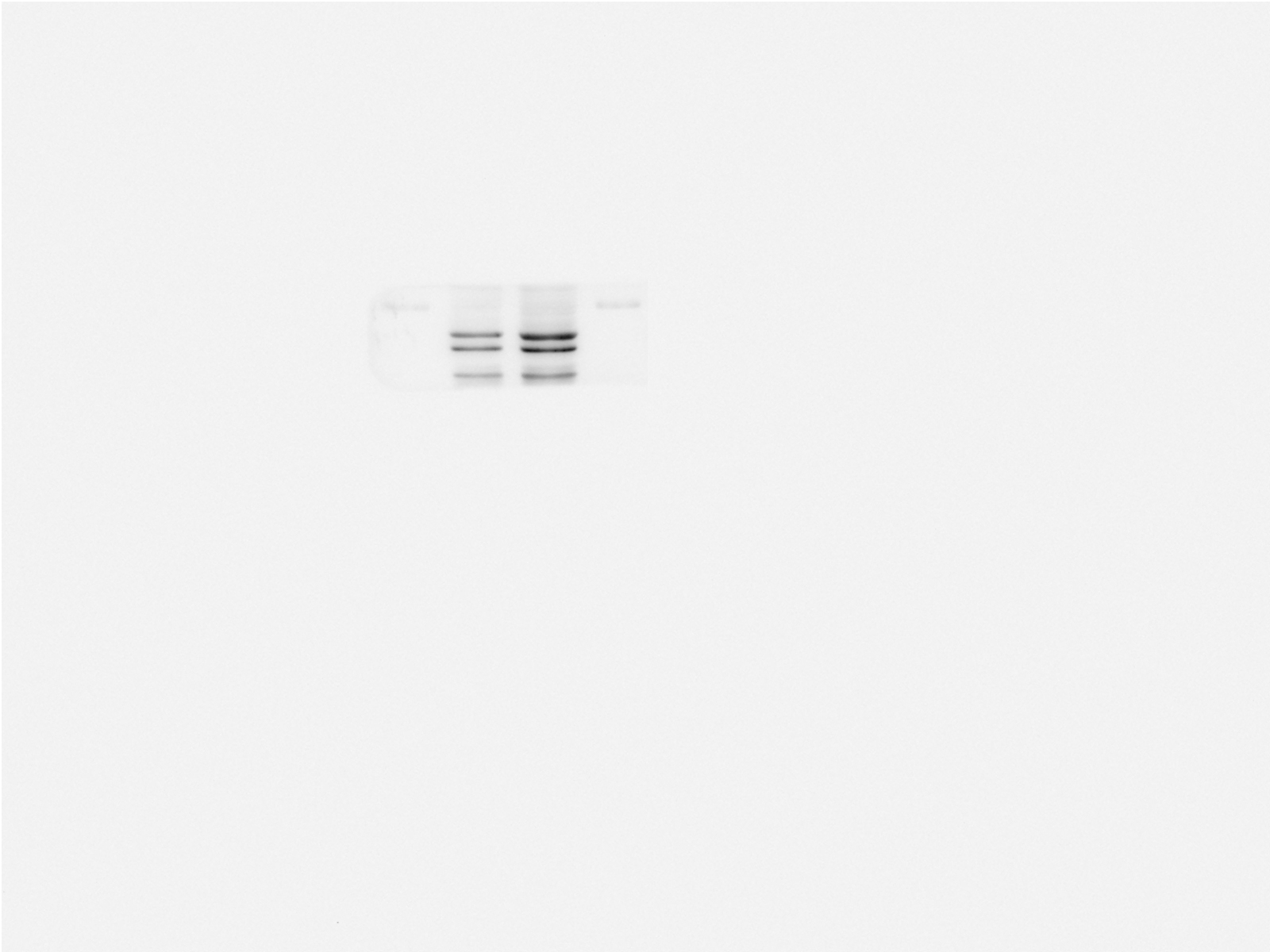

Supplement: Supplementary file 4 [file DataSheet8.ZIP › F4D right down cyclin E.jpg]

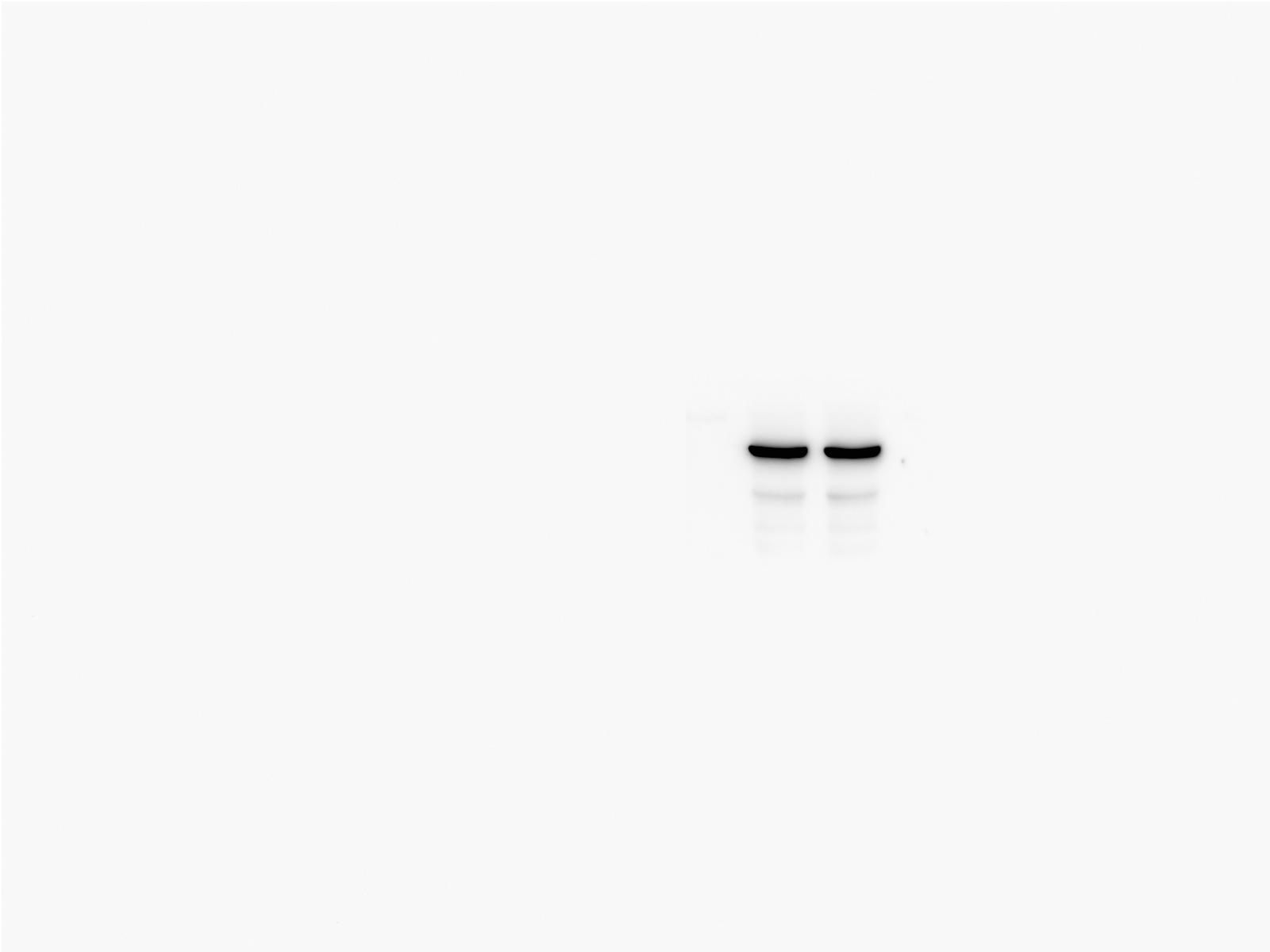

Supplement: Supplementary file 4 [file DataSheet8.ZIP › F4D right down actin.jpg]

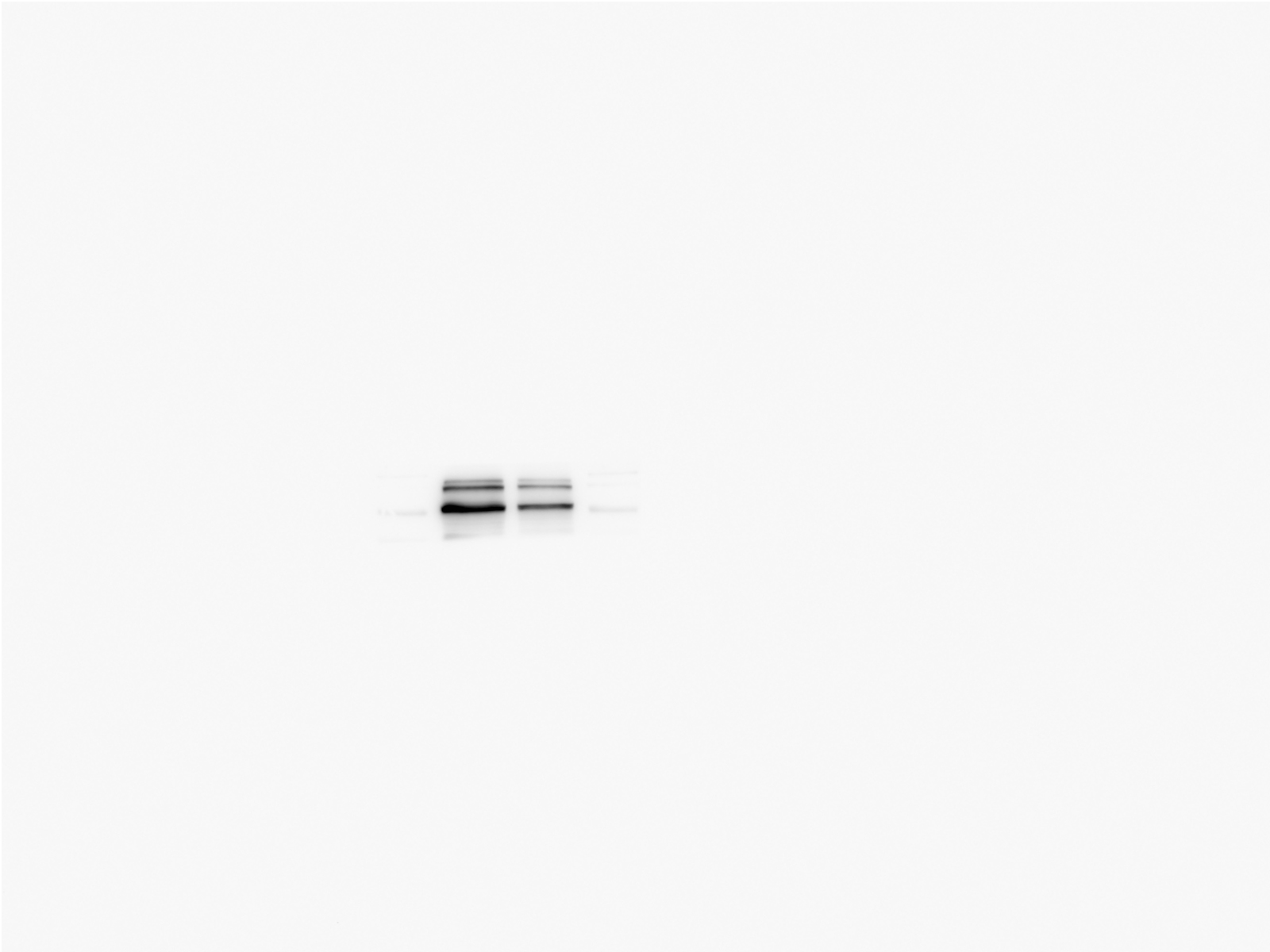

Supplement: Supplementary file 4 [file DataSheet8.ZIP › F4D right down E-Cadherin.jpg]

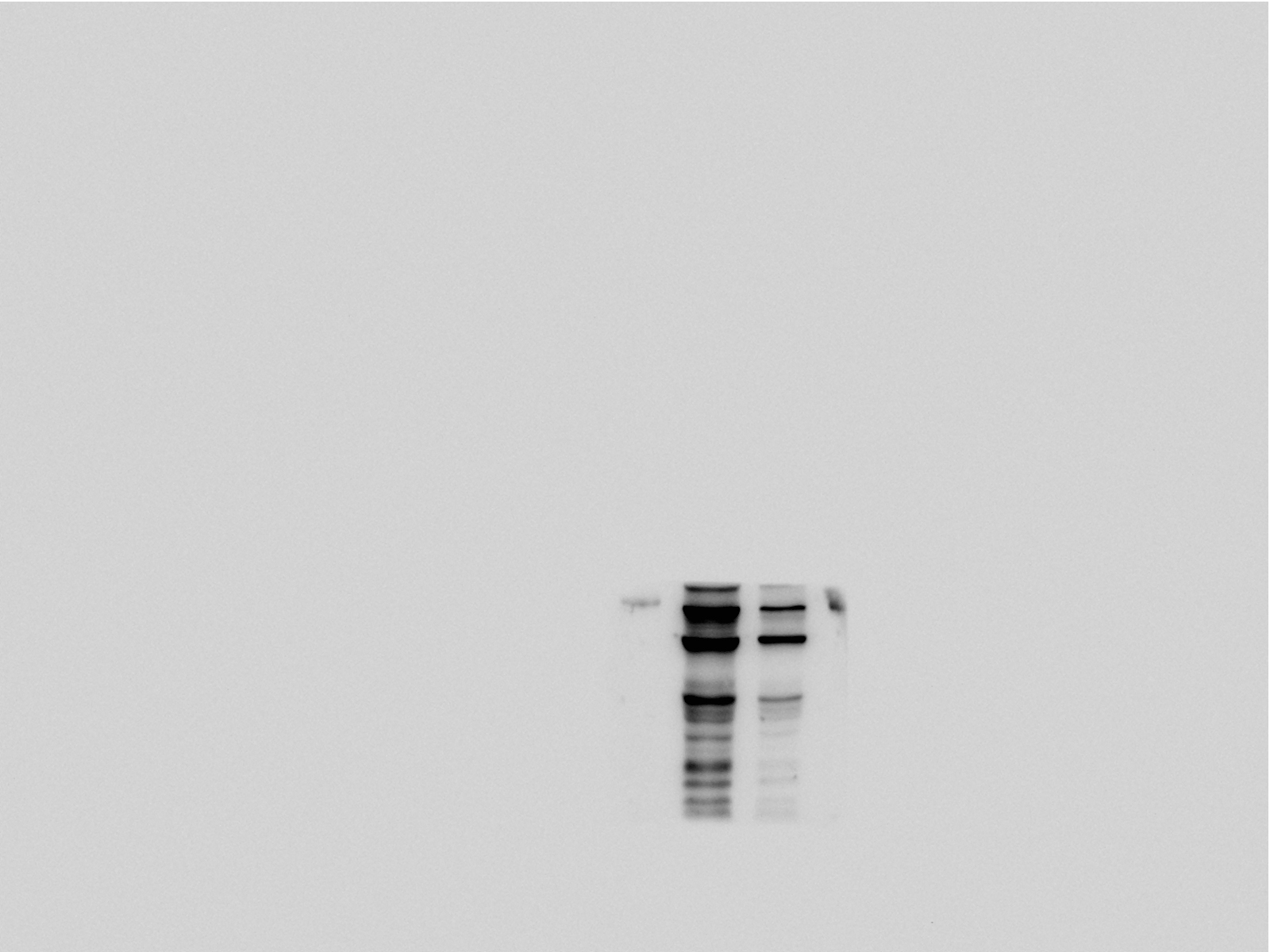

Supplement: Supplementary file 4 [file DataSheet8.ZIP › F4D right down p27.jpg]

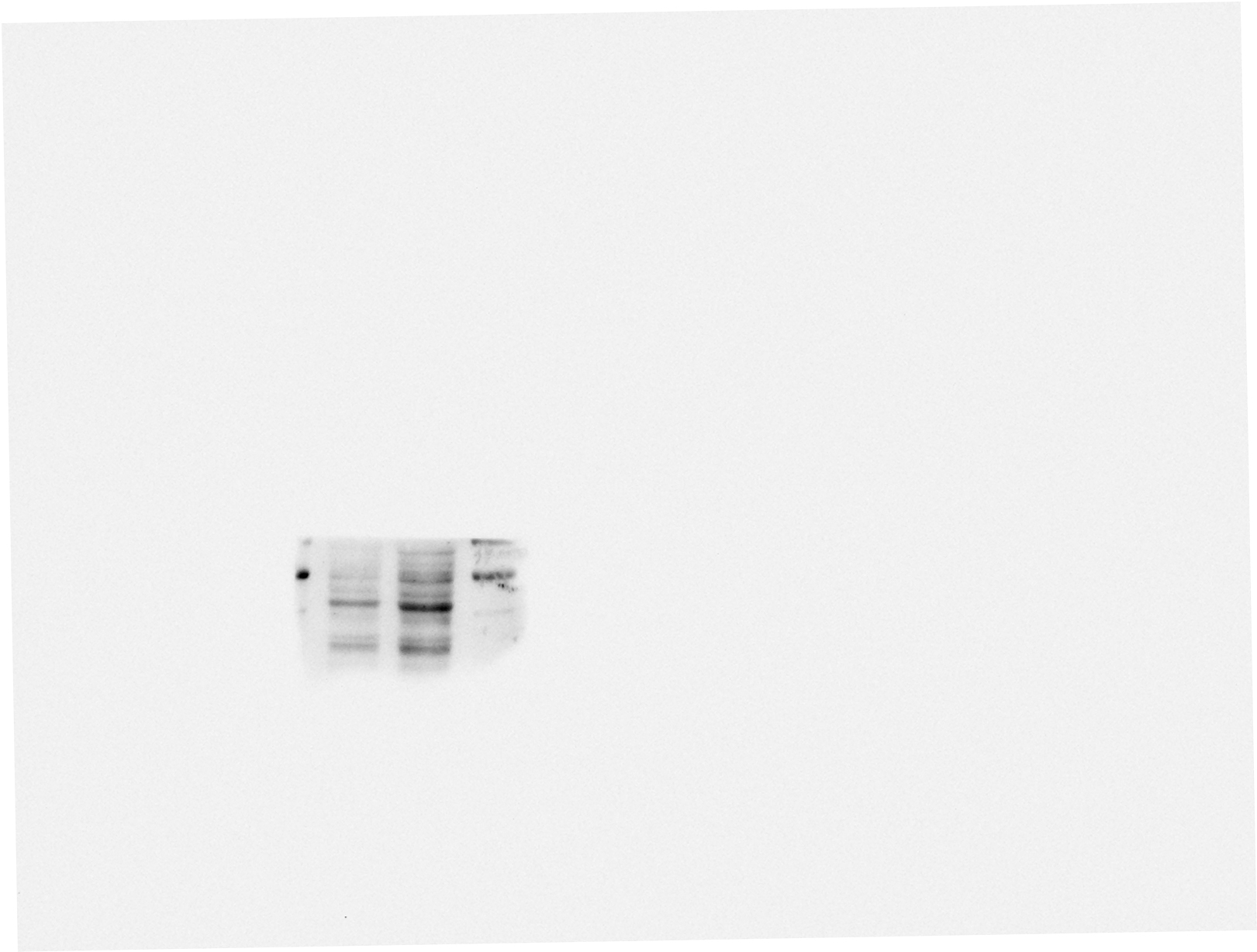

Supplement: Supplementary file 4 [file DataSheet8.ZIP › F4D right down TAZ.jpg]

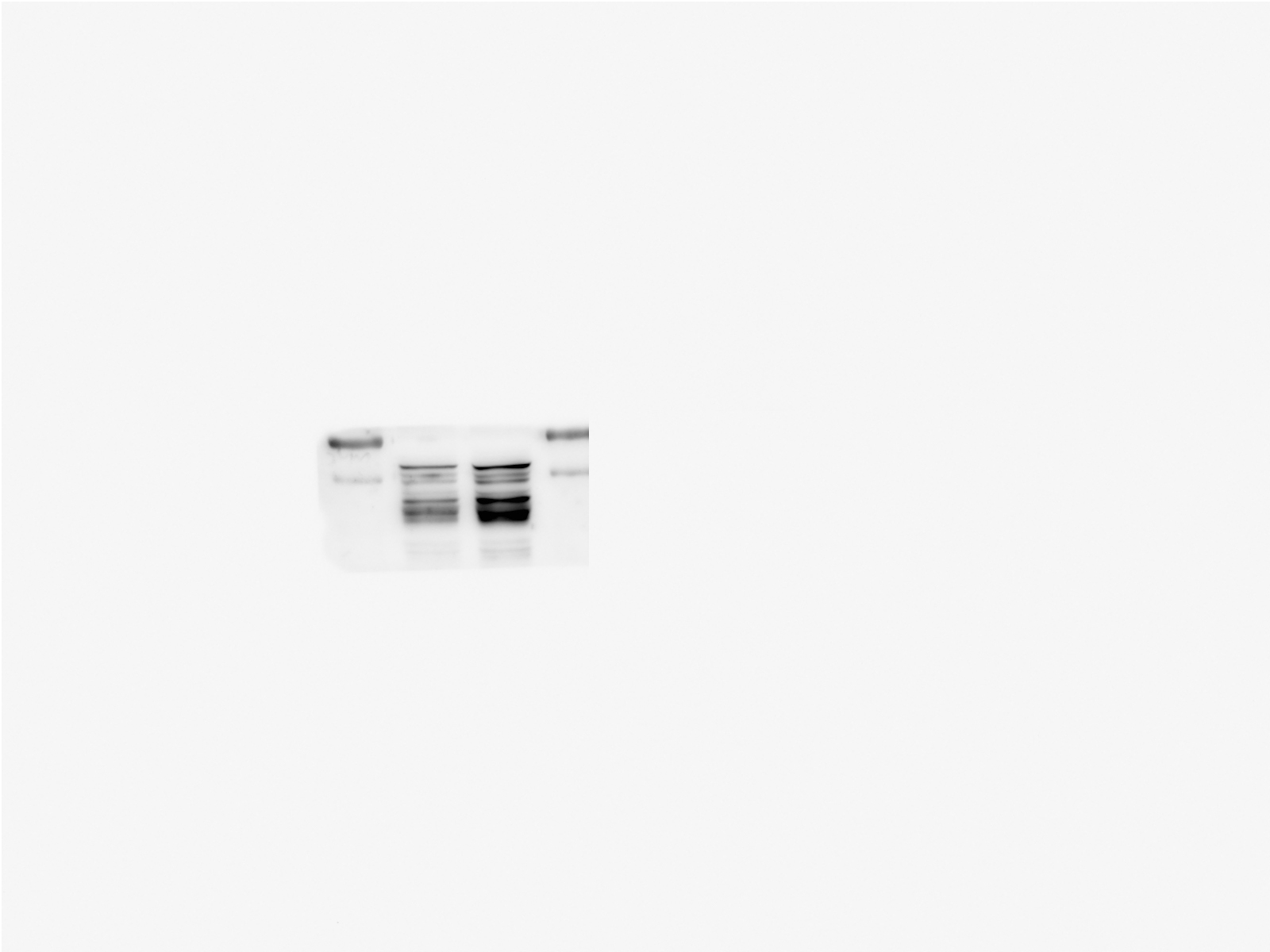

Supplement: Supplementary file 4 [file DataSheet8.ZIP › F4D right down vimentin.jpg]

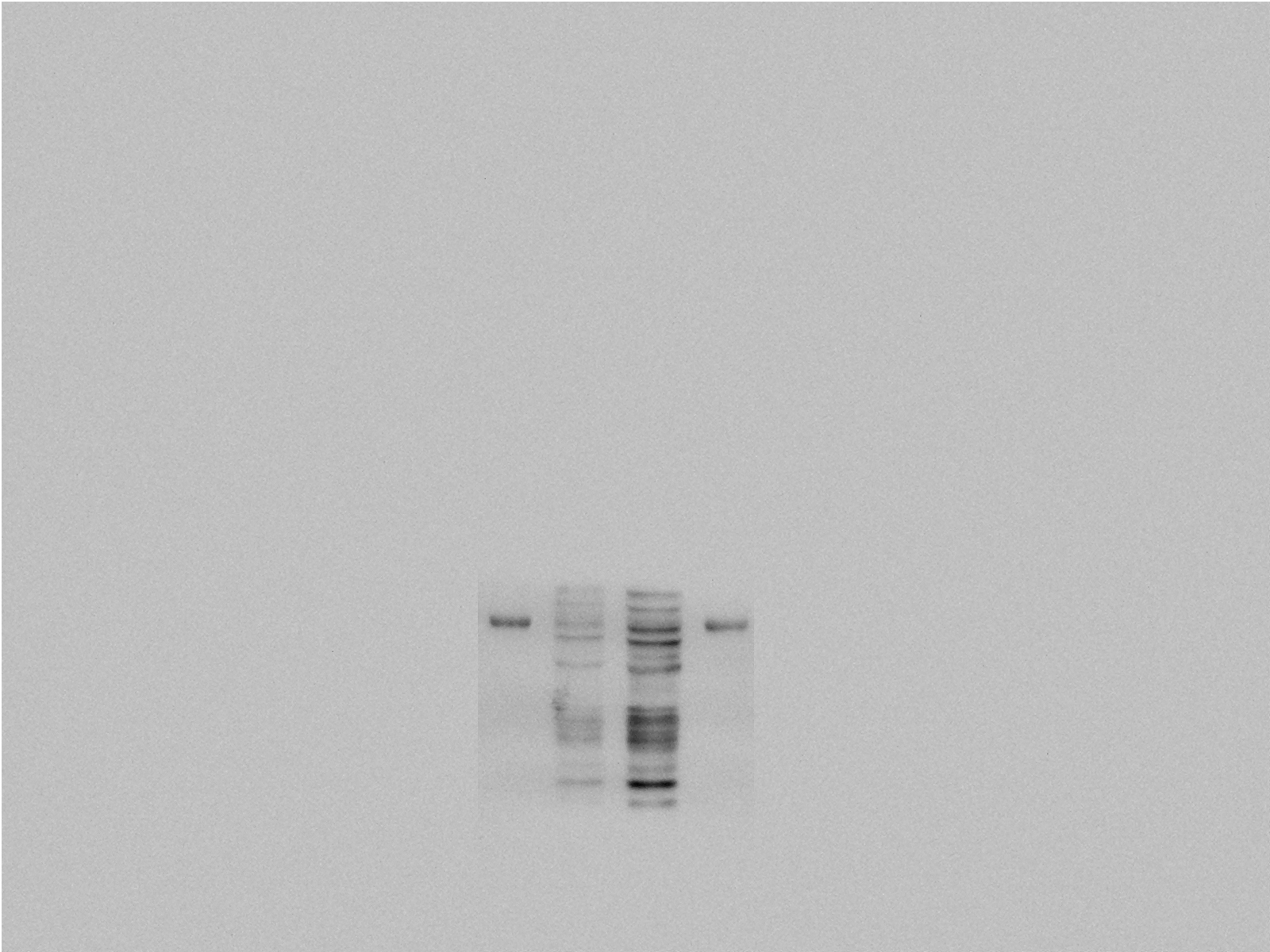

Supplement: Supplementary file 4 [file DataSheet8.ZIP › F4D right up cyclin E.jpg]

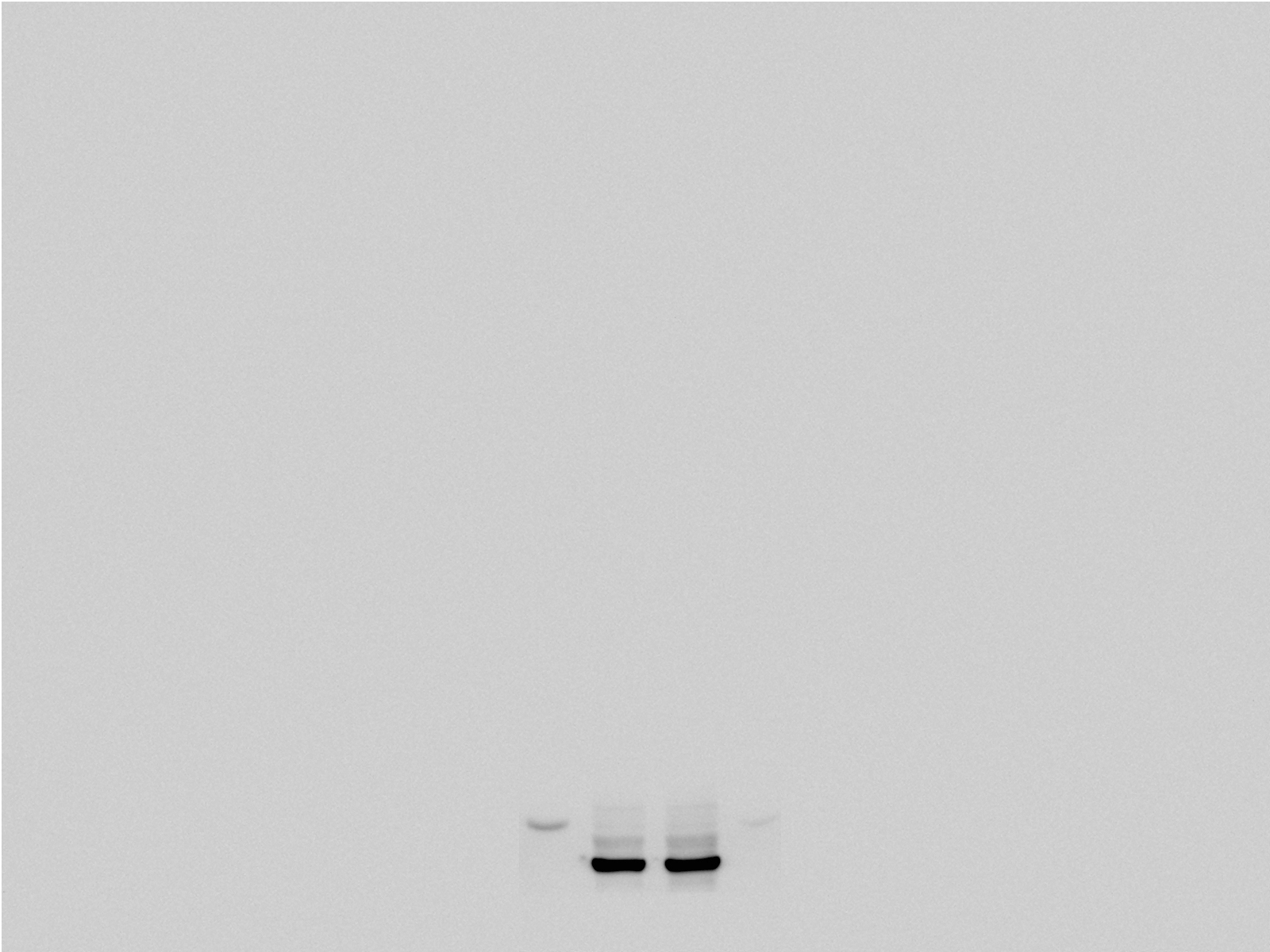

Supplement: Supplementary file 4 [file DataSheet8.ZIP › F4D right up actin.jpg]

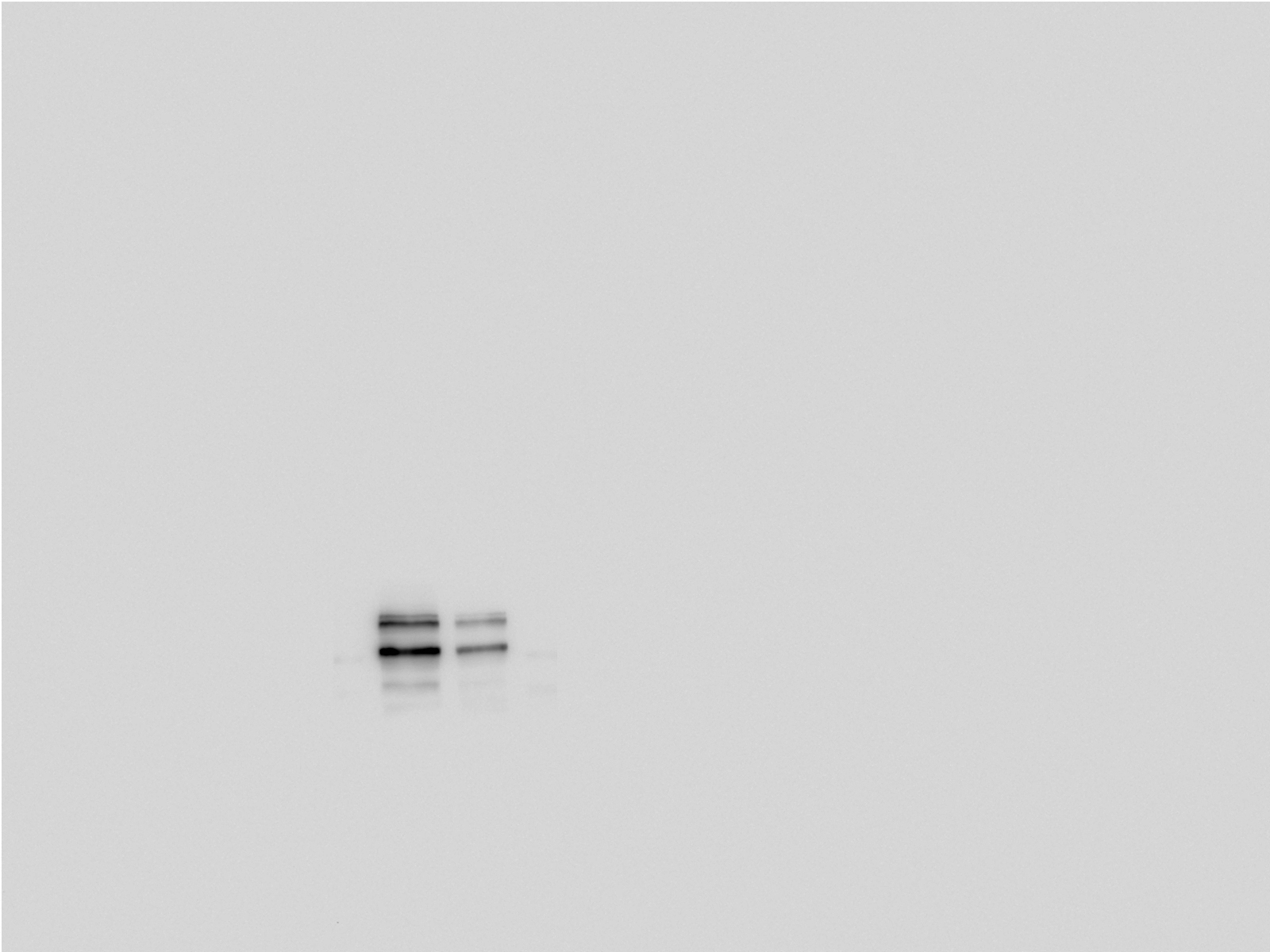

Supplement: Supplementary file 4 [file DataSheet8.ZIP › F4D right up E-Cadherin.jpg]

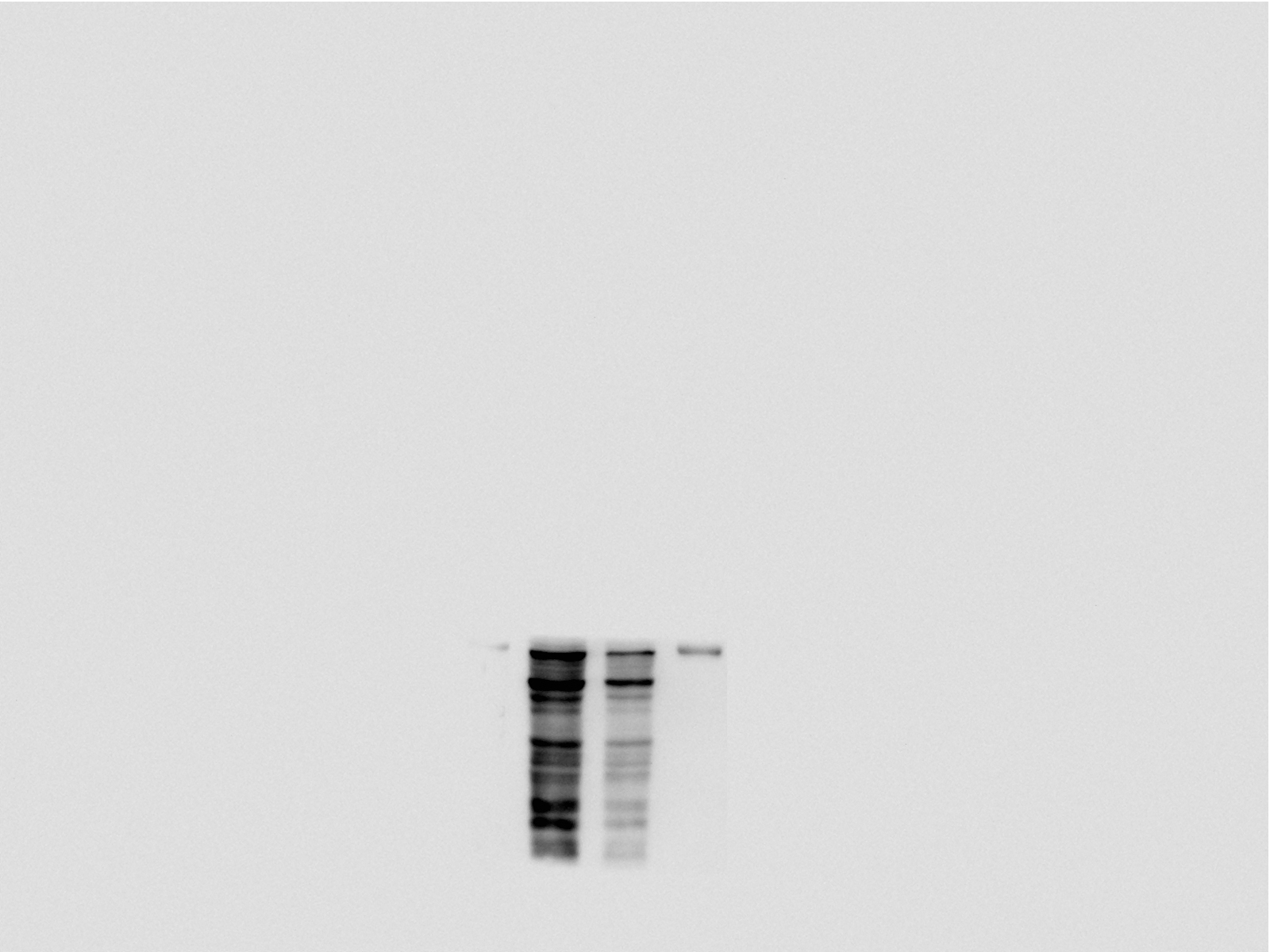

Supplement: Supplementary file 4 [file DataSheet8.ZIP › F4D right up p27.jpg]

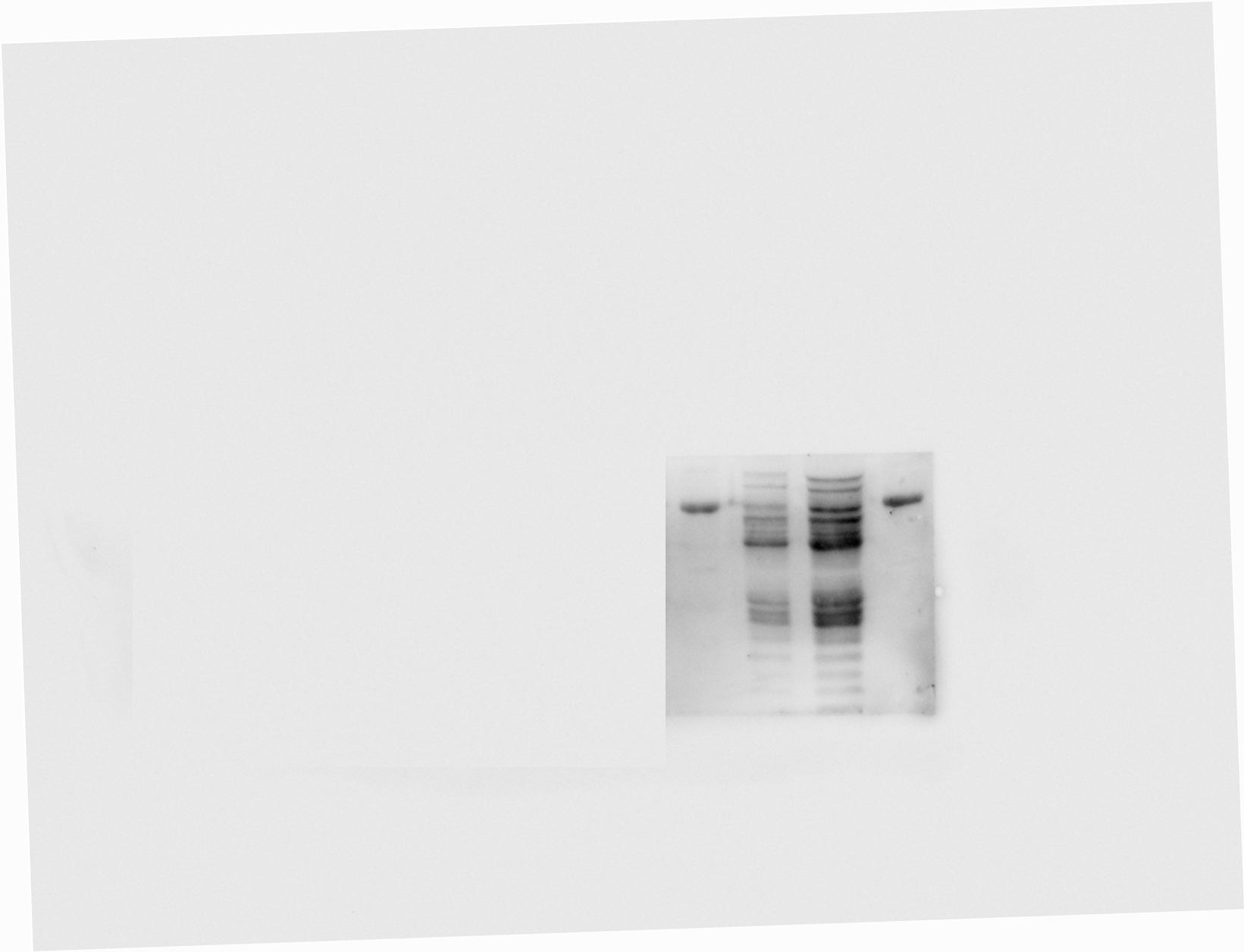

Supplement: Supplementary file 4 [file DataSheet8.ZIP › F4D right up taz.jpg]

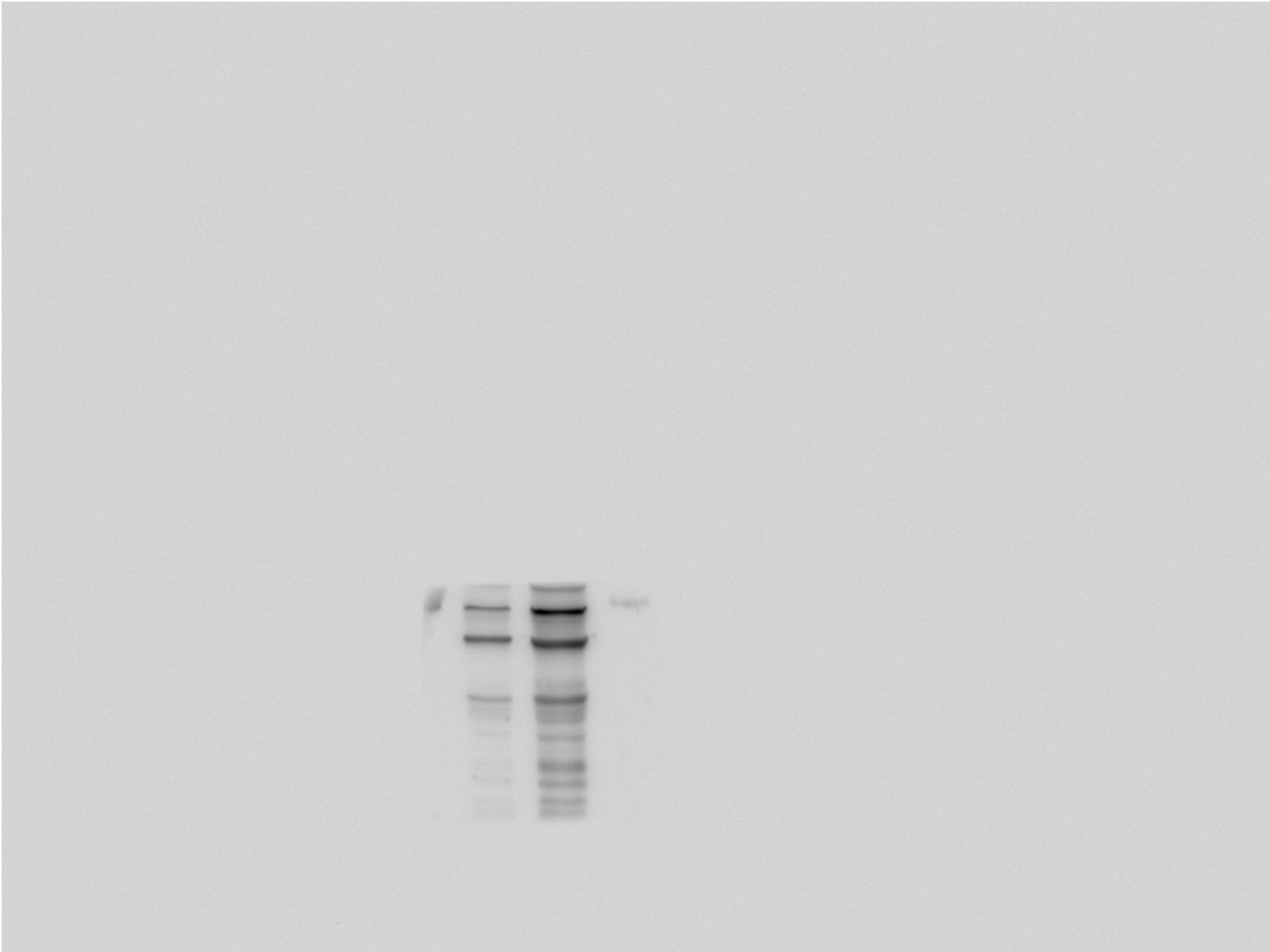

Supplement: Supplementary file 4 [file DataSheet8.ZIP › F4D right up vimentin.jpg]

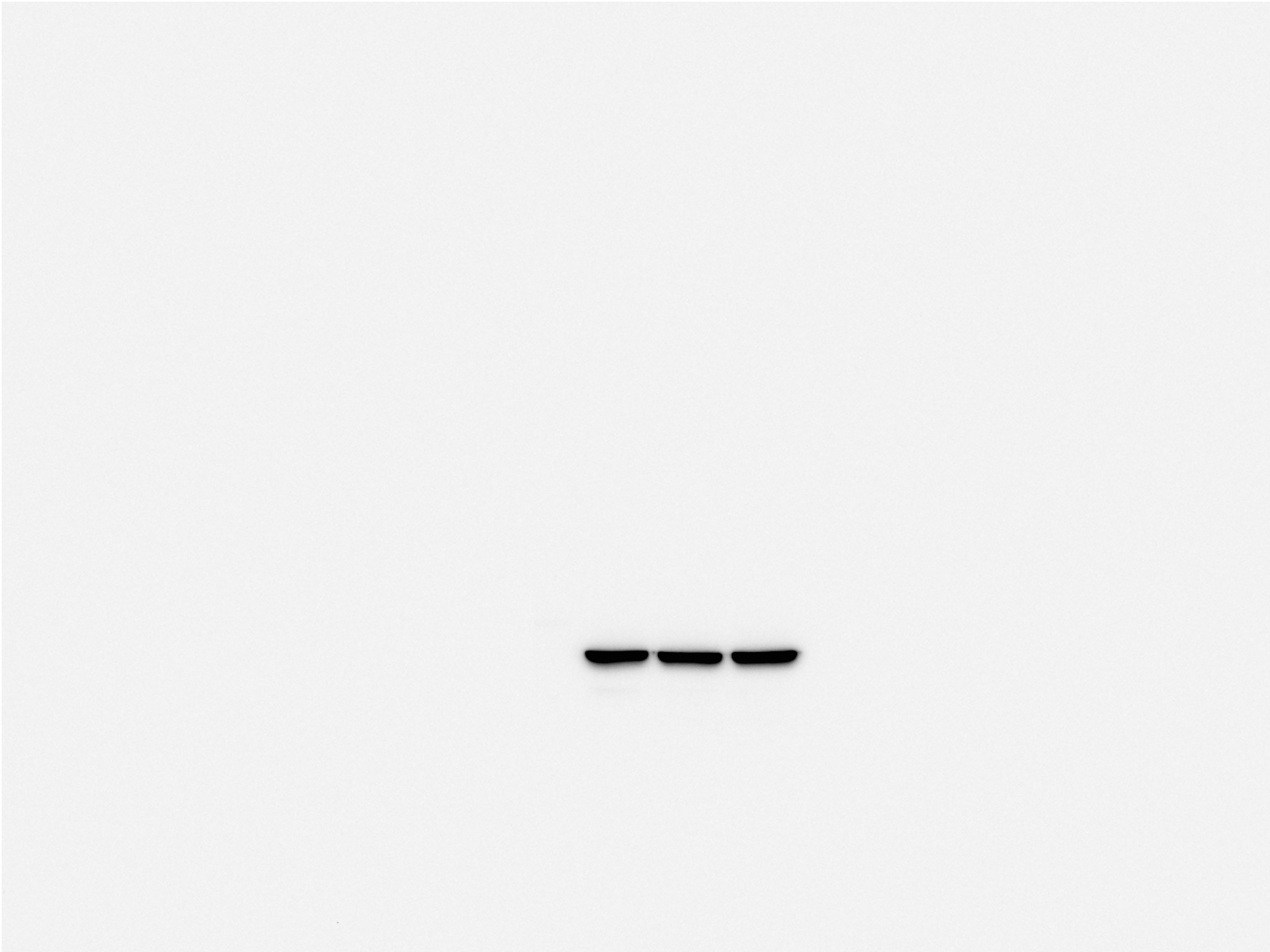

Supplement: Supplementary file 4 [file DataSheet8.ZIP › F5D actin.jpg]

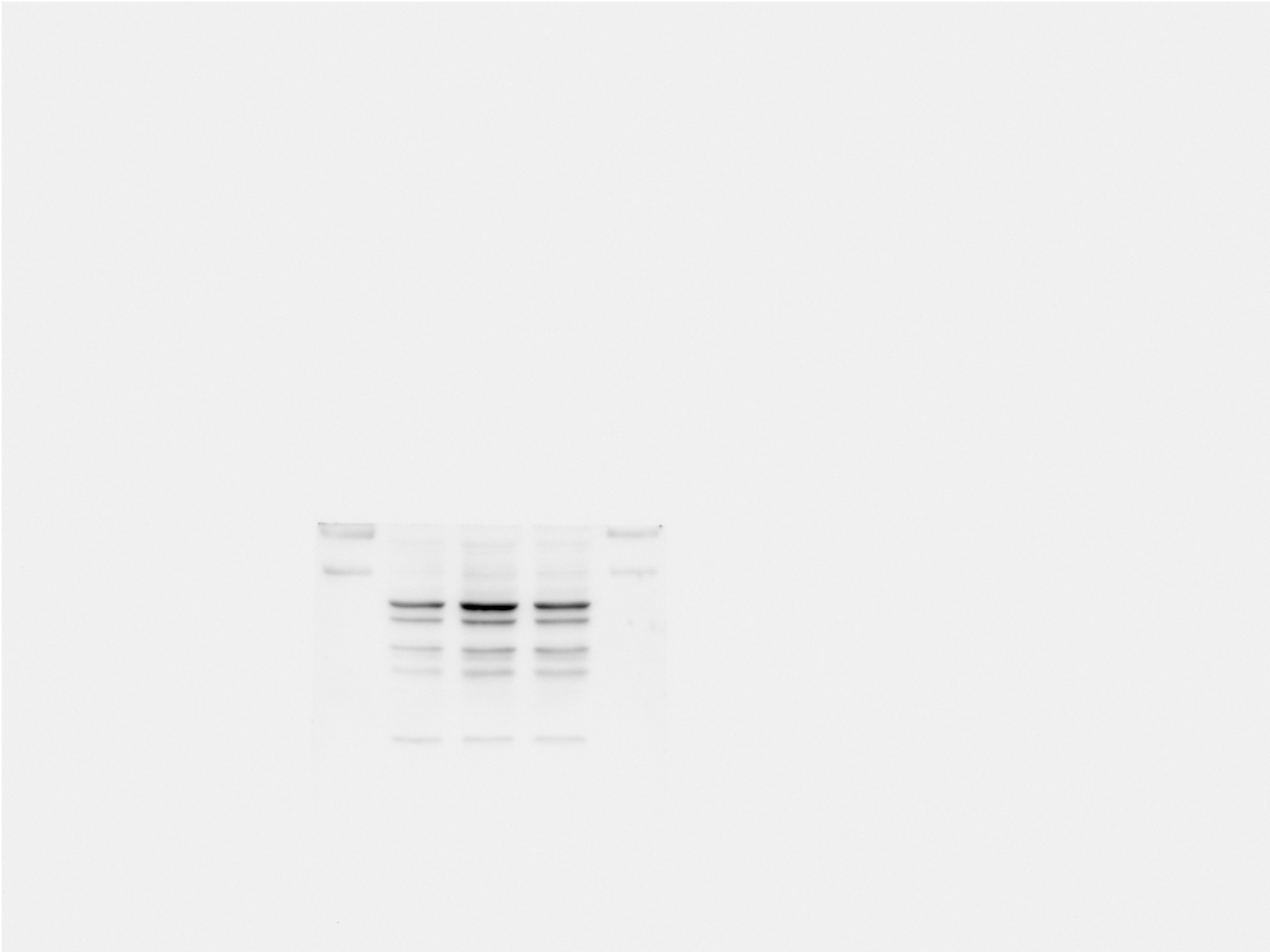

Supplement: Supplementary file 4 [file DataSheet8.ZIP › F5D cyclin E.jpg]

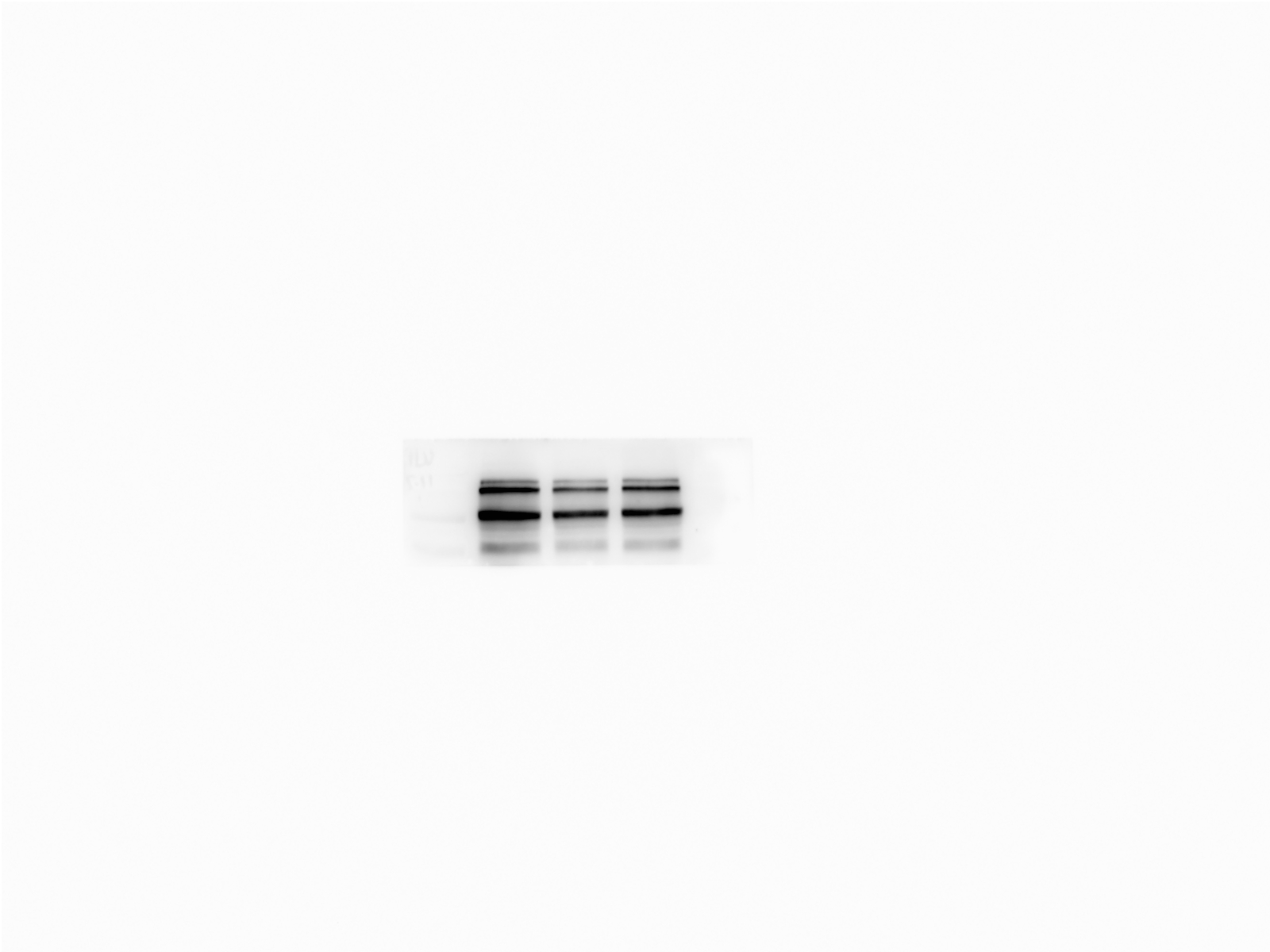

Supplement: Supplementary file 4 [file DataSheet8.ZIP › F5D E-Cadherin.jpg]

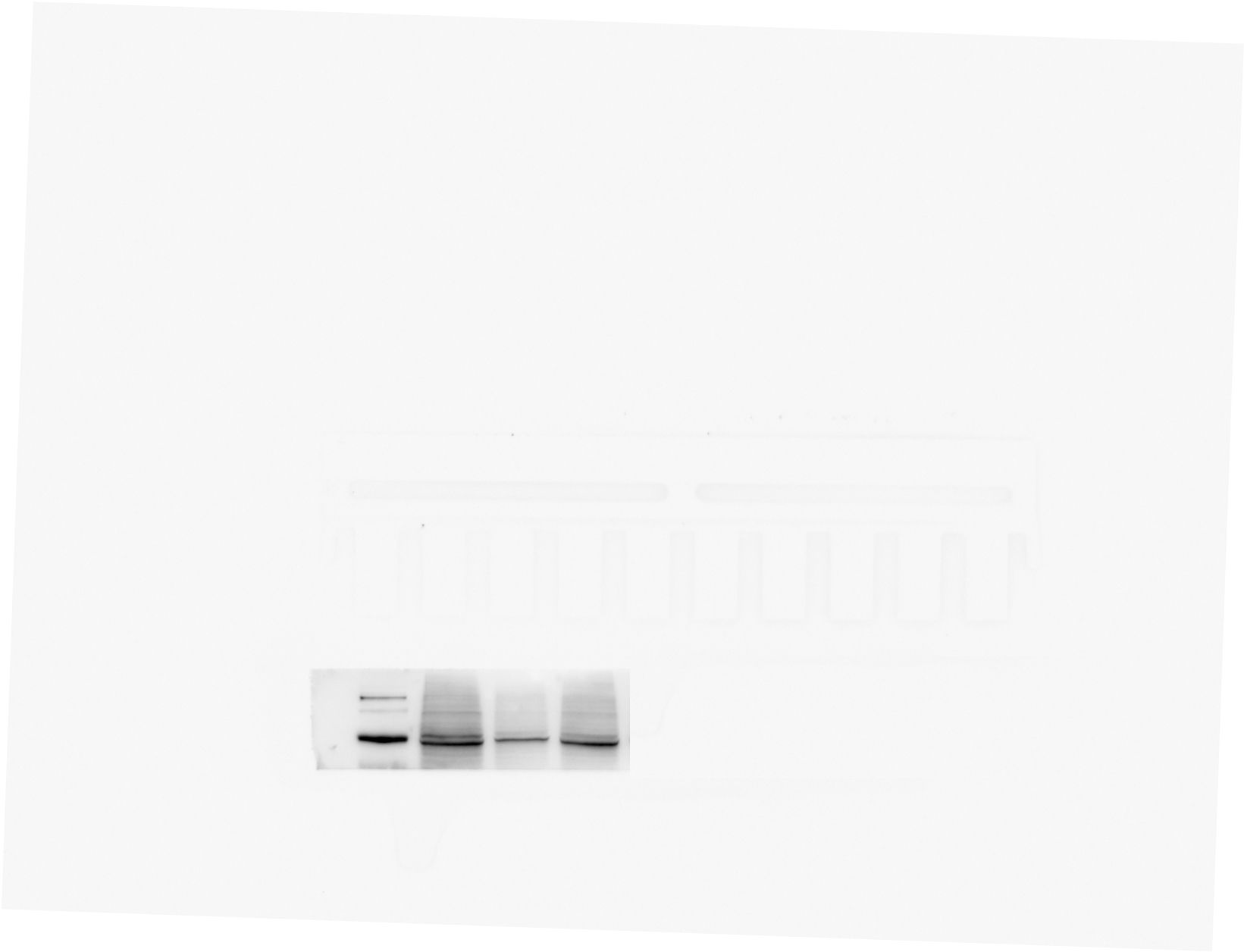

Supplement: Supplementary file 4 [file DataSheet8.ZIP › F5D lats1.jpg]

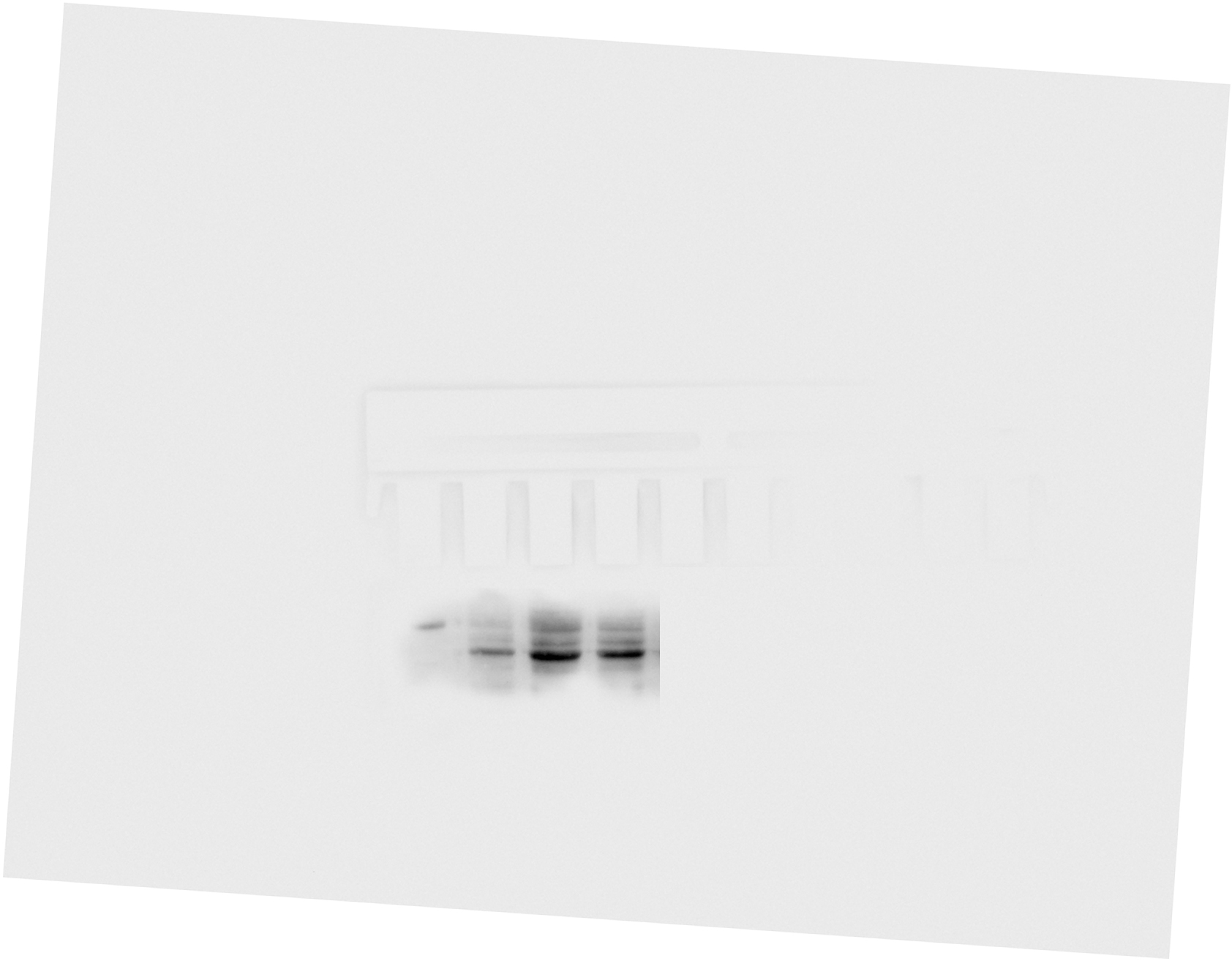

Supplement: Supplementary file 4 [file DataSheet8.ZIP › F5D TAZ.jpg]

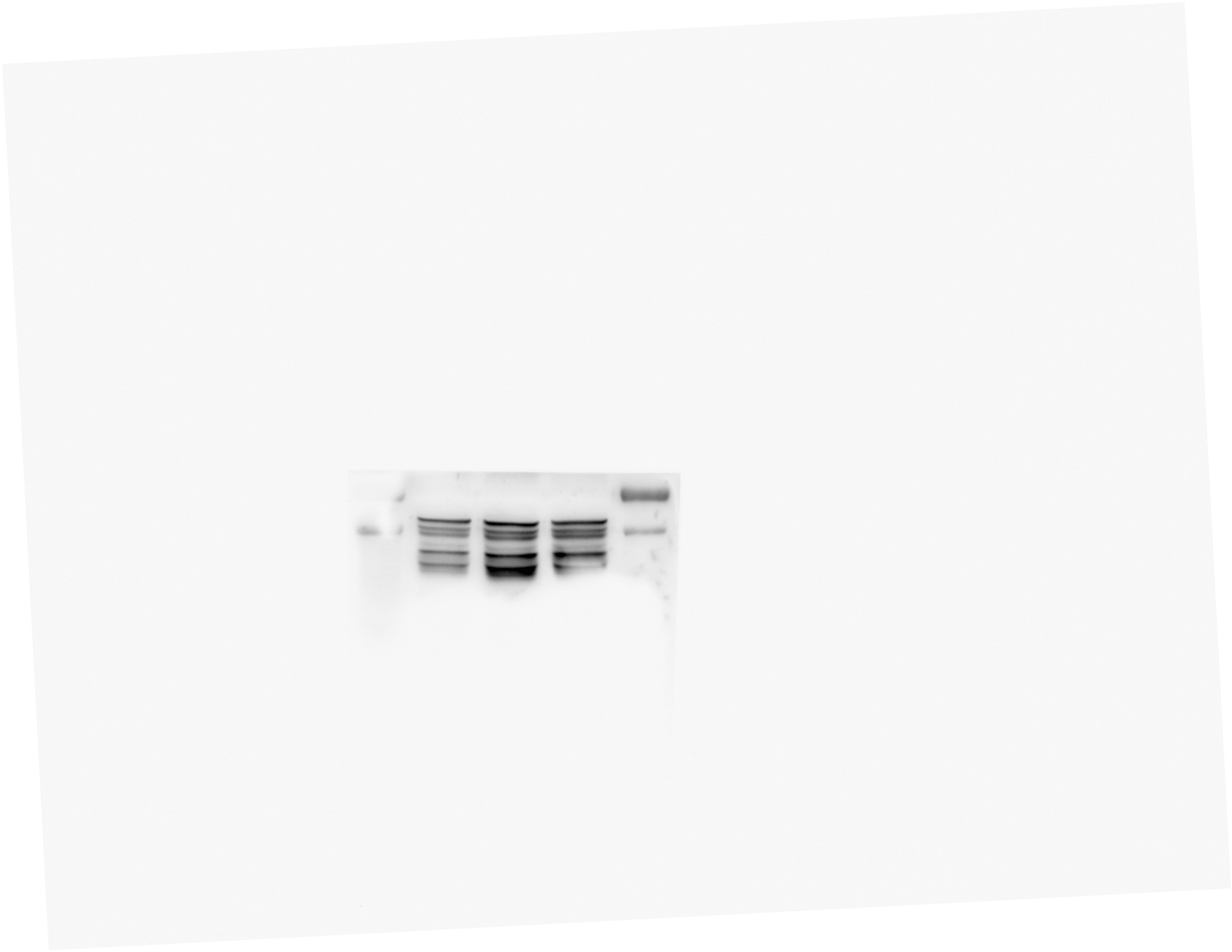

Supplement: Supplementary file 4 [file DataSheet8.ZIP › F5D vimentin.jpg]

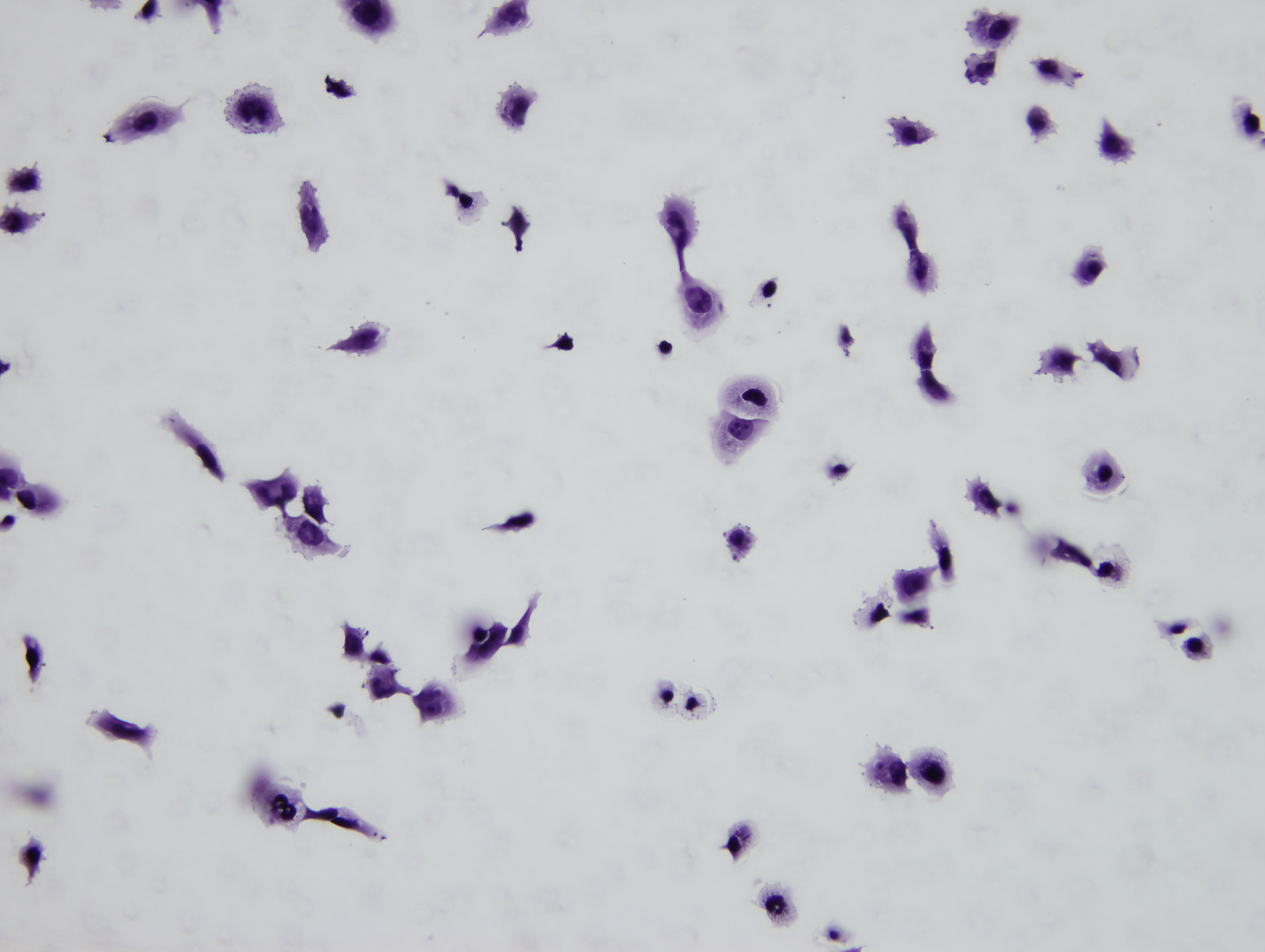

Supplement: Supplementary file 5 [file DataSheet4.ZIP › F4B left down Lats1.tif]

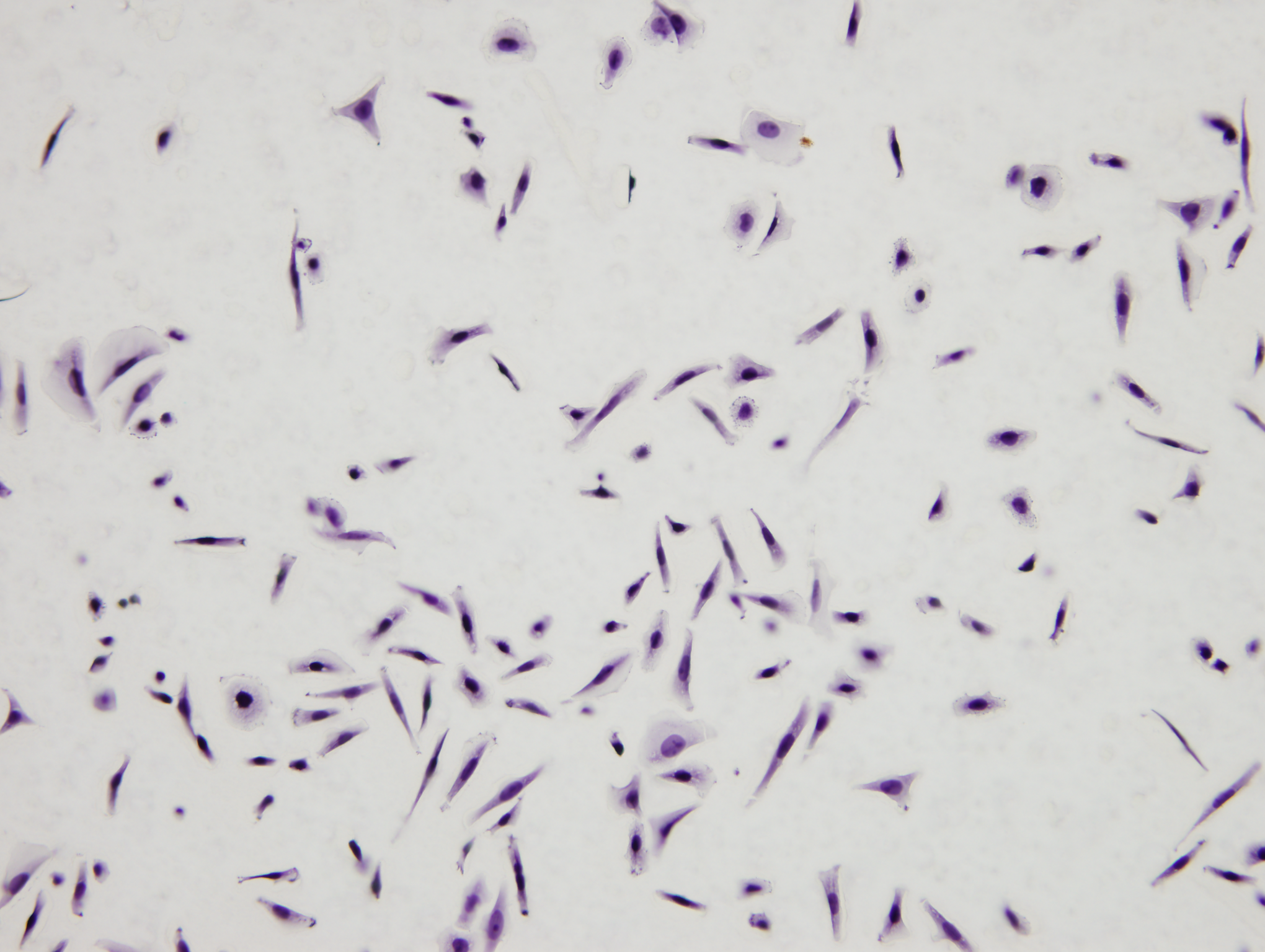

Supplement: Supplementary file 5 [file DataSheet4.ZIP › F4B left up control.tif]

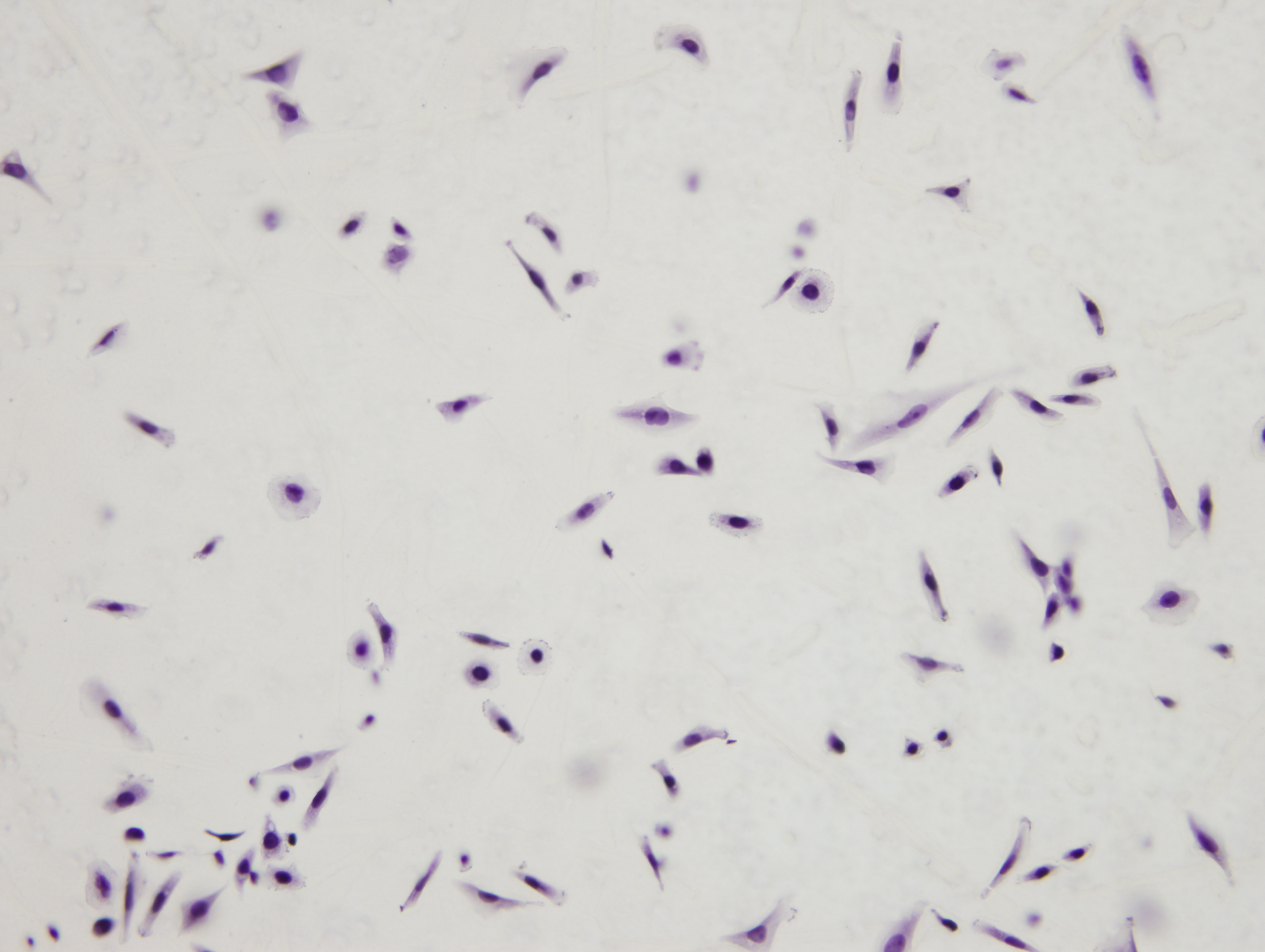

Supplement: Supplementary file 5 [file DataSheet4.ZIP › F4B left up Lats1.tif]

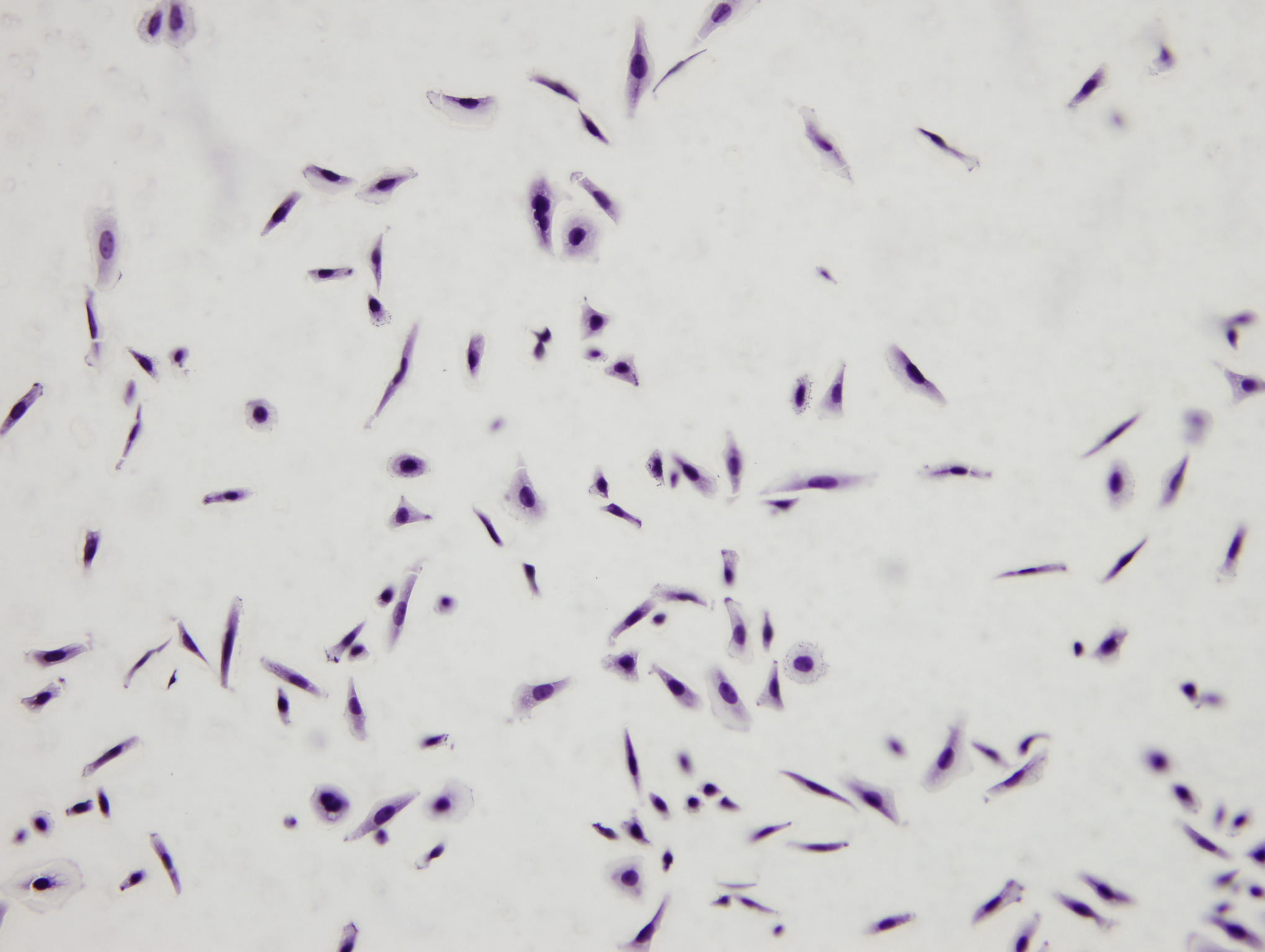

Supplement: Supplementary file 6 [file DataSheet1.ZIP › F2B left down control.tif]

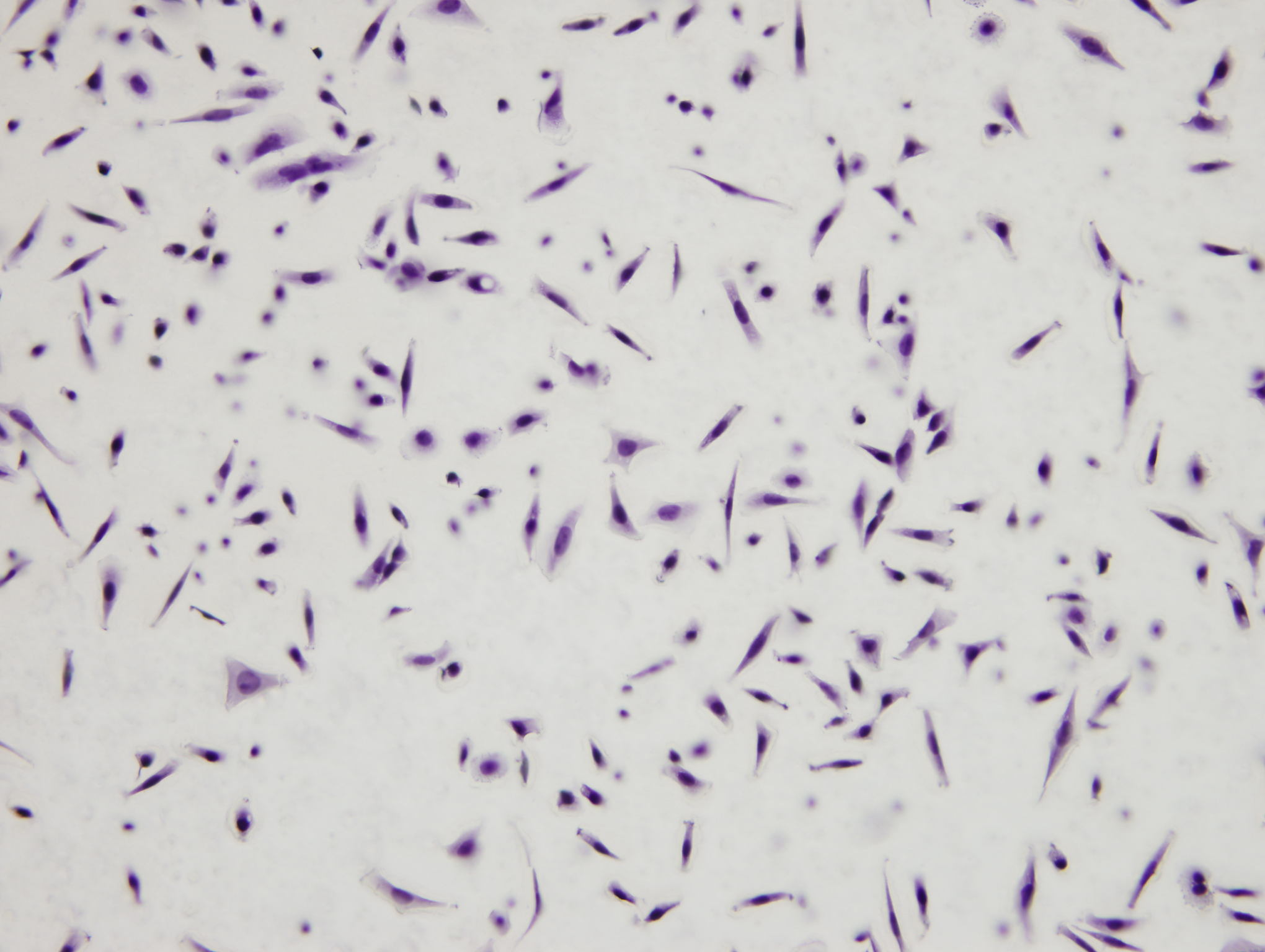

Supplement: Supplementary file 6 [file DataSheet1.ZIP › F2B left down miR-92a-3p mimic.tif]

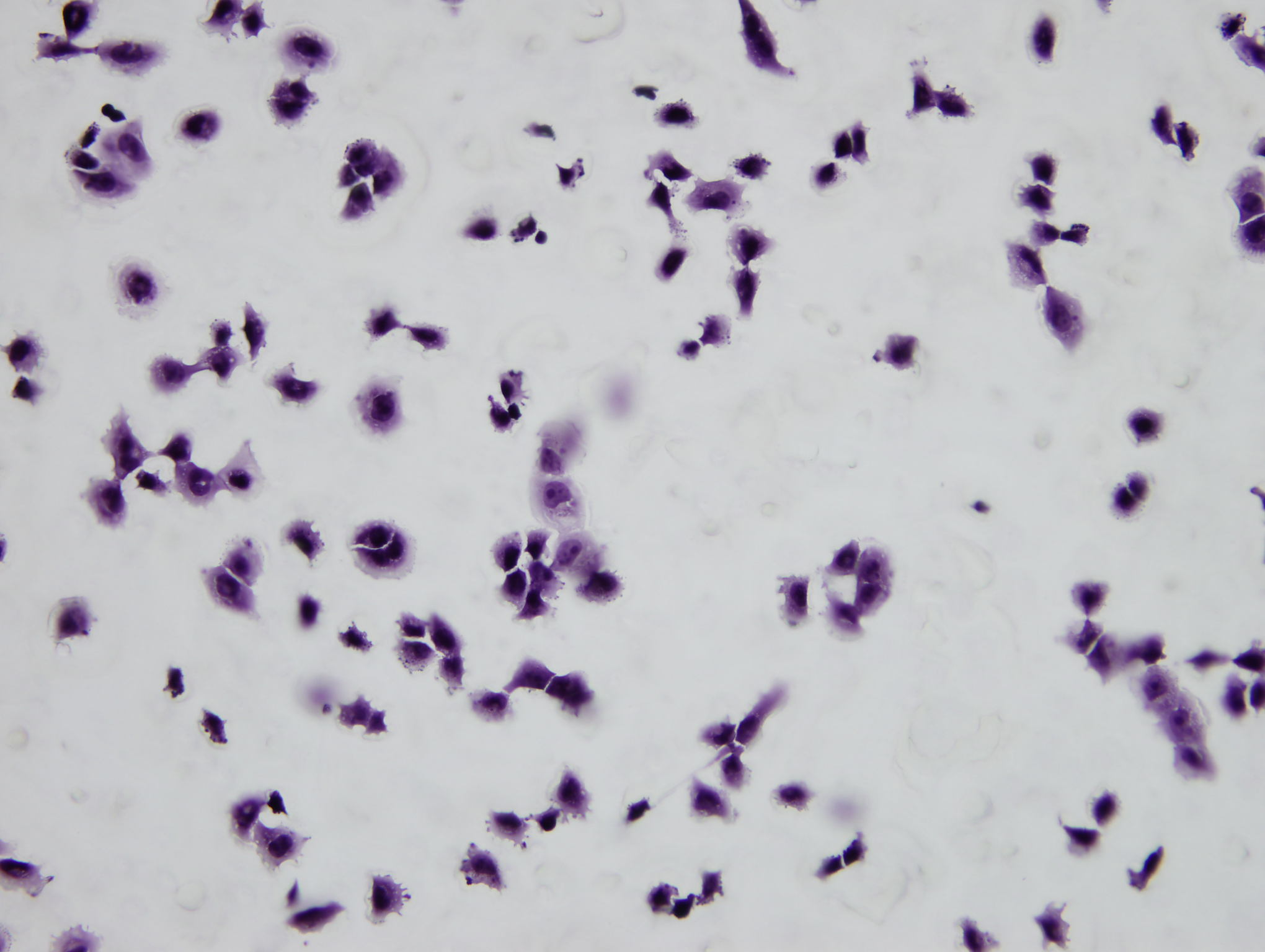

Supplement: Supplementary file 6 [file DataSheet1.ZIP › F2B left up control.tif]

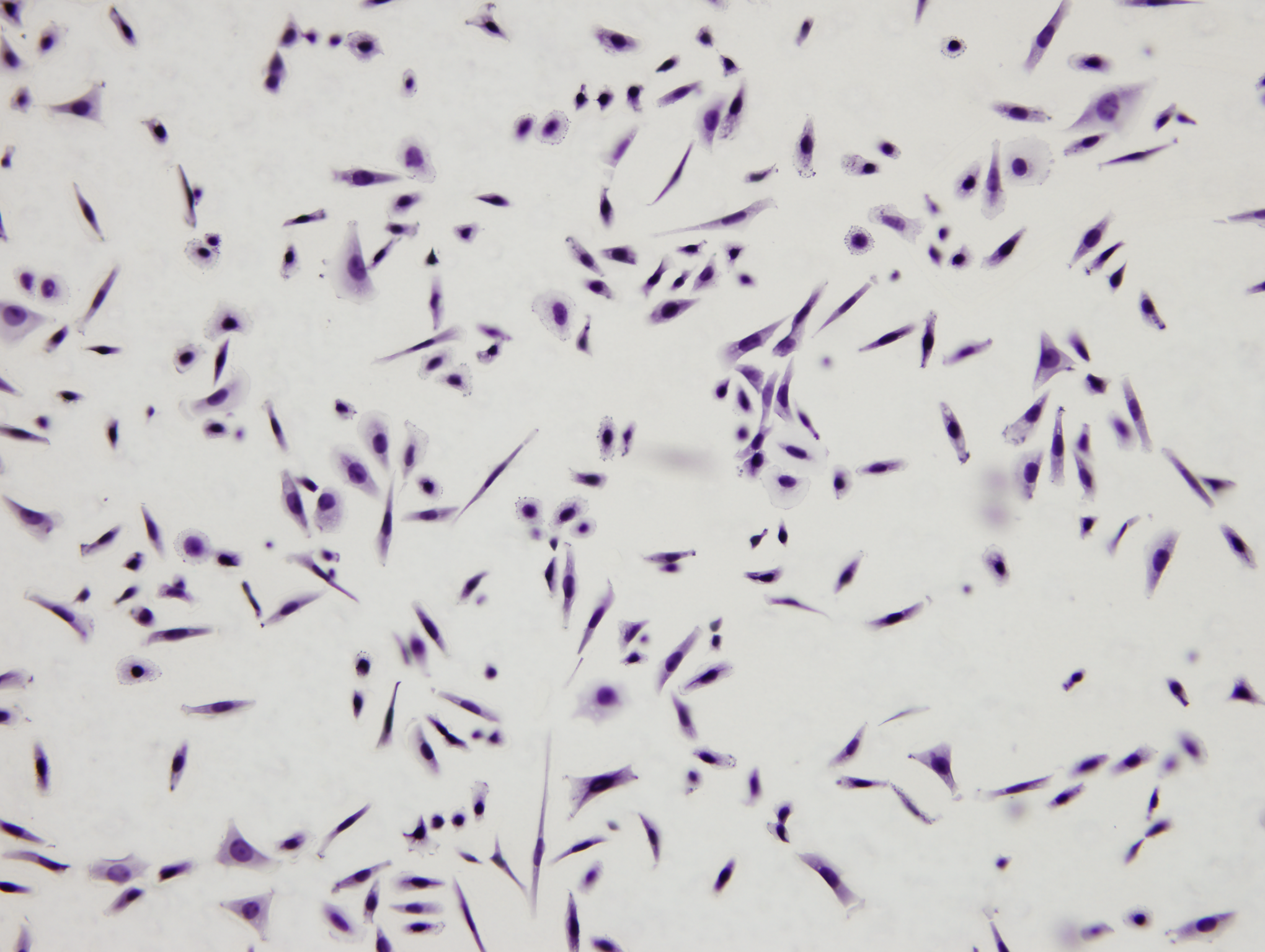

Supplement: Supplementary file 8 [file DataSheet6.ZIP › F4B right up siLats1.tif]

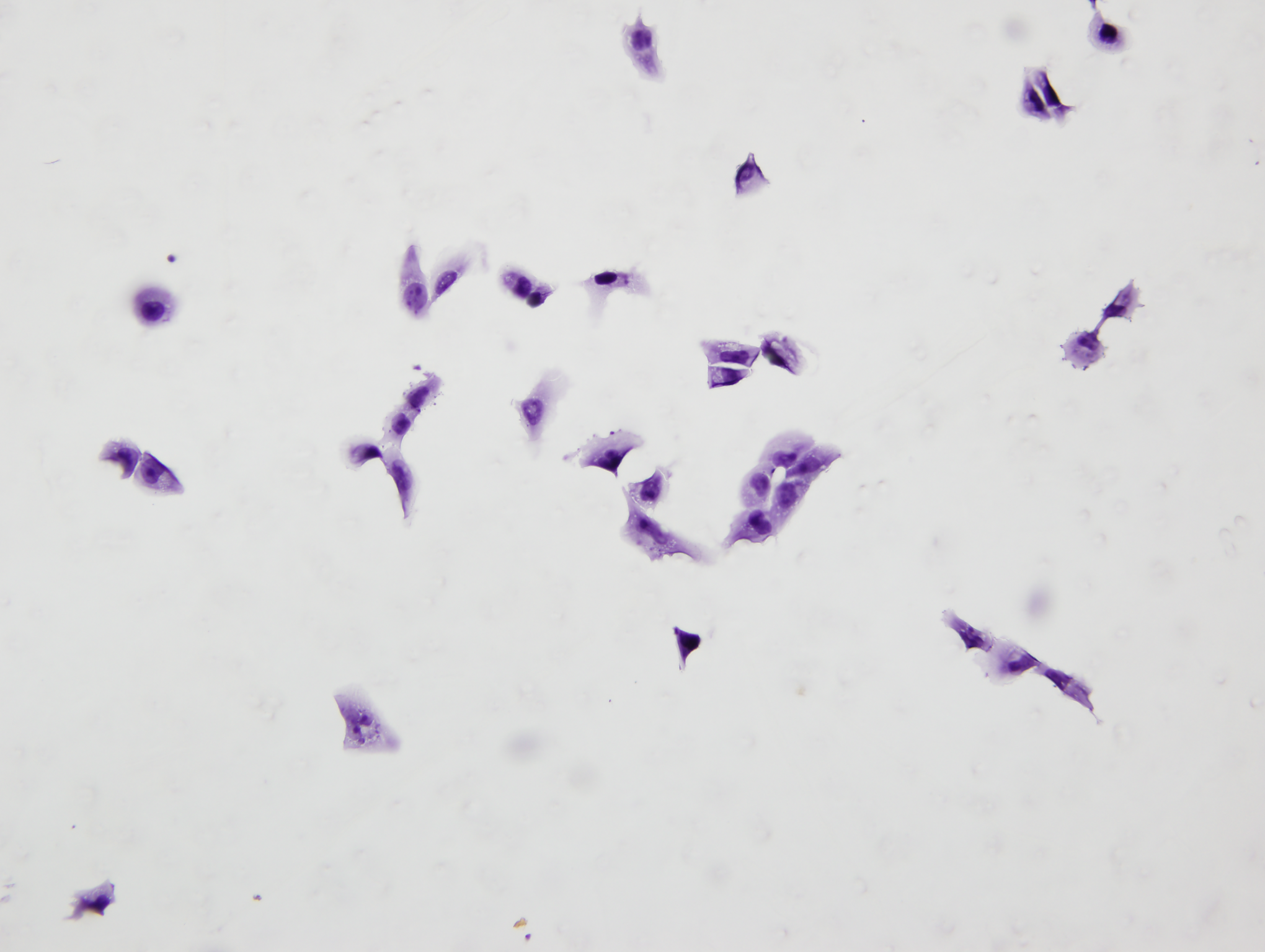

Supplement: Supplementary file 8 [file DataSheet6.ZIP › F5B left.tif]

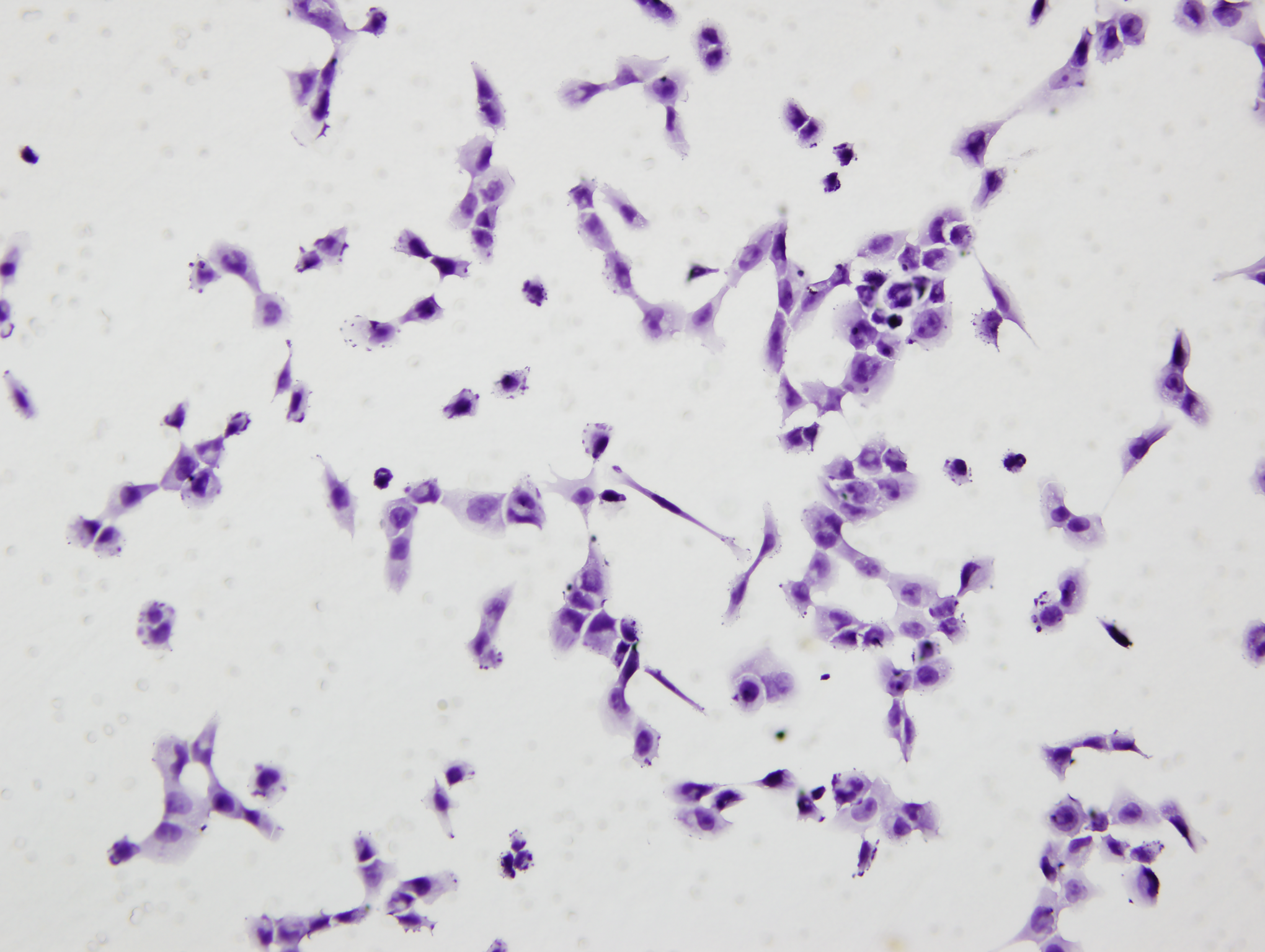

Supplement: Supplementary file 8 [file DataSheet6.ZIP › F5B middle.tif]

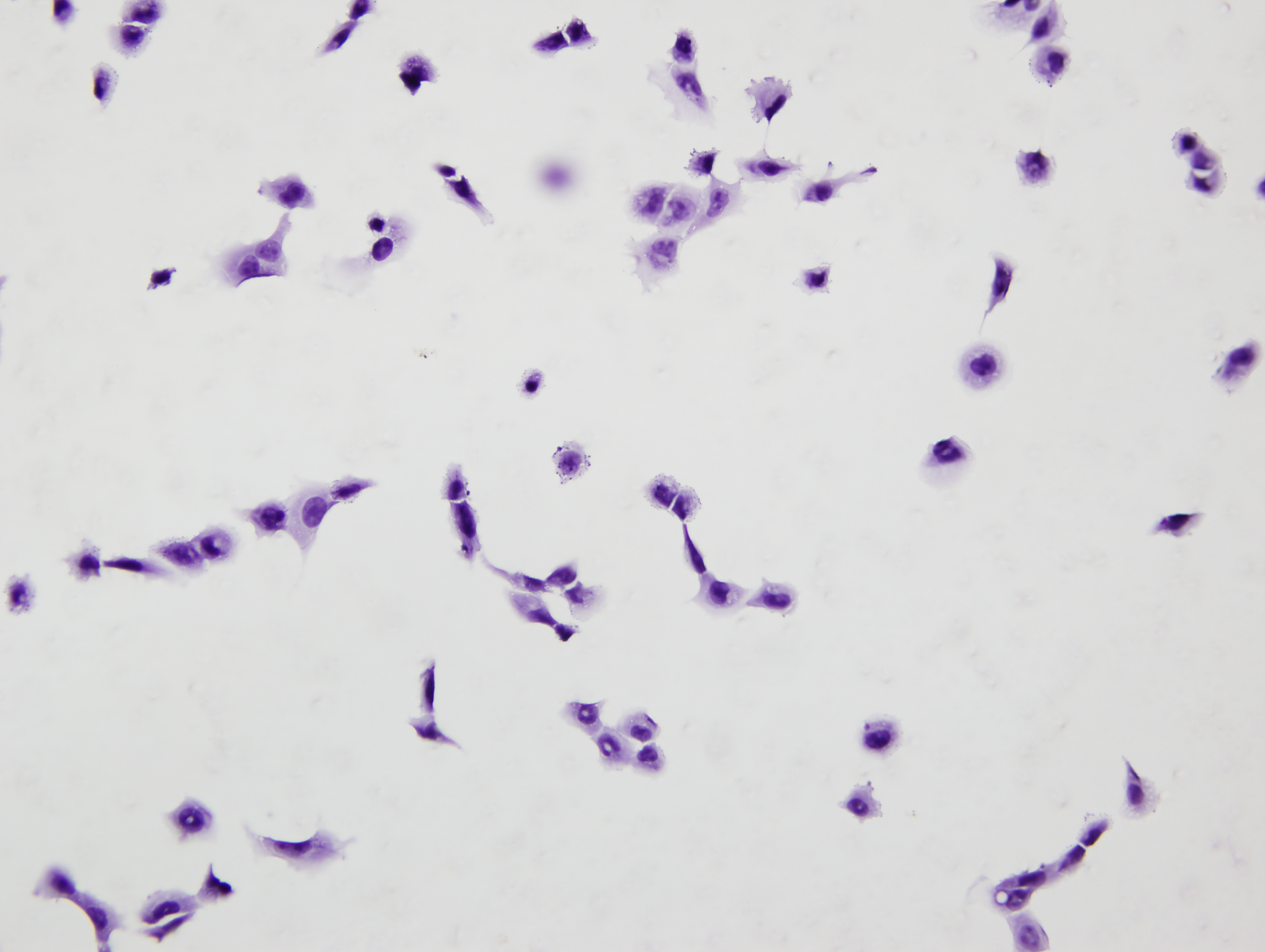

Supplement: Supplementary file 8 [file DataSheet6.ZIP › F5B right.tif]

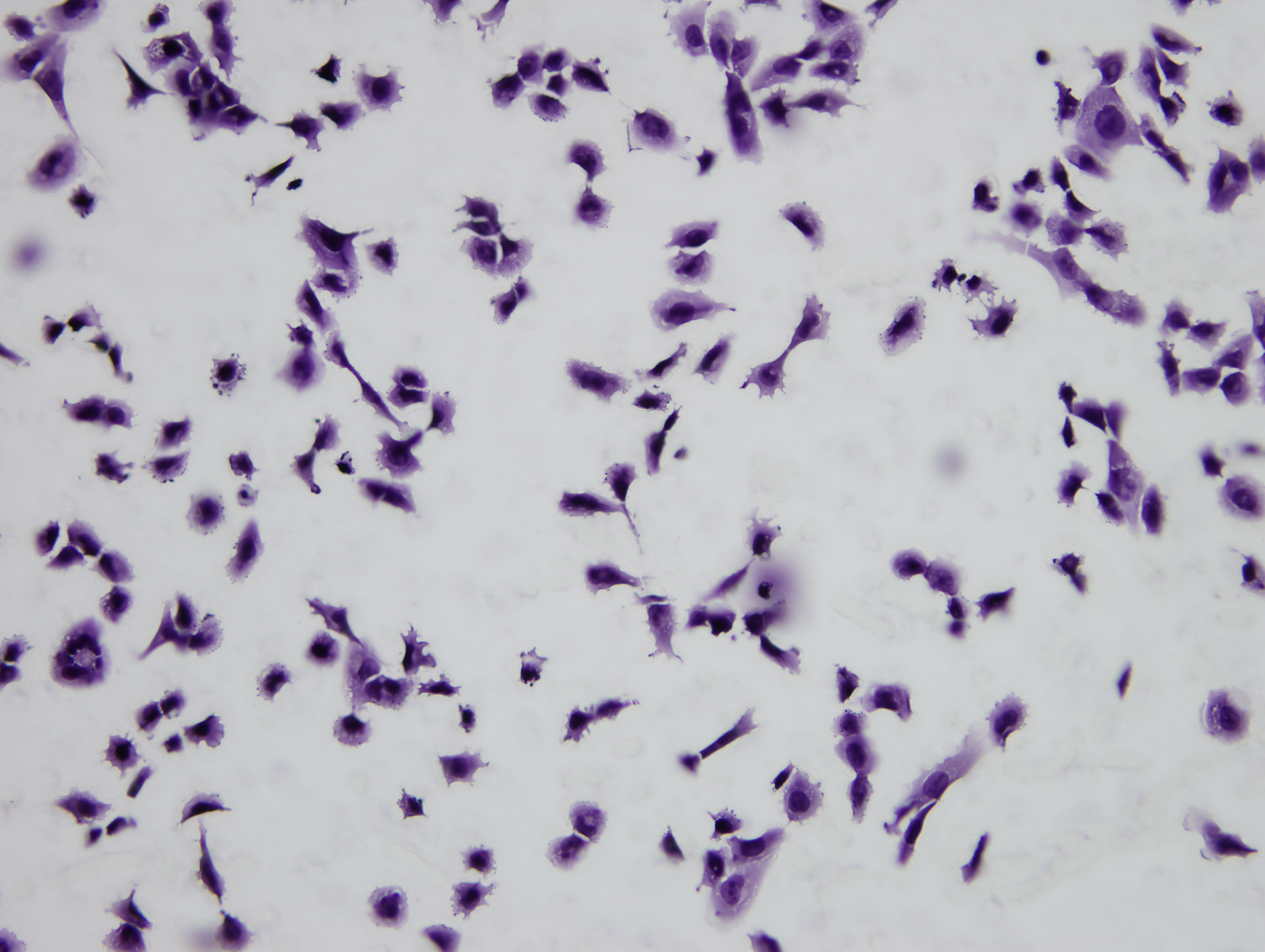

Supplement: Supplementary file 9 [file DataSheet2.ZIP › F2B left up miR-92a-3p mimic.tif]

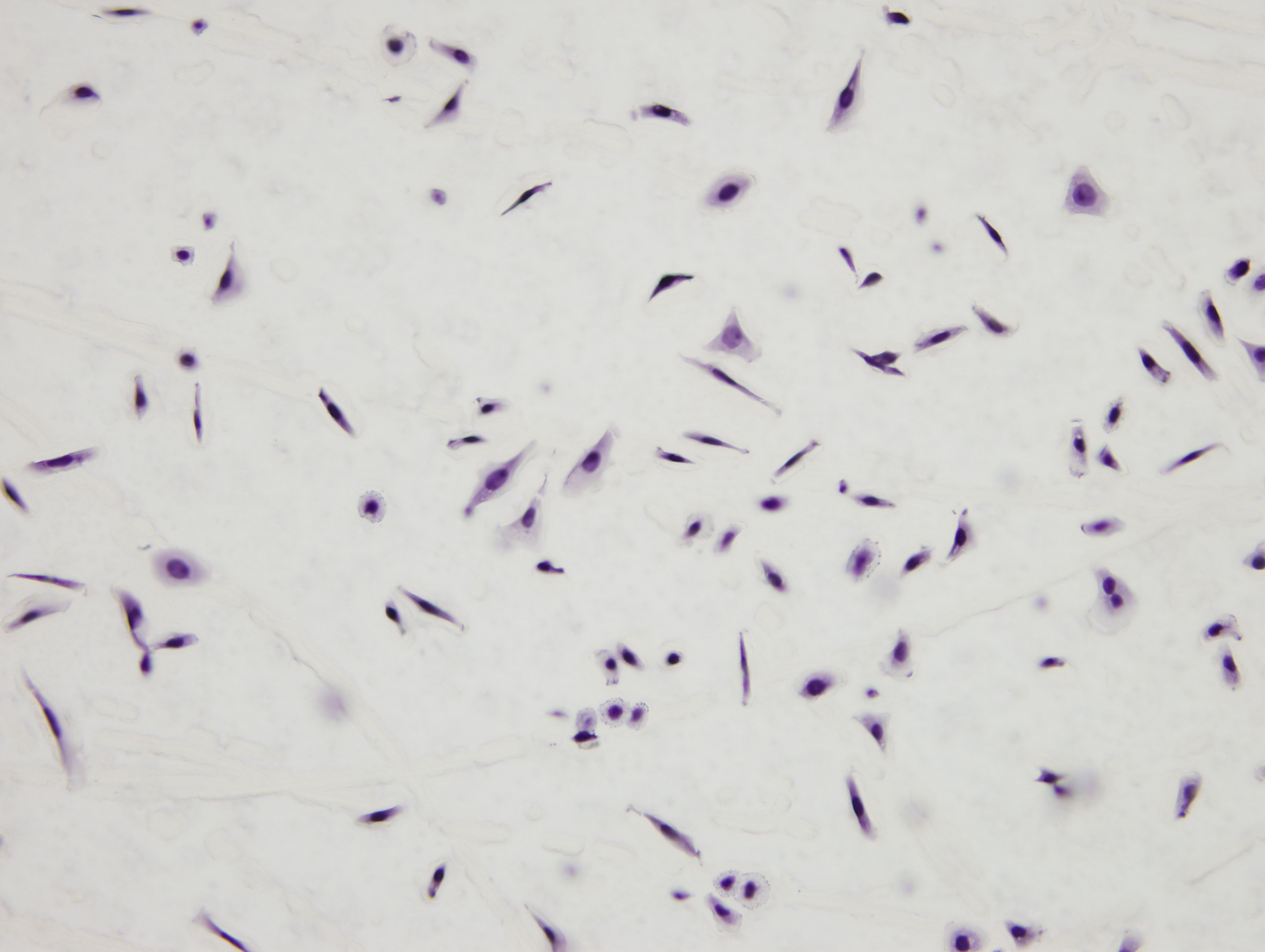

Supplement: Supplementary file 9 [file DataSheet2.ZIP › F2B right down control.tif]

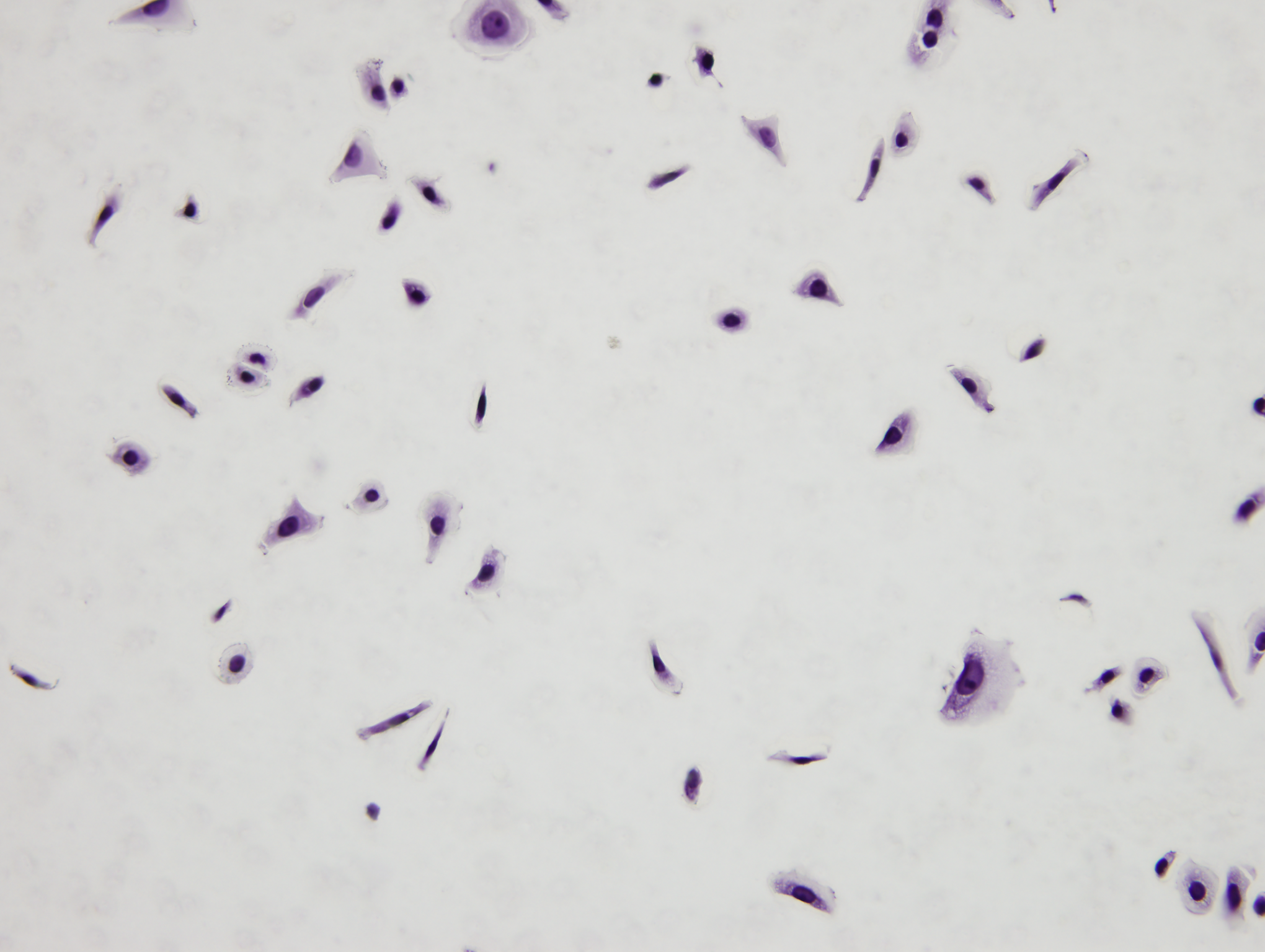

Supplement: Supplementary file 9 [file DataSheet2.ZIP › F2B right down miR-92a-3p inhibitor.tif]

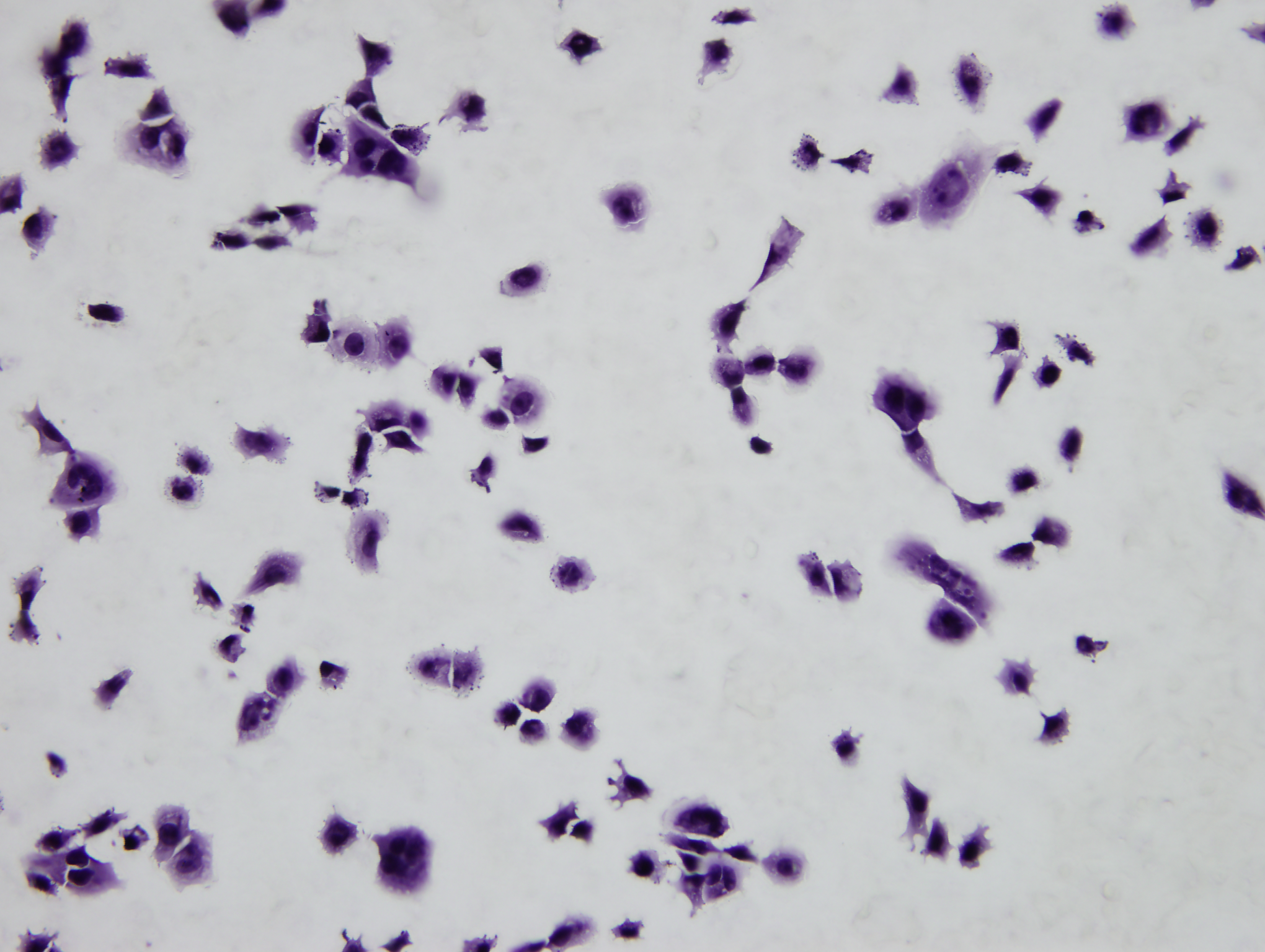

Supplement: Supplementary file 10 [file DataSheet5.ZIP › F4B right down control.tif]

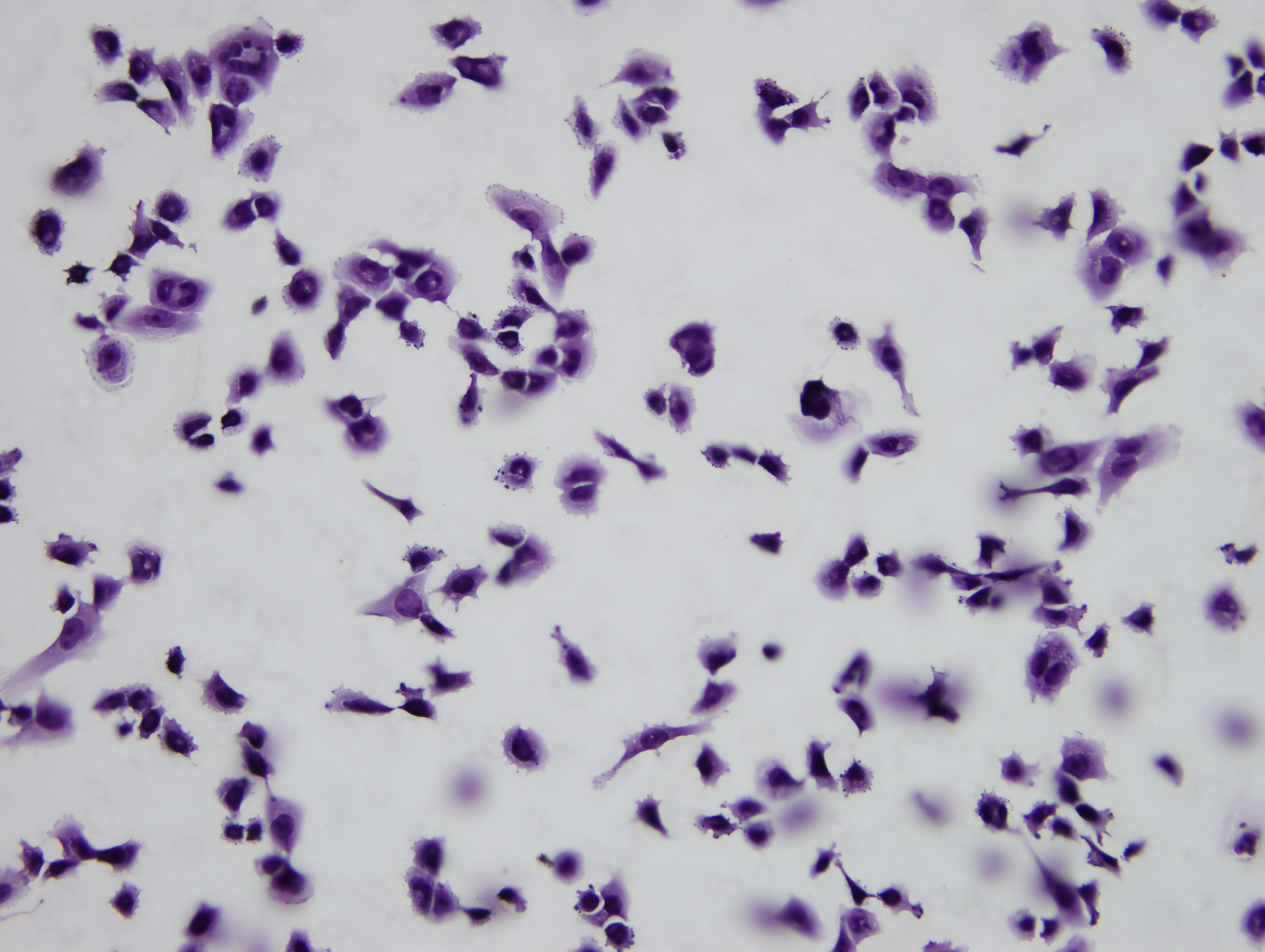

Supplement: Supplementary file 10 [file DataSheet5.ZIP › F4B right down siLats1.tif]

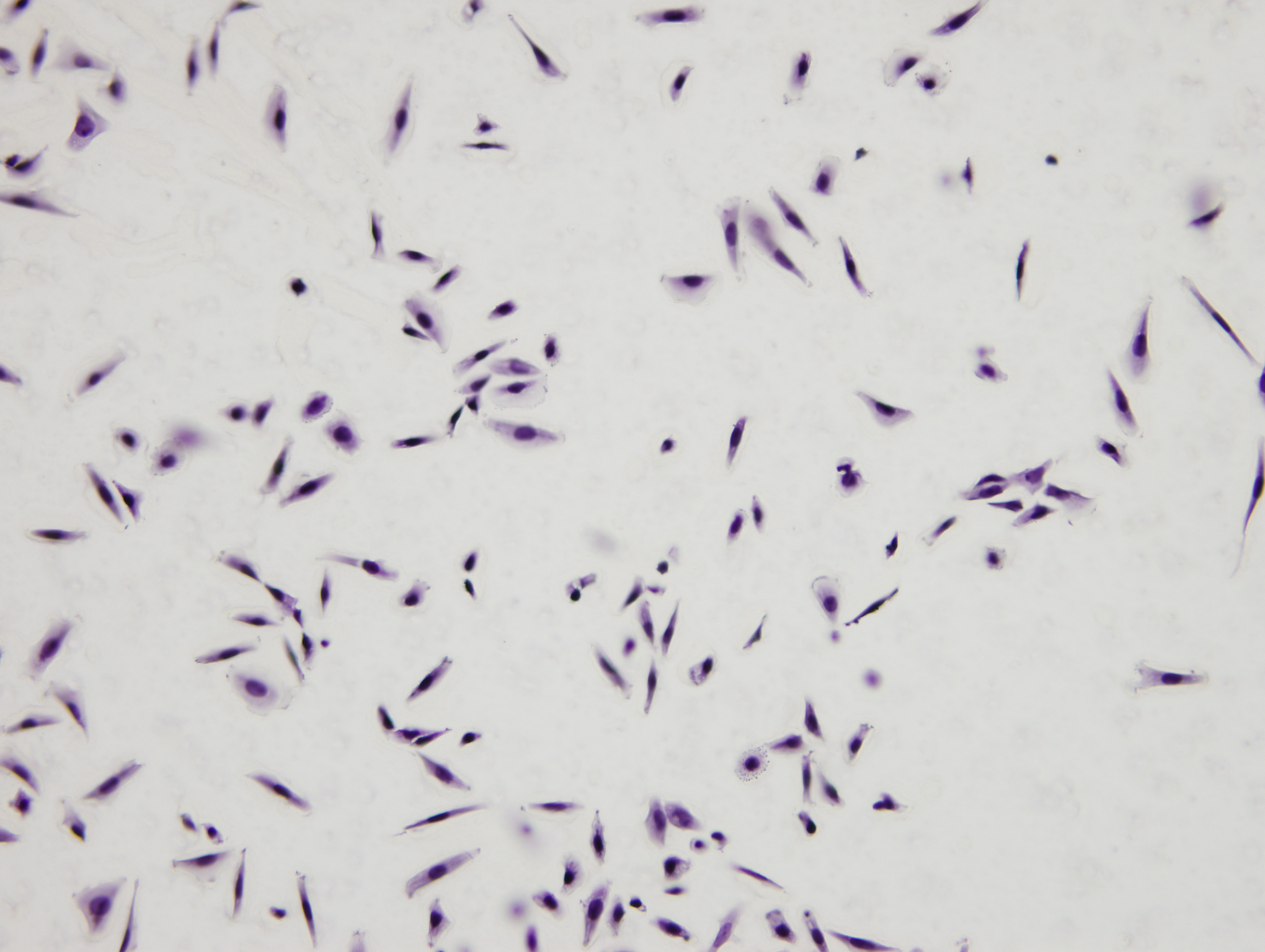

Supplement: Supplementary file 10 [file DataSheet5.ZIP › F4B right up control.tif]
